# Does standalone phacoemulsification lower intraocular pressure in glaucomatous eyes? A systematic review and meta-analysis

**DOI:** 10.1038/s41433-025-03927-7

**Published:** 2025-07-24

**Authors:** Osama Hussein, Ayman Musleh, Yara Abukhaled, Abdullah Hammad, Radi Feras, Hashem Abu Serhan

**Affiliations:** 1 School of Medicine, The University of Jordan, Amman, Jordan; 2 College of Medicine and Health Sciences, Khalifa University, Abu Dhabi, United Arab Emirates; 3 Department of Ophthalmology, Hamad Medical Corporation, Doha, Qatar

**Keywords:** Glaucoma, Optic nerve diseases, Education

## Abstract

We evaluated the effect of phacoemulsification on intraocular pressure (IOP) and the reduction of glaucoma medications in patients with glaucoma. Our protocol was registered in PROSPERO (CRD42024533437). We conducted a systematic search in PubMed, Scopus, Cochrane Central Register of Controlled Trials (CENTRAL), and Web of Science for randomized clinical trials (RCTs) with at least 10 eyes and a 12-month minimum follow-up. Risk of bias assessment was performed using the Cochrane risk-of-bias tool. A random-effects model meta-analysis was used, and heterogeneity was assessed using Cochran’s Q and I² statistics. We included 41 RCTs with a total of 2315 eyes in the phacoemulsification-alone group. In ACG patients, the pooled effect showed a percentage of IOP reduction (IOPR%) of 35.22% with a mean IOP reduction of −9.51 mmHg, *p *< 0.001 at 12 months, and an IOPR% of 27.47% with a mean reduction of −6.37 mmHg, *p *< 0.001 at 24 months. For OAG patients, the pooled effect showed an IOPR% of 18.94% with a significant absolute change of −4.35 mmHg, *p *< 0.001 at 12 months, and IOPR% of 16.95% with an absolute IOP change of −3.83 mmHg, *p *< 0.001 at 24 months. In ACG patients, medication use decreased by 67.13% at 12 months and 51.58% at 24 months (*p *< 0.001 for both), while in OAG patients, the reduction was 45.52% at 12 months and 50.19% at 24 months (*p *< 0.001). In conclusion, standalone phacoemulsification significantly lowers IOP and reduces the need for glaucoma medications in patients with glaucoma.

## Introduction

Glaucoma is a leading cause of irreversible blindness worldwide, characterized by progressive optic nerve damage often associated with elevated intraocular pressure (IOP) [1]. Over the past two decades, the prevalence of primary open-angle glaucoma (OAG) in adults has reached 2.4%, affecting ~69 million patients [1]. Although elevated IOP is not the sole cause of glaucoma, it remains the only modifiable risk factor, making it the primary target for interventions [2].

Various surgical approaches are available for glaucoma management, with trabeculectomy and glaucoma drainage implants being among the most common [3]. The choice of surgery depends on factors such as the type of glaucoma, the patient’s overall health, and associated risk factors [3]. While these surgical techniques effectively reduce IOP and control disease progression, suboptimal outcomes can still occur due to complications. Such complications include scarring and fibrosis of the filtering bleb established during trabeculectomy, as well as hypotony and tube shunt occlusion associated with glaucoma drainage devices [4, 5]. Furthermore, a recent multicentre randomized controlled trial (RCT) reported five-year cumulative failure rates of 42% for tube shunts and 35% for trabeculectomy [6].

Coexisting glaucoma and cataract are common in older individuals, particularly those over the age of 60 [7]. Given the frequent concurrence of these conditions, cataract surgery presents a unique opportunity to assess its effect on IOP changes and glaucoma progression. Several publications, including RCTs, have described the effects of cataract surgery on IOP in both glaucomatous and non-glaucomatous eyes [7–16]. While the extent of IOP reduction varies, the majority of studies have reported a postoperative decrease in IOP following cataract extraction. Additionally, a significant proportion of patients experience a reduction in the need for postoperative glaucoma medications [12–15]. These findings indicate that cataract surgery not only lowers IOP in unmedicated patients but also reduces dependence on glaucoma medications.

Our study aimed to evaluate the reliability of phacoemulsification in reducing both IOP and the need for glaucoma medications in glaucoma patients through a systematic review and meta-analysis. We analyzed the effects of phacoemulsification alone based on both absolute IOP changes and the percentage of IOP reduction (IOPR%) at 12- and 24-month follow-ups, focusing exclusively on RCTs to uphold the highest standard of evidence-based research.

## Materials and methods

We performed this meta-analysis following the Preferred Reporting Items for Systematic Reviews and Meta-Analyses (PRISMA) guidelines. The protocol was registered in the PROSPERO database (CRD42024533437).

### Eligibility criteria

We included studies that met the following criteria: 1) Reported IOP readings before and after phacoemulsification in glaucoma patients; (2) Were conducted on human subjects; (3) Used an RCT design; (4) Had a sample size of at least 10 eyes; (5) Included a follow-up period of at least 12 months; (6) Were published in the English language. Studies that did not meet these criteria were excluded.

### Search methods for identifying studies

We conducted a systematic search on the PubMed, Scopus, Cochrane Central Register of Controlled Trials (CENTRAL), and Web of Science databases on December 7, 2023, to identify relevant studies. The following keywords were used: “glaucoma” OR “open-angle” OR “angle-closure” OR “ normal tension” OR “closed angle” in combination with “phacoemulsification” OR “cataract surgery” OR “intraocular lens implantation”. No restriction was placed on the publication date or language. To ensure that no eligible studies were missed, a second search was conducted on November 19, 2024, on PubMed using additional keywords. The full search strategy is included in the Supplementary Appendix.

### Study selection

We removed duplicates and screened the studies using the Rayyan web tool [17]. Two reviewers (OH and AH) independently performed the screening according to the eligibility criteria. The screening process was conducted in three phases: title screening, abstract screening, and full-text screening. In cases of disagreement, a third reviewer (AM) was consulted.

### Data collection and quality assessment

We developed a spreadsheet on Excel (Microsoft, USA) for data extraction. The following data were extracted: First author’s name, publication year, sample size, study design, number of eyes at baseline, follow-up periods, glaucoma subtype, pre-operative IOP, post-operative IOP, and change in IOP. Details of the data extraction process are provided in the Supplementary Appendix.

For risk of bias assessment, we used the Cochrane Risk of Bias tool (RoB 2). Two independent reviewers (OH and YA) performed this assessment, and in cases of disagreement, a third reviewer (AM) was consulted.

### Data synthesis and analysis

All analyses were performed using Stata statistical software version 17 (StataCorp LLC, College Station, TX, USA).

Our primary outcome was to calculate the absolute change in IOP and IOPR% for each glaucoma subtype as well as the percentage of glaucoma medication reduction at 12- and 24-month follow-ups. Extracted or calculated data were entered into an Excel sheet, including mean change in IOP and its standard error. We performed a univariate meta-analysis of the change in IOP using a random-effects model with the DerSimonian–Laird method. Results were represented in forest plots.

To assess heterogeneity, a Galbraith plot was created. We performed separate analyses based on glaucoma subtype at different follow-up periods (6 months and last follow-up) and according to mean pre-surgical IOP. A sensitivity analysis was performed by excluding outliers identified in the Galbraith plot. To assess publication bias, we conducted three different tests for each glaucoma subtype at 12- and 24-month follow-ups: Regression-based Egger test, Begg’s test, and the nonparametric trim-and-fill analysis. A *p*-value less than 0.05 was considered statistically significant. We considered publication bias a high concern for the analyzed group if any one of the three tests was significant; if none were significant, we considered it a low concern.

## Results

### Study selection

**Fig. 1:**
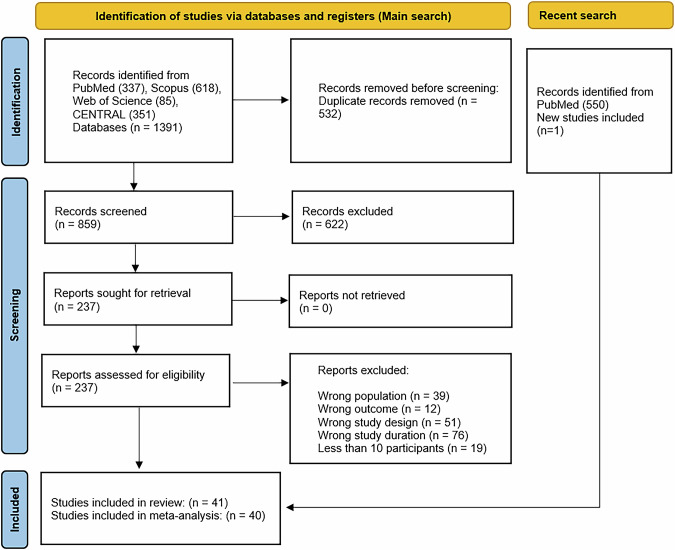
Study selection flow chart, preferred reporting items for a systematic review and meta-analysis.

Our search yielded a total of 1391 records, from which 532 duplicates were removed. After title and abstract screening, 622 studies were excluded, leaving 237 studies eligible for full-text screening. Further exclusions included: 39 studies due to an ineligible population, 12 studies for irrelevant outcomes, 51 studies for incorrect study design, 71 studies for a duration of less than 12 months, and 19 studies for having fewer than 10 participants. Additionally, one newly published study (2024) was included following a second search [18]. Figure 1 details the search and selection process.

### Study characteristics

A total of 41 studies were included, enrolling at least 2254 patients (2315 eyes) in the phacoemulsification-alone group [12–16, 18–53]. Three studies did not specify the number of patients in the phacoemulsification-alone group; data were reported based on the number of eyes rather than the number of patents [39, 47, 48]. Eighteen studies included 744 patients (745 eyes) with angle-closure glaucoma (ACG) [19–36], while twenty-three studies included more than 1510 (1570 eyes) participants with OAG [12–16, 18, 37–53].

Each included study followed patients for at least 12 months. The longest follow-up among ACG studies was 72 months (6 years), while in OAG studies, it was 60 months (5 years) [35, 51]. The mean participant age was 67 years in the ACG group and 71 years in the OAG group.

Sixteen studies reported the gender distribution of the patients in the ACG group [19–28, 30–35], while 17 studies reported gender distribution in the OAG group [12–16, 18, 38, 40, 42, 44, 46, 47, 49–53]. the ACG group included 244 males (37%) and 417 females (63%), whereas the OAG group included 628 males (47%) and 706 females (53%).

Three studies evaluated the impact of phacoemulsification alone on IOP and glaucoma medication use in patients with pseudoexfoliative glaucoma (PEXG) [40, 41, 52]. Jacobi et al. reported a mean baseline IOP of 32.0 ± 7.7 mmHg, which decreased to 18.5 mmHg at 6 months, 18.4 mmHg at 12 months, and 18.0 mmHg at 24 months postoperatively. Concurrently, glaucoma medication use reduced from a baseline of 2.2 ± 0.8 medications to 0.9 at 6 months, 0.8 at 12 months, and 1.1 at 24 months. Georgopoulos et al. reported a mean baseline IOP of 18.7 ± 1.84 mmHg, decreasing modestly to 17.1 mmHg at 6 months and further to 16.7 mmHg at 12 months. Medication use also decreased from 1.46 medications preoperatively to 1.08 at the final follow-up. In a subgroup analysis of PEXG patients, Zarei (2023) reported a mean IOP dropping from 24.1 mmHg at baseline to 13.7 mmHg at 6 months and 13.6 mmHg at 12 months. Glaucoma medication use declined from 2.47 ± 1.19 medications preoperatively to 1.33 at both 6 and 12 months.

The baseline IOP mean ranged from 15.4 to 52.6 mmHg in ACG patients and 16.65 to 32 mmHg in OAG patients. The included studies were conducted in North America, Europe, Asia, Africa, and Australia, with five studies conducted internationally [12, 14, 28, 32, 51]. All included studies involved patients who underwent lens extraction alone without additional glaucoma surgery. More details on study designs, participant numbers, follow-up periods, glaucoma types, and IOP outcomes are available in Supplementary Tables S1 and S2.

### Meta-analysis

#### Intraocular pressure reduction

The overall pooled effect demonstrated a significant mean reduction in both the IOPR% as well as the absolute change in IOP after phacoemulsification alone at 12- and 24-month follow-up periods.

##### Percentage intraocular pressure reduction (IOPR%)

**Fig. 2:**
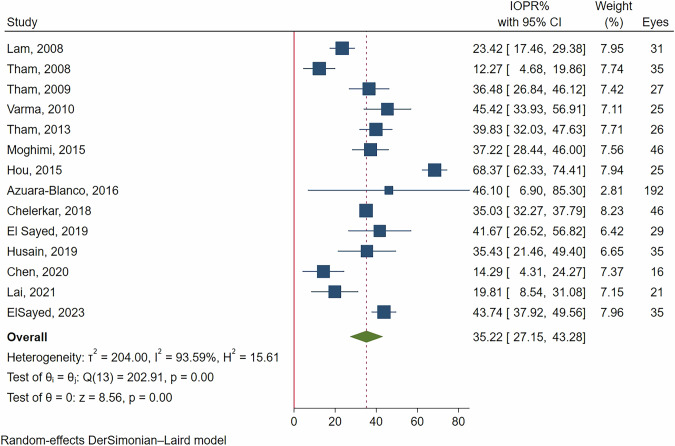
Forest plot of the IOPR% at the 12-month follow-up in patients with closed-angle glaucoma.

**Fig. 3:**
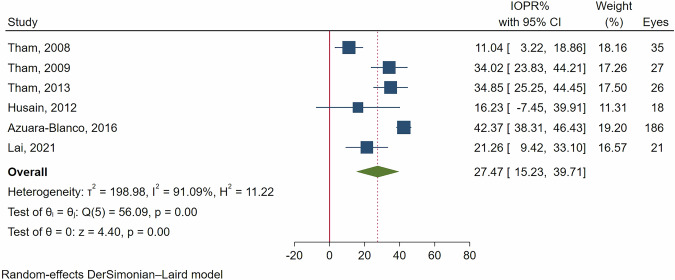
Forest plot of the IOPR% at the 24-month follow-up in patients with closed-angle glaucoma.

In ACG patients, the IOPR% at 12 months was 35.22% (95% CI: 27.15–43.28%, *p *< 0.001). By 24 months, the IOPR% decreased to 27.47% (95% CI: 15.23–39.71%, *p *< 0.001). Considerable heterogeneity was observed; with I² values of 93.59% at 12 months and 91.09% at 24 months (see Figs. 2 and 3).

**Fig. 4:**
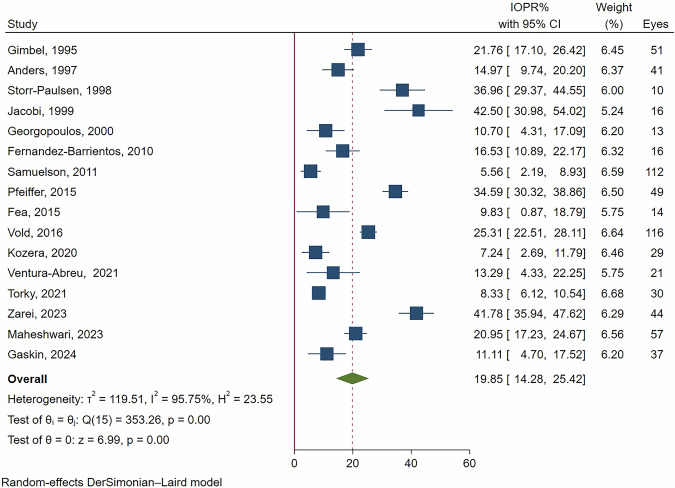
Forest plot of IOPR% at the 12-month follow-up in patients with open-angle glaucoma.

**Fig. 5:**
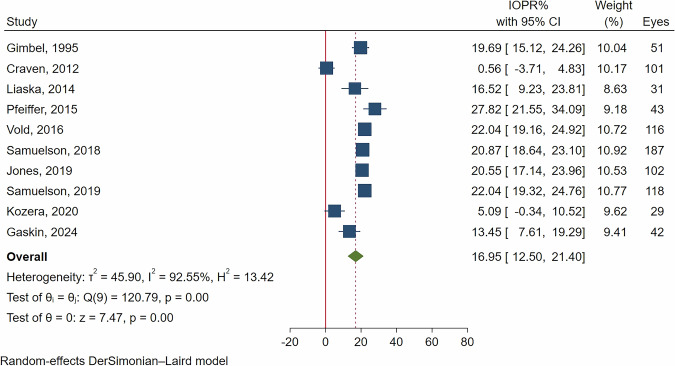
Forest plot of IOPR% at the 24-month follow-up in patients with open-angle glaucoma.

Figures 4 and 5 present the forest plots of IOPR% in OAG patients at 12- and 24-month follow-up periods. The pooled results revealed a significant reduction in IOPR%, with a 19.85% reduction (95% CI: 14.28-25.42%, *p *< 0.001) at 12 months, and a 16.95% reduction (95% CI: 12.50–21.40%, *p *< 0.001) at 24 months. Similar to the ACG group, substantial heterogeneity was noted, with I² values of 95.75% at 12 months and 92.55% at 24 months.

Given the high level of heterogeneity, a Galbraith plot was constructed to identify studies that might be contributing to this variability (see Supplementary Figs. S13–S28). Outliers falling outside the 95% confidence interval were excluded, followed by a sensitivity analysis including the remaining studies.

In the sensitivity analysis, after excluding outlier studies identified through Galbraith plots, heterogeneity was reduced to very low levels (I² = 0%–5.13%). The adjusted analysis showed statistically significant IOPR% reductions of 36.30% and 32.90% in the ACG group, and 13.31% and 15.83% in the OAG group at 12 and 24 months, respectively (see Supplementary Figs. S29–S44).

##### Absolute intraocular pressure reduction

As shown in Supplementary Figs. S1, S2, among ACG patients, the absolute reduction in IOP at 12 months was −9.51 mmHg (95% CI: −12.22 to −6.79 mmHg, *p *< 0.001), while at 24 months it was −6.37 mmHg (95% CI: −10.74 to −2.00 mmHg, *p *< 0.001). Substantial heterogeneity was observed across the included studies, with an I² of 97.54% at 12 months and 96.83% at 24 months.

Supplementary Figs. S3, S4 display the forest plots for the absolute changes in IOP among OAG patients at both 12- and 24-month follow-ups. The pooled analysis demonstrated a significant mean reduction in IOP, with an absolute change of −4.58 mmHg (95% CI: −5.95 to −3.21 mmHg, *p *< 0.001) at 12 months, and −3.83 mmHg (95% CI: −5.18 to −2.49 mmHg, *p *< 0.001) at 24 months. Similar to ACG, considerable heterogeneity was noted, with I² values of 96.72% at 12 months and 96.10% at 24 months.

In the sensitivity analysis, heterogeneity in absolute IOP reduction decreased from high to moderate levels (I² = 46–70%). The significant absolute IOP reductions remained consistent at both 12-month and 24-month follow-ups post-analysis.

#### Glaucoma medication reduction

Among ACG patients, the reduction in glaucoma medication use at 12 months was statistically significant, with a 67.13% reduction (95% CI: 53.65–80.61%, *p *< 0.001) and I² value of 85.60%. At 24 months, the reduction decreased to 51.58% (95% CI: 42.55–60.62%, *p *< 0.001), with a low heterogeneity (see Supplementary Figs. S65, S66).

Supplementary Figs. S67, S68 present the forest plots of medication reduction in OAG patients at 12- and 24-month follow-up. The pooled analysis showed a significant reduction in medication use, with a 45.52% reduction (95% CI: 35.57–55.47%, *p *< 0.001) at 12 months, and a 50.19% reduction (95% CI: 42.53–57.85%, *p *< 0.001) at 24 months. A moderate to high heterogeneity was noted, with I² values of 70.88% at 12 months and 59.99% at 24 months.

Following the exclusion of outlier studies, sensitivity analyses (Supplementary Figs. S72–S74) confirmed the significant reduction in glaucoma medication usage while also leading to a substantial decrease in heterogeneity.

#### Separate analyses

We conducted separate analyses according to presurgical IOP levels (31–23 mm Hg, 22–20 mm Hg, 19–18 mm Hg, 17–15 mm Hg, and 14–9 mm Hg) [54], and by follow-up duration (6 months and last available follow-up). Across all groups, phacoemulsification was associated with a significant reduction in both IOPR% and absolute IOP values.

##### Analysis by presurgical intraocular pressure

In ACG patients, those with a presurgical IOP of 23–31 mm Hg showed a 41.77% reduction at 12 months, while patients with a presurgical IOP of 15–17 mm Hg had an 11.55% reduction at 24 months, both with low heterogeneity. Among OAG patients, a 9.31% reduction was observed at 12 months in those with a presurgical IOP of 15–17 mm Hg, and a 21.72% reduction at 24 months in those with a presurgical IOP of 23–31 mm Hg, also with low heterogeneity. Regarding absolute IOP change, ACG patients with a presurgical IOP of 15–17 mm Hg at 24 months experienced a decrease of 1.88 mm Hg, while OAG patients with a presurgical IOP of 15–17 mm Hg at 12 months showed an absolute reduction of 1.63 mm Hg. Additionally, OAG patients with a presurgical IOP of 23–31 mm Hg had an absolute reduction of 5.45 mm Hg at 24 months, all with low heterogeneity. The remaining metanalyses also demonstrated significant reductions, although heterogeneity ranged from moderate to high (see Supplementary Figs. S45–S64).

##### Analyses by follow-up duration

A meta-analysis was also conducted based on follow-up periods at 6 months and the last available follow-up (Supplementary Figs. S4–S12). The results demonstrated a significant IOP reduction following phacoemulsification but with high heterogeneity. To address the issue, a Galbraith plot and sensitivity analysis were conducted. The sensitivity analysis for the 6-month period for both OAG and ACG showed low heterogeneity. For the last follow-up, moderate heterogeneity was observed in the absolute change in IOP, while low heterogeneity was observed in the IOPR% after the sensitivity analysis (see Supplementary Appendix).

#### Publication bias

For ACG at 12 and 24 months, both Egger’s test for small-study effects and Begg’s test produced *p*-values greater than 0.05, suggesting that publication bias is unlikely. Additionally, the non-parametric trim-and-fill analysis showed no imputed studies, supporting the absence of publication bias. For OAG at 12 months, Egger’s test revealed significant small-study effects, Whereas Begg’s test did not, and non-parametric trim-and-fill analysis showed no imputed studies. For OAG at 24 months, all tests indicated a low risk of publication bias (see Supplementary Appendix).

### Risk of bias and applicability

**Fig. 6:**
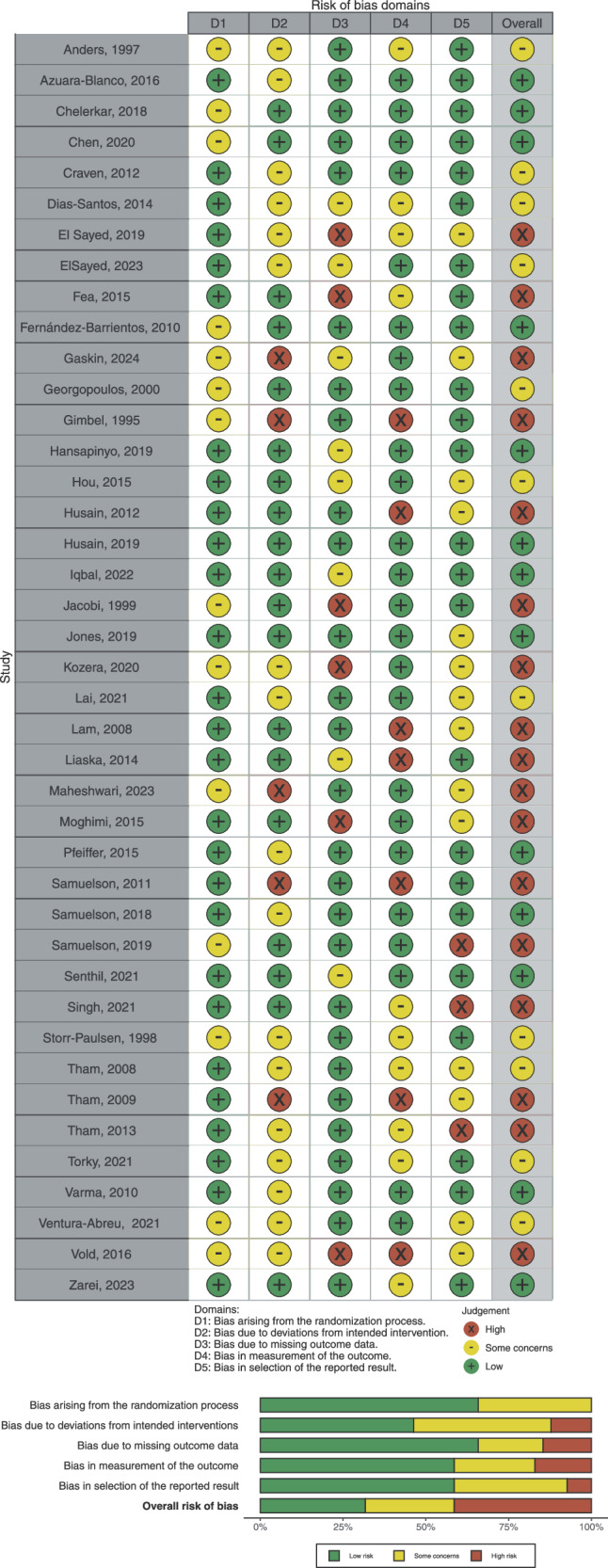
Risk of bias summary.

The risk of bias and applicability concerns for each individual domain are presented in Fig. 6. Risk of bias and applicability concerns were applied to five key domains:

1. Randomization process: Out of the 41 included studies, 14 (34.1%) showed some concerns in the randomization process [15, 16, 18, 29, 33, 37–42, 47, 48, 53], while the remaining 27 (65.9%) studies had low risk of bias [12–14, 19–28, 30–32, 34–36, 43–46, 49–52].
2. Deviations from intended interventions: Five of the 41 studies (12.2%) had a high risk of deviation from intended interventions [13, 18, 21, 37, 53], 17 (41.5%) showed some concerns regarding bias [12, 14, 15, 20, 22, 23, 25, 28, 31, 34, 36, 38, 39, 43, 47, 48, 50], and the remaining 19 (46.3%) studies showed low risk of bias [16, 19, 24, 26, 27, 29, 30, 32, 33, 35, 40–42, 44–46, 49, 51, 52].
3. Missing outcome Data: Six of the 41 studies (14.6%) did not include all the participant’s data and showed high risk of bias [15, 26, 31, 40, 45, 47], 8 (19.5%) showed some concerns [18, 25, 27, 30, 35, 36, 44, 51], and the remaining 27 studies (65.9%) showed low risk of bias [12–14, 16, 19–24, 28, 29, 32–34, 37–39, 41–43, 46, 48–50, 52, 53].
4. Measurement of the outcome: Seven (17.1%) studies had a high risk of bias due to inadequate blinding [13, 15, 19, 21, 24, 37, 44], 10 (24.4%) of the studies showed some concerns [20, 23, 25, 31, 38, 39, 45, 49, 50, 52], while the remaining 24 (58.5%) studies appropriately measured the outcome [12, 14, 16, 18, 22, 26–30, 32–36, 40–43, 46–48, 51, 53].
5. Selection of the reported results: Three of the 41 studies (7.3%) had high risk of bias in the selection of reported results [16, 23, 49], 14 (34.1%) showed some concerns [15, 18–21, 24, 26, 27, 31, 34, 46–48, 53], and the remaining 24 studies (58.5%) had low risk of bias when reporting results [12–14, 22, 25, 28–30, 32, 33, 35–45, 50–52].

Overall, 17 of the 41 studies (41.5%) were classified as having a high risk of bias [13, 15, 16, 18, 19, 21, 23, 24, 26, 31, 37, 40, 44, 45, 47, 49, 53], 11 studies (26.8%) showed some concerns [20, 25, 27, 34, 36, 38, 39, 41, 43, 48, 50], and the remaining 13 studies (31.7%) had a low risk of bias [12, 14, 22, 28–30, 32, 33, 35, 42, 46, 51, 52].

## Discussion

To our knowledge, this is the first systematic review and meta-analysis evaluating both the absolute change in IOP and the IOPR% after phacoemulsification, along with percentage of glaucoma medication reduction in OAG and ACG patients, using data exclusively from RCTs. Overall, phacoemulsification resulted in a significant reduction in absolute change in IOP in both OAG and ACG patients. Additionally, the IOPR% in ACG patients was higher than OAG patients across most follow-up periods. There was also a significant reduction in glaucoma medication use in all groups following phacoemulsification alone.

Previous research has explored the effects of phacoemulsification on IOP in glaucoma patients. A Cochrane review by Ong et al. assessed the effect of lens extraction in the treatment of chronic primary ACG. They found that phacoemulsification may not significantly lower IOP compared to standard care; however, it does offer benefits in reducing medication dependency and slowing visual field progression [55]. Benekos et al. explored the effect of phacoemulsification on IOP in patients with OAG and reached a conclusion similar to ours, finding that phacoemulsification alone significantly reduces IOP in OAG patients at 1-year follow-up and beyond [56]. However, unlike our approach, they appeared to include studies with outcomes measured at different time points within the same analysis.

Additionally, their review included fewer studies, primarily due to limited inclusion criteria. For example, they excluded the study by Craven et al. [43] and Pfeiffer et al. [12] because it involved patients with pigmentary glaucoma. We chose to include these studies, as pigmentary glaucoma is considered a subtype of OAG. They also excluded Iqbal et al. [51] on the grounds that it was a post-trial follow-up of the Horizon study. While we recognized this concern, as mentioned in our Supplementary Appendix, we included it because it provided valuable long-term follow-up data (5 years) while also avoiding duplicate reporting of the 2-year follow-up data already reported by Samuelson et al. [14].

Masis et al. conducted a meta-analysis on the role of phacoemulsification in glaucoma therapy, incorporating data from both non-randomized and randomized studies. Their findings indicated that for OAG patients, phacoemulsification resulted in an average IOP reduction of −2.7 mmHg at 12 months and beyond [7]. The effect was more pronounced in ACG patients with a reduction of up to −6.4 mmHg at 12 months and beyond [7]. Similarly, a meta-analysis by Pasquali et al. assessed IOP reduction at different time points for both OAG and ACG patients, reporting reductions at 6, 12, and 24-month follow-ups. However, their analysis also pooled data from both non-randomized and randomized studies, which may have introduced variability in their results [57].

Multiple mechanisms are thought to contribute to the IOP-lowering effect of phacoemulsification, including widening of the iridocorneal angle and mechanical changes associated with washout of the trabecular meshwork [58]. A separate meta-analysis found that phacoemulsification not only lowers IOP but also reduces the average number of glaucoma medication required, with the most significant effect occurring within the first one to two years postoperatively, which aligns with our findings [8]. We observed that while IOP significantly decreases after surgery, the effect diminishes over time. Nonetheless, even temporary IOP reductions may delay the need for more invasive glaucoma surgeries, emphasizing the need for further research to determine the long-term benefits of phacoemulsification.

We limited inclusion to RCTs because randomization minimizes confounding and provides the highest‑certainty estimate of the causal impact of phacoemulsification on IOP, whereas observational designs are more susceptible to selection bias, secular trends, and postoperative management differences.

Due to the high variability in IOP changes across studies, the exact amount of reduction after phacoemulsification cannot be reliably predicted for an individual patient. Factors such as baseline IOP, patient demographics, and type of glaucoma all influence the extent of IOP reduction.

To investigate the impact of heterogeneity on our findings, we generated Galbraith plots, which plot each study’s standardized effect size against the inverse of its standard error. Studies falling outside the standard-error envelope were classified as outliers and subsequently removed together. After excluding these outliers, the meta-analysis was repeated. The revised pooled estimate remained within the original confidence interval, and heterogeneity significantly decreased, confirming that our overall conclusions were robust and not driven by these outlier studies [59].

One of the primary limitations of our study is the lack of RCTs comparing glaucoma patients who underwent phacoemulsification versus those who did not, likely due to ethical concerns associated with conducting such head-to-head studies. Another limitation is the lack of a “washout” period before surgery in many of the included studies, which is essential for determining baseline IOP and preventing overestimation of phacoemulsification effect. Additionally, inconsistencies in how IOP changes were reported make direct comparisons challenging. Establishing standardized guidelines for reporting glaucoma surgery outcomes would improve the quality and comparability of future research.

Despite the high variability between individual studies, the overall trend in our analysis clearly indicates that phacoemulsification significantly lowers IOP and reduces the need for glaucoma medications in glaucoma patients, with effects persisting for at least 12 months postoperatively. To establish a gold standard for glaucoma treatment, further research is needed on long-term outcomes, particularly regarding the combination of phacoemulsification with minimally invasive glaucoma surgeries (MIGS). Future research should also prioritize patient-centered outcomes, such as quality of life improvements and long-term vision preservation.

## Conclusion

Standalone phacoemulsification significantly lowers IOP and reduces the need for glaucoma medications in glaucoma patients. However, high heterogeneity in some outcomes may limit the interpretations of our findings. Future research should focus on identifying patient-specific factors that predict IOP reduction after phacoemulsification, which could help guide surgical decision- making and optimize treatment strategies.

Supplemental material is available at Eye’s website.

## Summary

### What is known about this top

- Coexisting glaucoma and cataract are common in older individuals, particularly those over the age of 60.
- Given the frequent concurrence of these conditions, cataract surgery presents a unique opportunity to assess its effect on IOP changes and glaucoma progression.

### What this study adds

- Our study aimed to evaluate the reliability of phacoemulsification in reducing both IOP and the need for glaucoma medications in glaucoma patients through a systematic review and meta-analysis.
- Standalone phacoemulsification significantly lowers IOP and reduces the need for glaucoma medications in patients with glaucoma.

## Supplementary information

SUPPLEMENTAL MATERIAL

Table 1S

Table 2S
