# Supplementary material for: Does standalone phacoemulsification lower intraocular pressure in glaucomatous eyes? A systematic review and meta-analysis
Source: Eye (Lond). 2025 Jul 24;39(13):2518–26. doi: 10.1038/s41433-025-03927-7 (PMC12402476; doi:10.1038/s41433-025-03927-7)
Supplement: Supplementary file 1 — SUPPLEMENTAL MATERIAL [file 41433_2025_3927_MOESM1_ESM.docx]

Supplementary materials

**Search Strategy:**

**Main Search, 07/12/2023**

PubMed:

("phacoemulsification"[Title/Abstract] OR "cataract surgery"[Title/Abstract] OR "Phaco"[Title/Abstract] OR "intraocular lens implantation"[Title/Abstract] OR "IOL implantation"[Title/Abstract]) AND (glaucoma[Title/Abstract] OR "Open angle"[Title/Abstract] OR "Angle closure"[Title/Abstract] OR "Normal Tension"[Title/Abstract] OR "Closed angle"[Title/Abstract]) AND (clinicaltrial[Filter] OR randomizedcontrolledtrial[Filter])

337 results

Scopus

*Advanced Search*: (TITLE-ABS-KEY (("phacoemulsification" OR "cataract surgery" OR "Phaco" OR "intraocular lens implantation" OR "IOL implantation") AND (glaucoma OR "open angle" OR "angle closure" OR "normal tension" OR "closed angle")) AND TITLE-ABS-KEY ("randomized controlled trial" OR "controlled clinical trial"))

618 results

Cochrane Central Register of Controlled Trials (CENTRAL)

Title Abstract Keyword: ("phacoemulsification" OR "cataract surgery" OR "Phaco" OR "intraocular lens implantation" OR "IOL implantation") AND (glaucoma OR "Open angle" OR "Angle closure" OR "Normal Tension" OR "Closed angle") AND ("randomized controlled trial" OR "controlled clinical trial")

351 results

Web of science

*All fields*: (("phacoemulsification" OR "cataract surgery" OR "Phaco" OR "intraocular lens implantation" OR "IOL implantation") AND (glaucoma OR "Open angle" OR "Angle closure" OR "Normal Tension" OR "Closed angle") AND ("randomized controlled trial" OR "controlled clinical trial"))

85 results

**Second search, 19/11/2024**

**PubMed:**

("glaucoma"[MeSH Terms] OR "glaucoma, open angle"[MeSH Terms] OR "ocular hypertension"[MeSH Terms] OR "glaucoma"[Title/Abstract] OR "Open angle"[Title/Abstract] OR "Angle closure"[Title/Abstract] OR "Normal Tension"[Title/Abstract] OR "Closed angle"[Title/Abstract]) AND ("Phacoemulsification"[MeSH Terms] OR "Cataract Extraction"[MeSH Terms] OR "Phacoemulsification"[Title/Abstract] OR "phakoemulsification"[Title/Abstract] OR "cataract surgery"[Title/Abstract] OR "Lens extraction"[Title/Abstract] OR "Phaco"[Title/Abstract] OR "intraocular lens implantation"[Title/Abstract] OR "IOL implantation"[Title/Abstract]) AND (clinicaltrial[Filter] OR randomizedcontrolledtrial[Filter])

550 results

**Data extraction details**

For each study, the extracted or calculated data were entered into an Excel sheet along with the mean change in IOP and its standard error.

If studies did not report IOP change directly, but reported only IOP in the pre-operative and at the follow-up period. The mean IOP change can be estimated simply by the difference of IOP value before and after phacoemulsification, while the SD of the IOP change is estimated by [(Ref):](https://handbook-5-1.cochrane.org/0_2_how_to_cite_the_handbook.htm)

$$SD(\Delta change)= \sqrt{(SD(pre)^2 + SD(post)^2 - (2 \times Corr \times SD(pre) \times SD(post)))}$$

Corr, is the average of correlation coefficient values computed from studies reported full details of mean and standard deviation for all baseline, final and change in IOP values. The correlation between the pre-and-post measurements, was set to the median of the correlations in the studies reporting the full set of metrics, which was, 0.211566092

To calculate both the percentage reduction in intraocular pressure (IOP) and the standard error (SE) of percentage reduction for studies we used the following formulas ([Ref](https://doi.org/10.1371/journal.pone.0131770)):

IOPR% = (IOPR / IOP baseline) × 100

SE IOPR% = SE IOPR / IOP baseline * 100

If median and IQR were provided, we estimated the mean and SD using the Cochrane collaboration formulas. If median and range were reported we estimated mean and SD using formula by Hozo et al .

The standard deviation was converted into standard error for analysis using the following equation:

$$SE=\frac{SD}{\sqrt{Sample size}}$$

If studies explored the effect of washout period on intraocular pressure, data extraction was conducted as follows:

All Washout Periods Present:

If washout values are presented for all periods, we included the washout values for all periods.

Some Washout Periods Present:

1. If all reported washout periods match the periods we want to calculate, we chose those washout periods.
2. If only some of the desired washout periods are present and the other period's mean and standard deviation (SD) cannot be calculated, we included the available washout periods since the other periods could not be extracted or calculated.
3. If some washout periods are present but some of the desired periods are in non-washout form and we can calculate the mean and SD for them to be included in the analysis, we chose the non-washout periods for consistency.

* In Fea, 2015, we included the results of baseline from a previous study (Fea, 2010) and the 12 months and 48 months from the recent study 2015.

* For Jacobi, 1999, the 2.5-year follow-up results were excluded from the IOP analysis because the number of patients was fewer than 10, instead we used the 2-year data.

* In Ventura‑Abreu, 2021, we included the calculated raw mean intraocular pressure reduction values in the meta-analysis, rather than the GEE-adjusted estimates, to ensure consistency with the other studies included in the analysis.

* Iqbal (2022) is a follow-up study to Samuelson (2018). To avoid duplication, we did not include the same 2-year follow-up results from the study in the 24-month analysis

**Sub-group Analysis Based on Follow-up:**

*
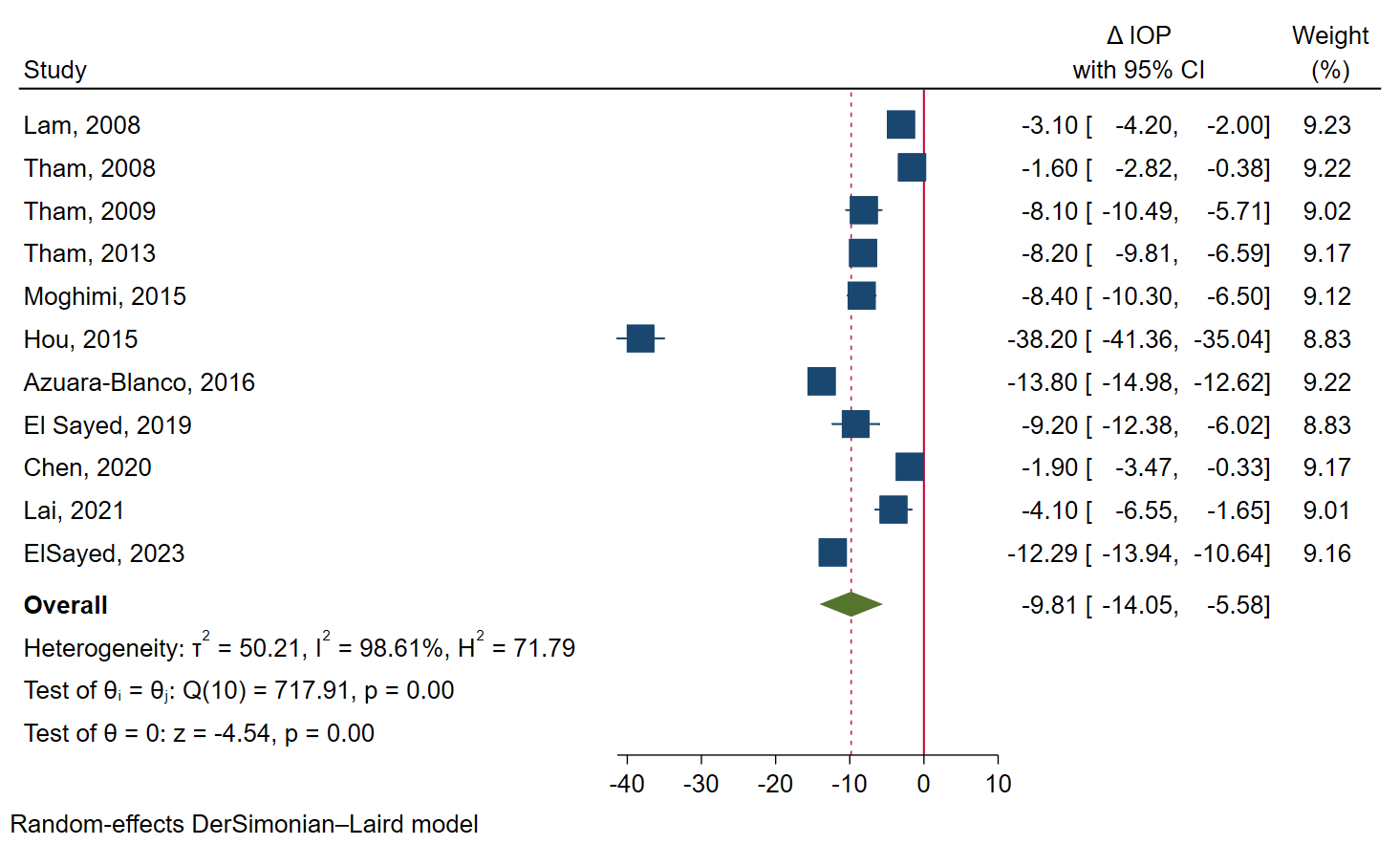
*

**Fig. S1.** Forest plot of the changes in IOP at the 6-month follow-up in patients with closed-angle glaucoma


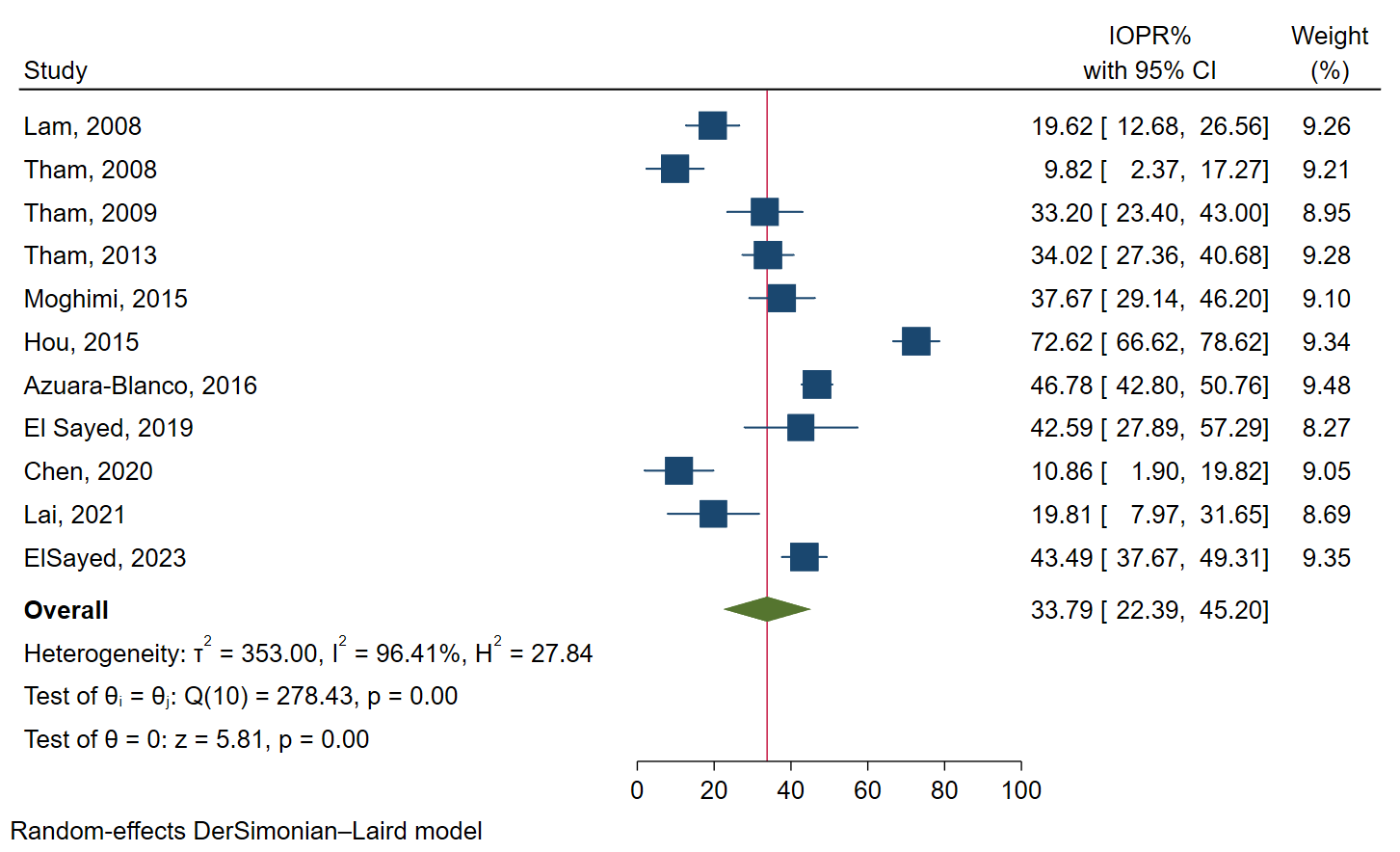


**Fig. S2.** Forest plot of the percentage of IOP reduction at the 6-month follow-up in patients with closed-angle glaucoma

*
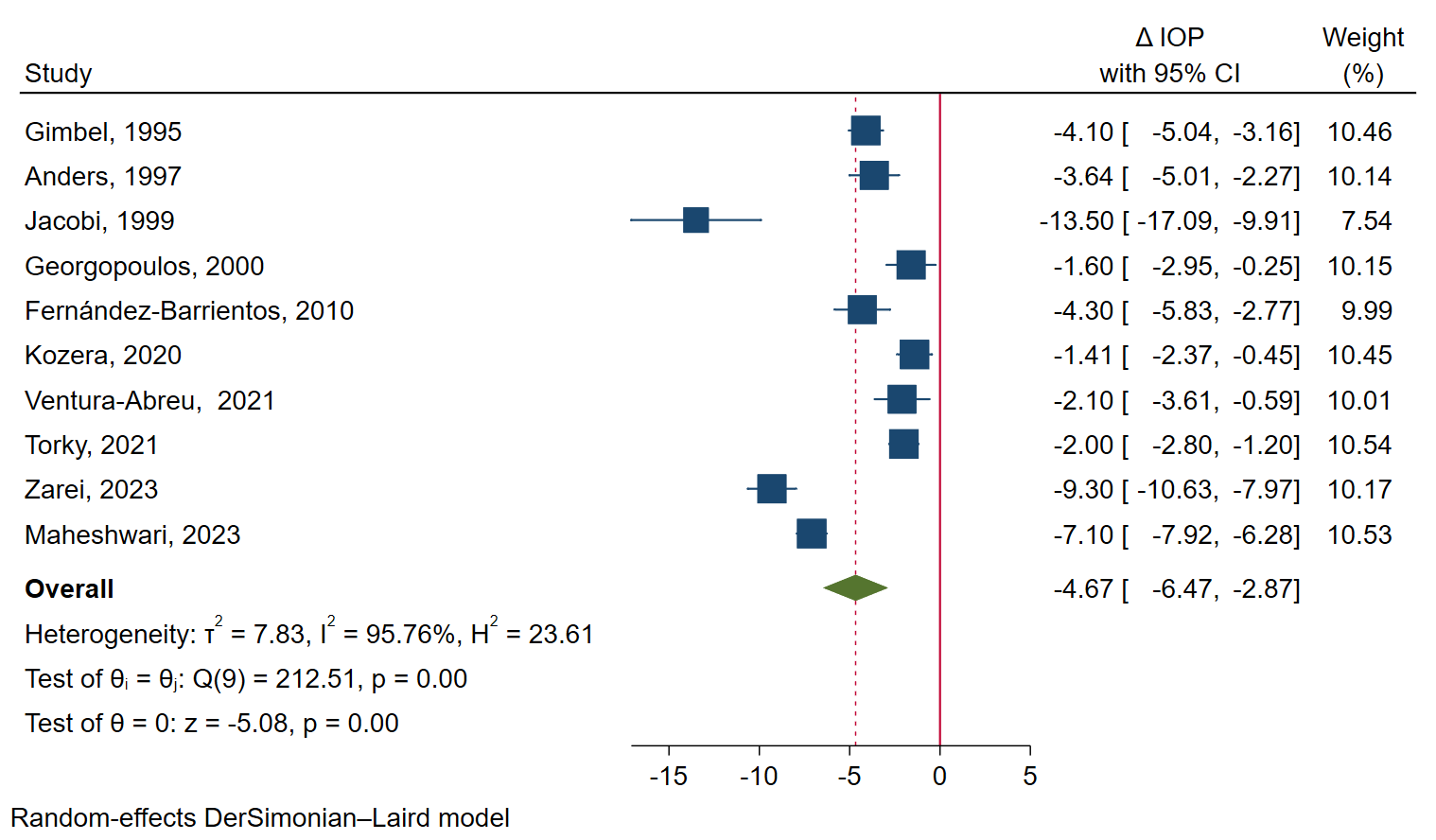
*

**Fig. S3.** Forest plot of the changes in IOP at the 6-month follow-up in patients with open-angle glaucoma


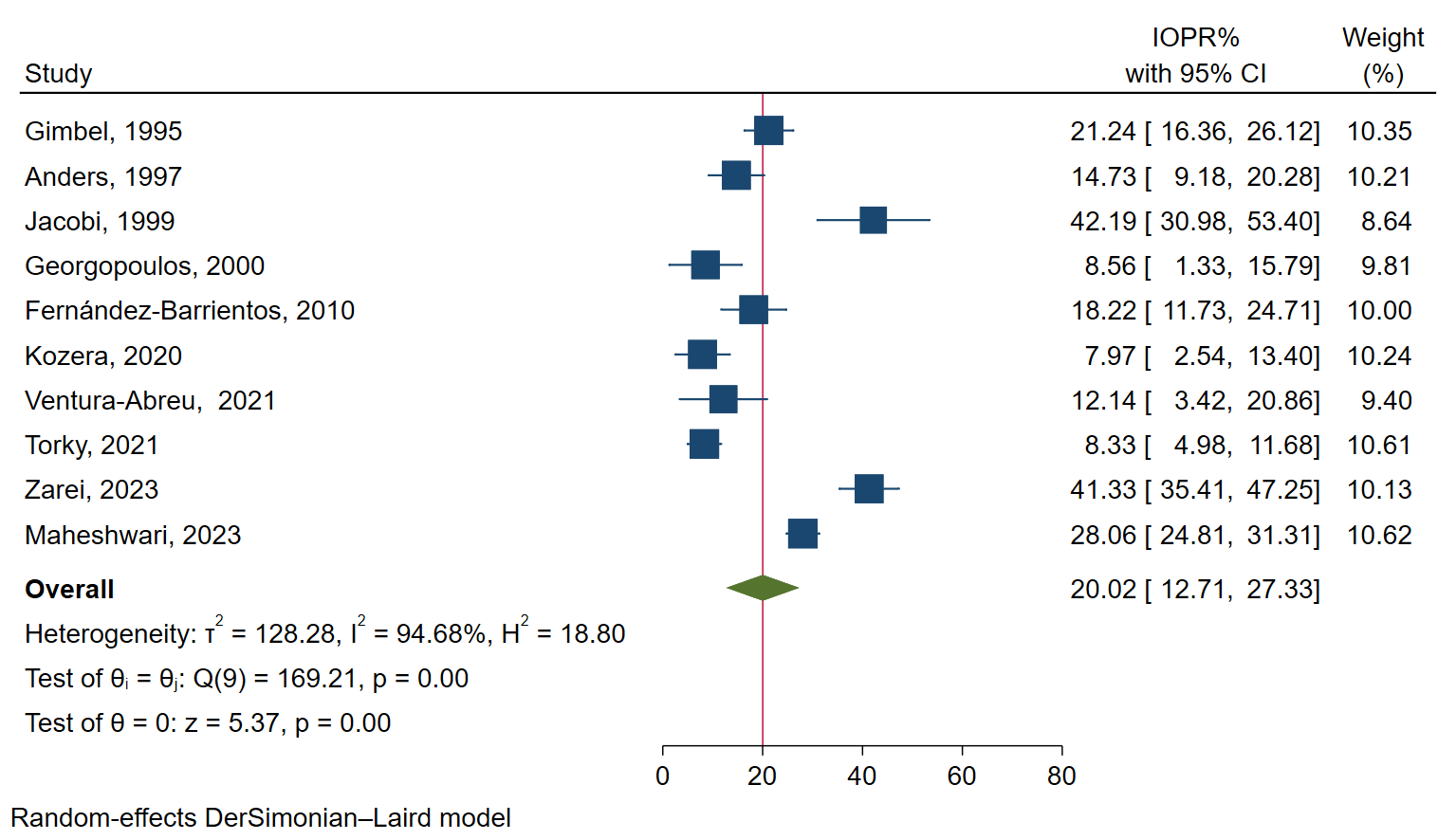


**Fig. S4.** Forest plot of the percentage of IOP reduction at the 6-month follow-up in patients with open-angle glaucoma


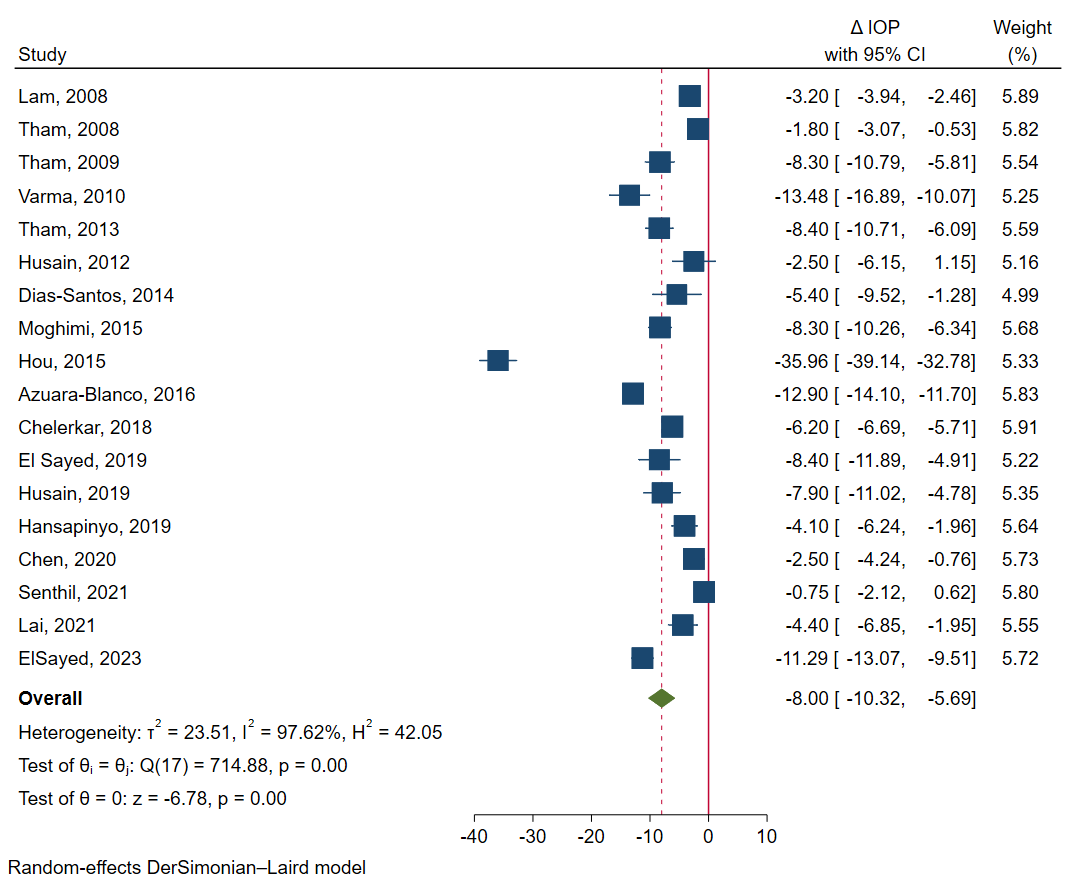


**Fig. S5.** Forest plot of the changes in IOP at the last follow-up in patients with closed-angle glaucoma


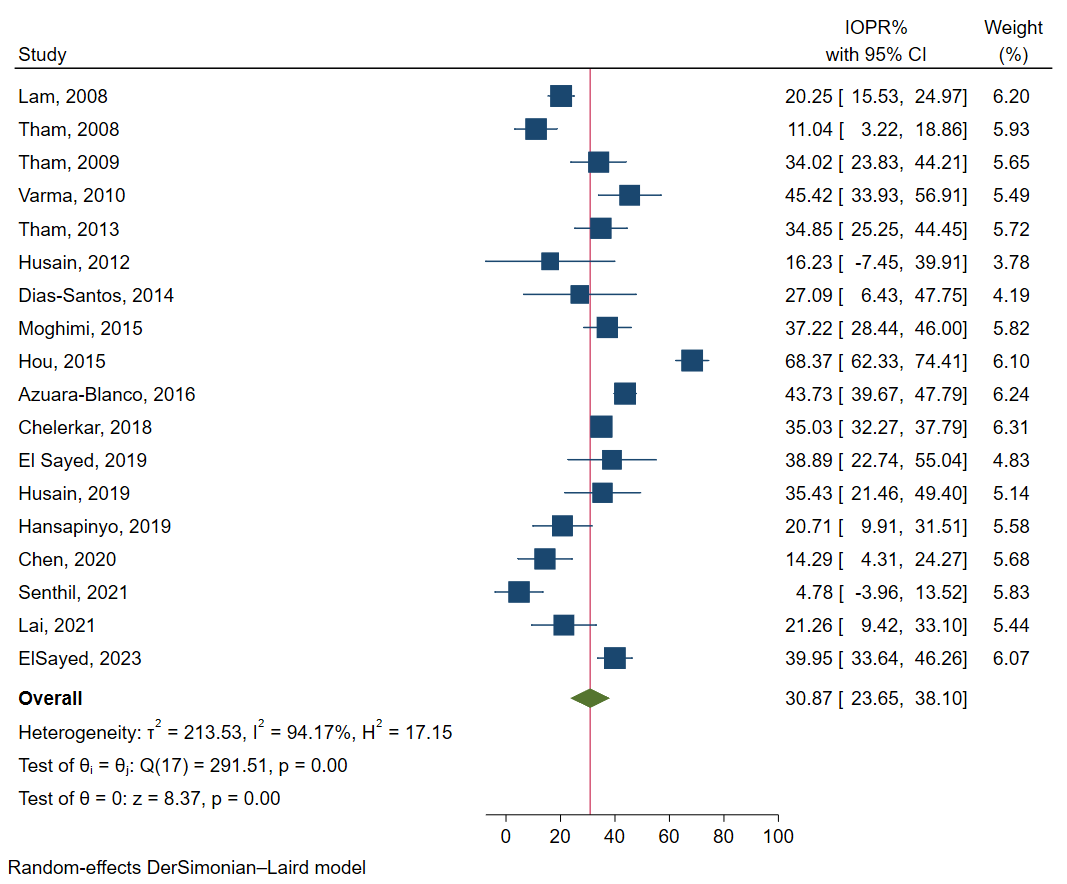


**Fig. S6.** Forest plot of the percentage of IOP reduction at the last follow-up in patients with closed-angle glaucoma

**
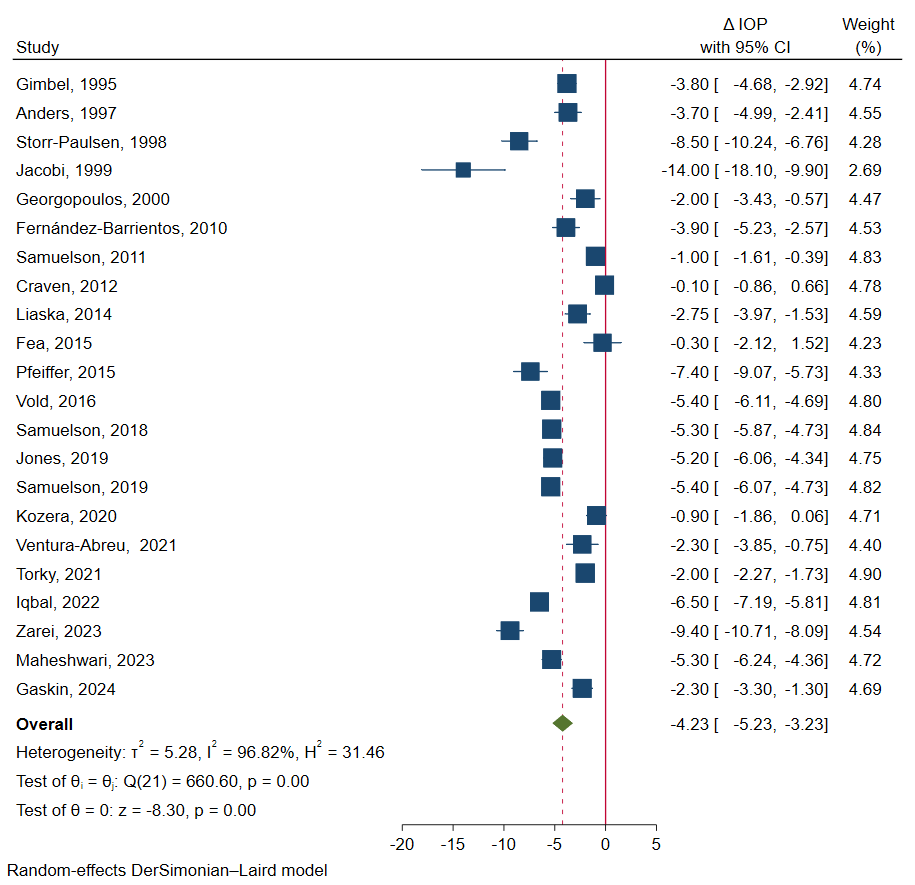
**

**Fig. S7.** Forest plot of the changes in IOP at the last follow-up in patients with open-angle glaucoma


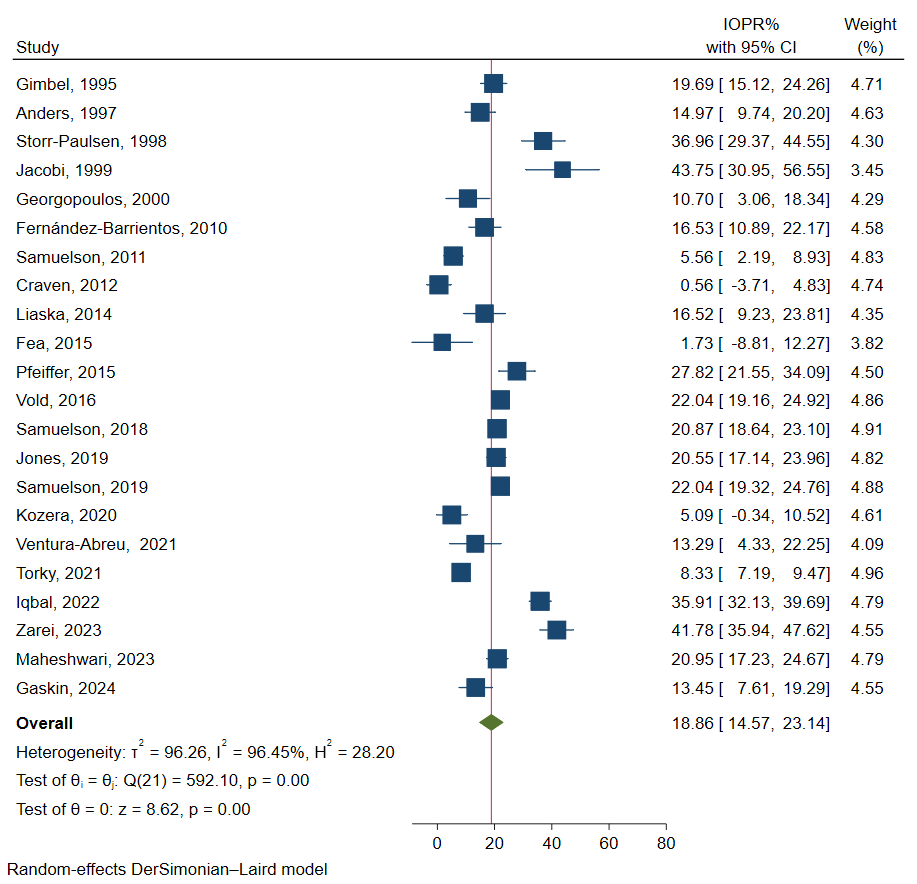


**Fig. S8.** Forest plot of the percentage of IOP reduction at the last follow-up in patients with open-angle glaucoma

**Galbraith plots:**


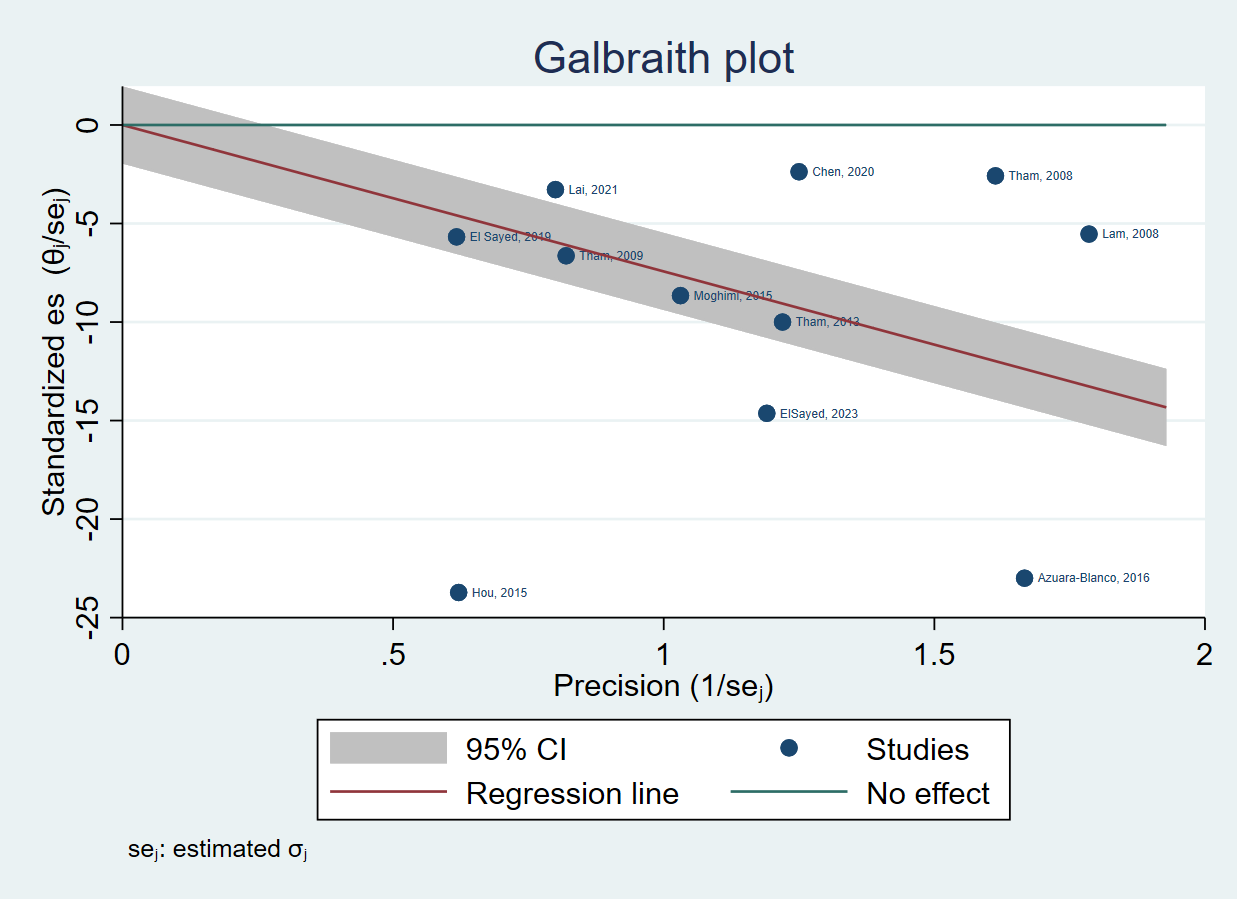


**Fig. S9.** Galbraith plot of changes in IOP at the 6-month follow-up in patients with closed-angle glaucoma


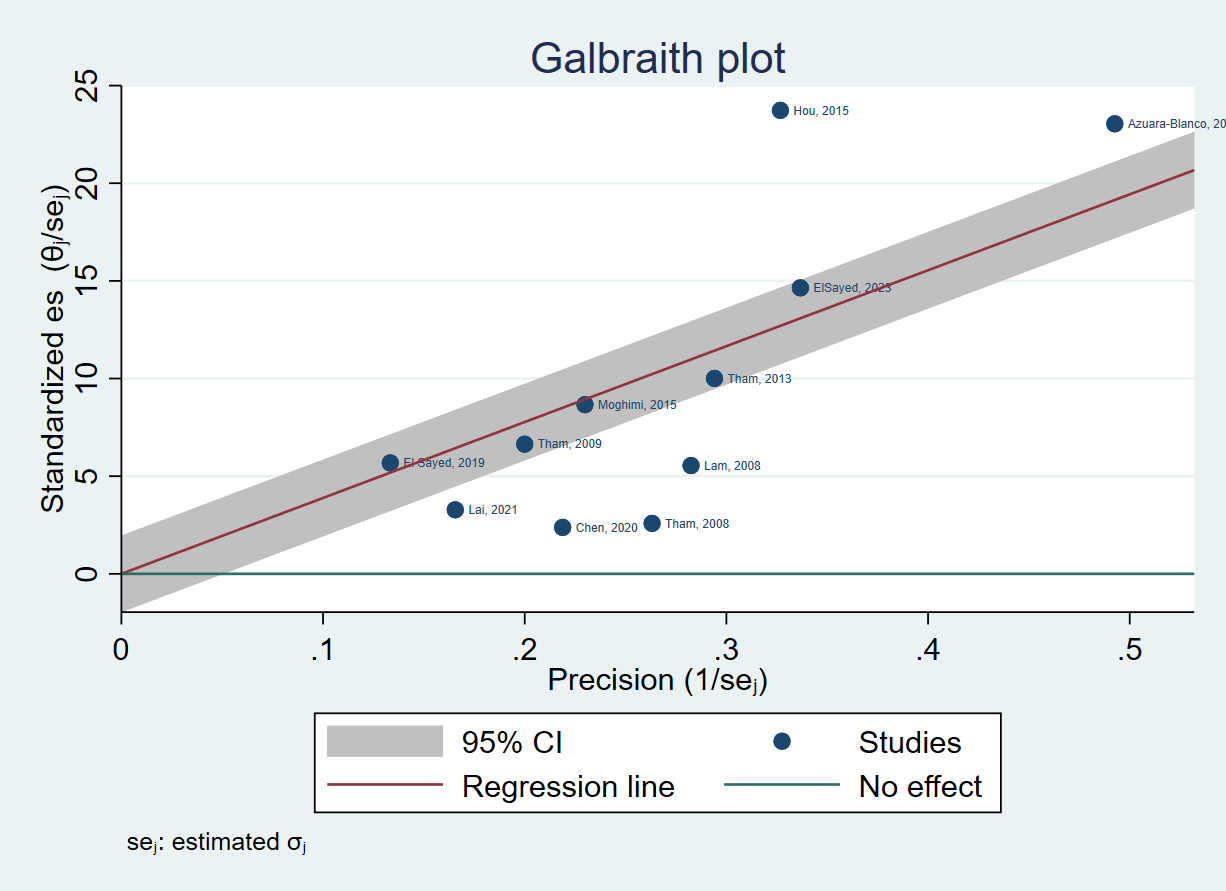


**Fig. S10.** Galbraith plot of the percentage of IOP reductions at the 6-month follow-up in patients with closed-angle glaucoma


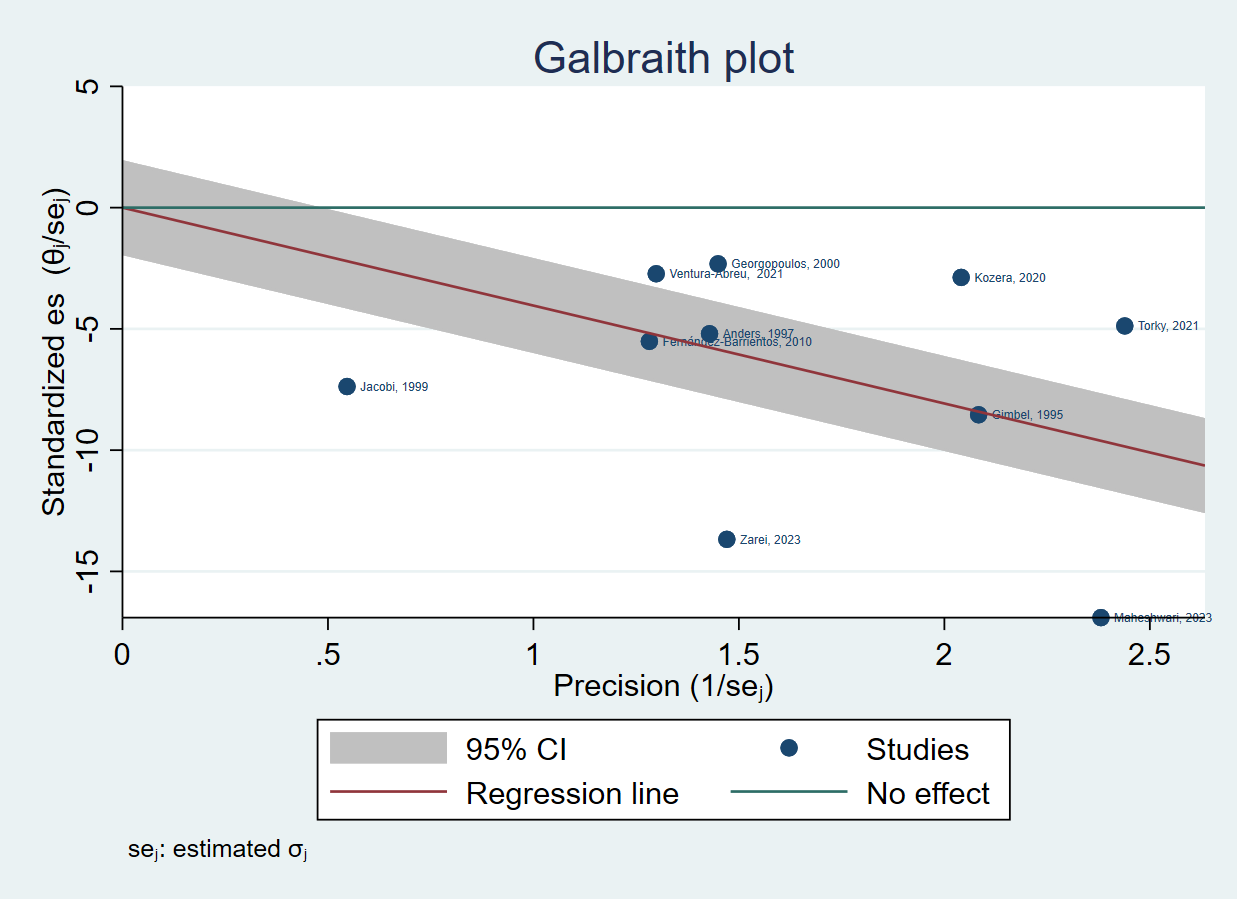


**Fig. S11.** Galbraith plot of changes in IOP at the 6-month follow-up in patients with open-angle glaucoma

**
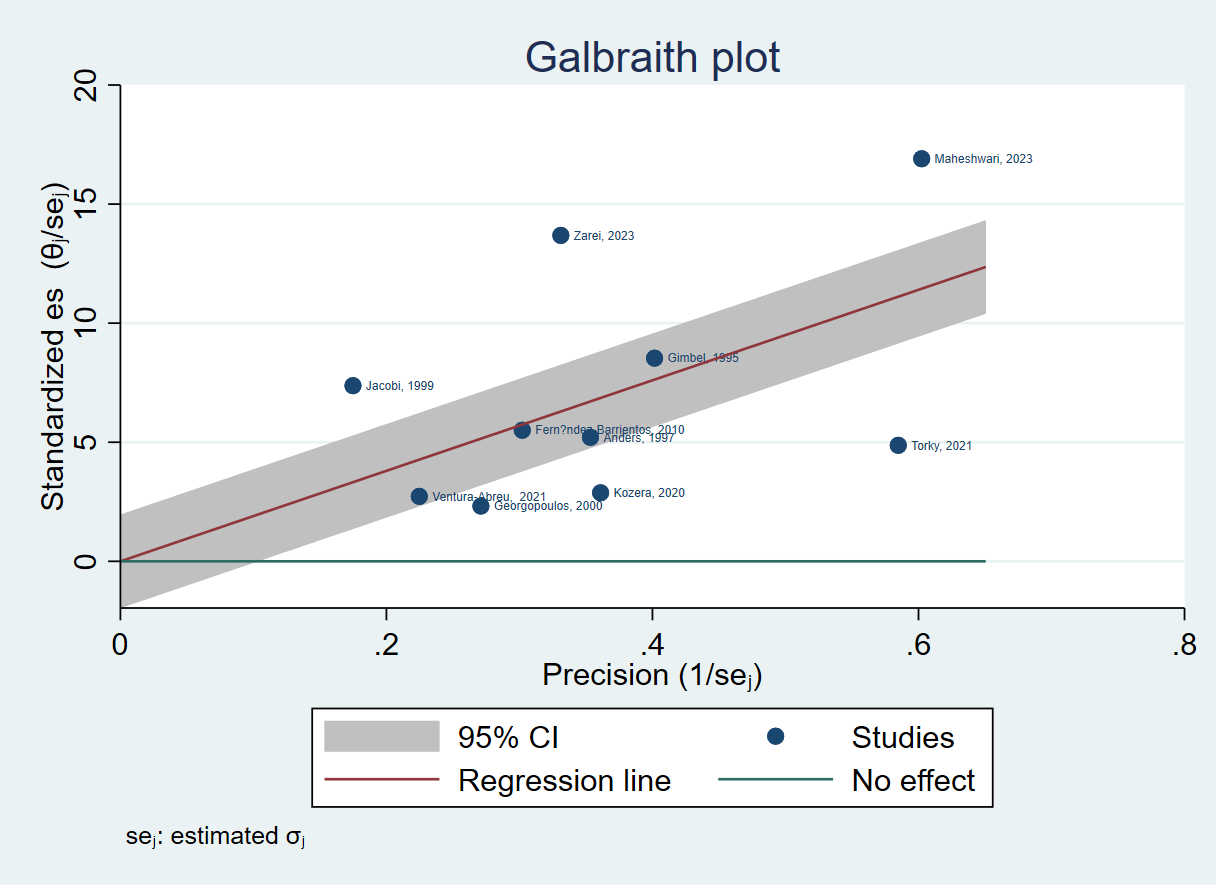
**

**Fig. S12.** Galbraith plot of the percentage of IOP reductions at the 6-month follow-up in patients with open-angle glaucoma


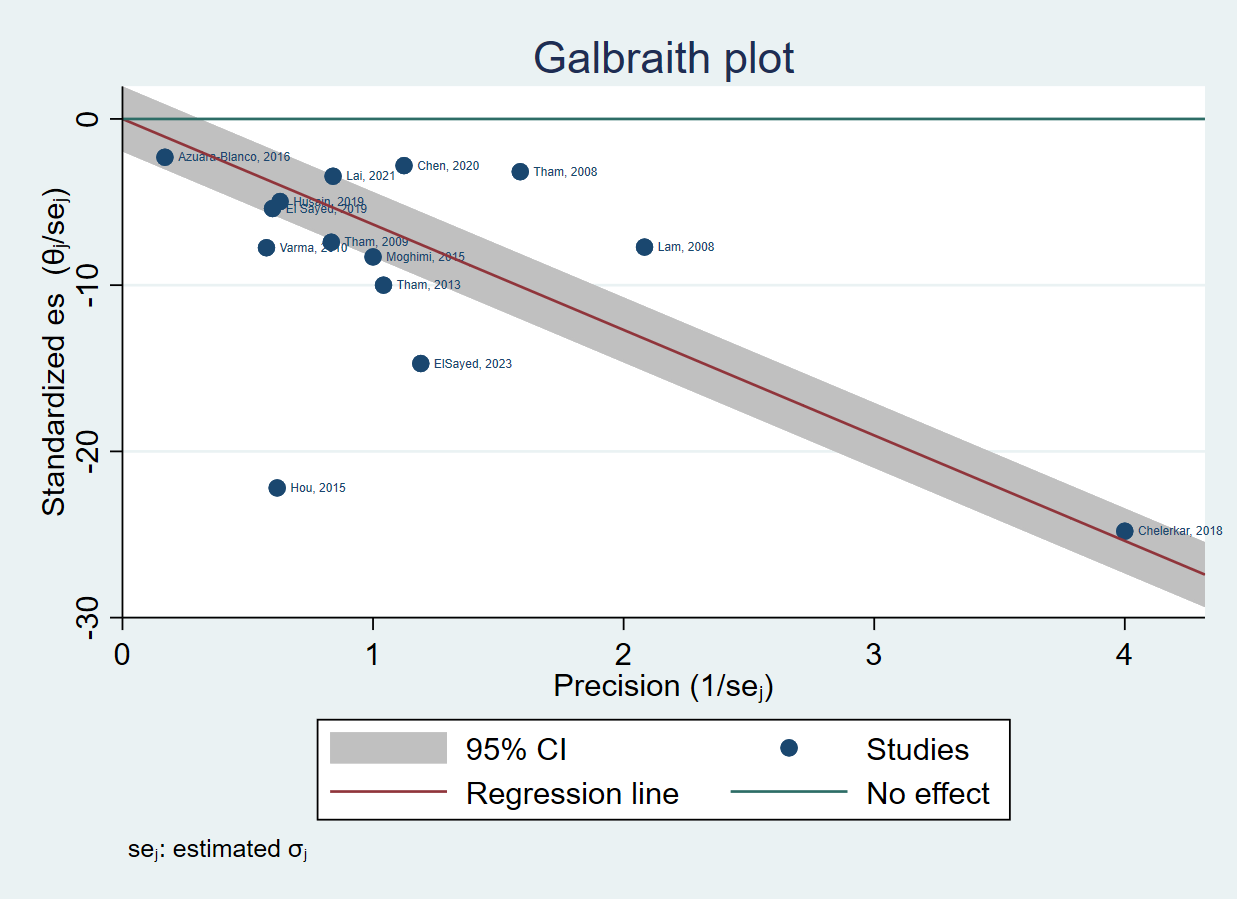


**Fig. S13.** Galbraith plot of changes in IOP at the 12-month follow-up in patients with closed-angle glaucoma


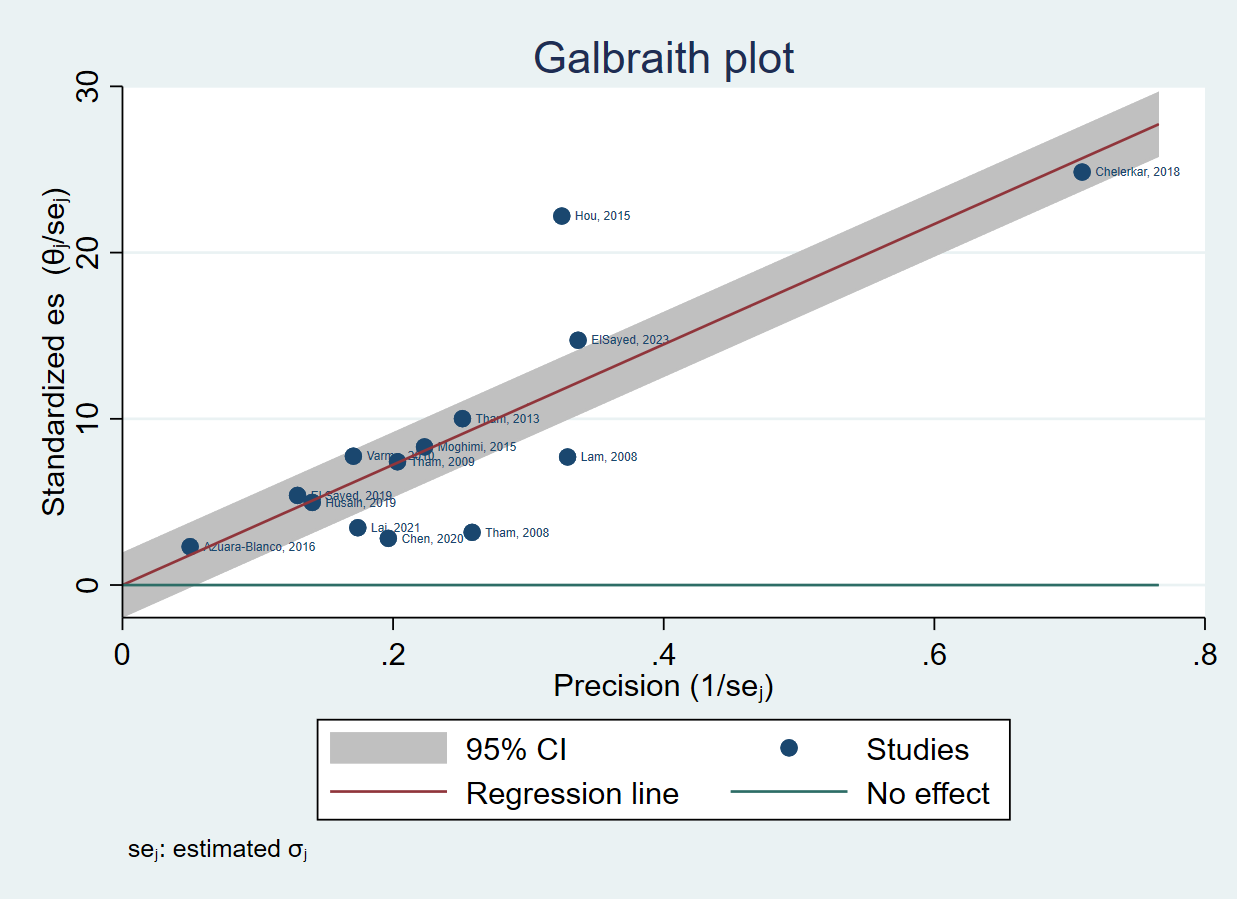


**Fig. S14.** Galbraith plot of the percentage of IOP reductions at the 12-month follow-up in patients with closed-angle glaucoma


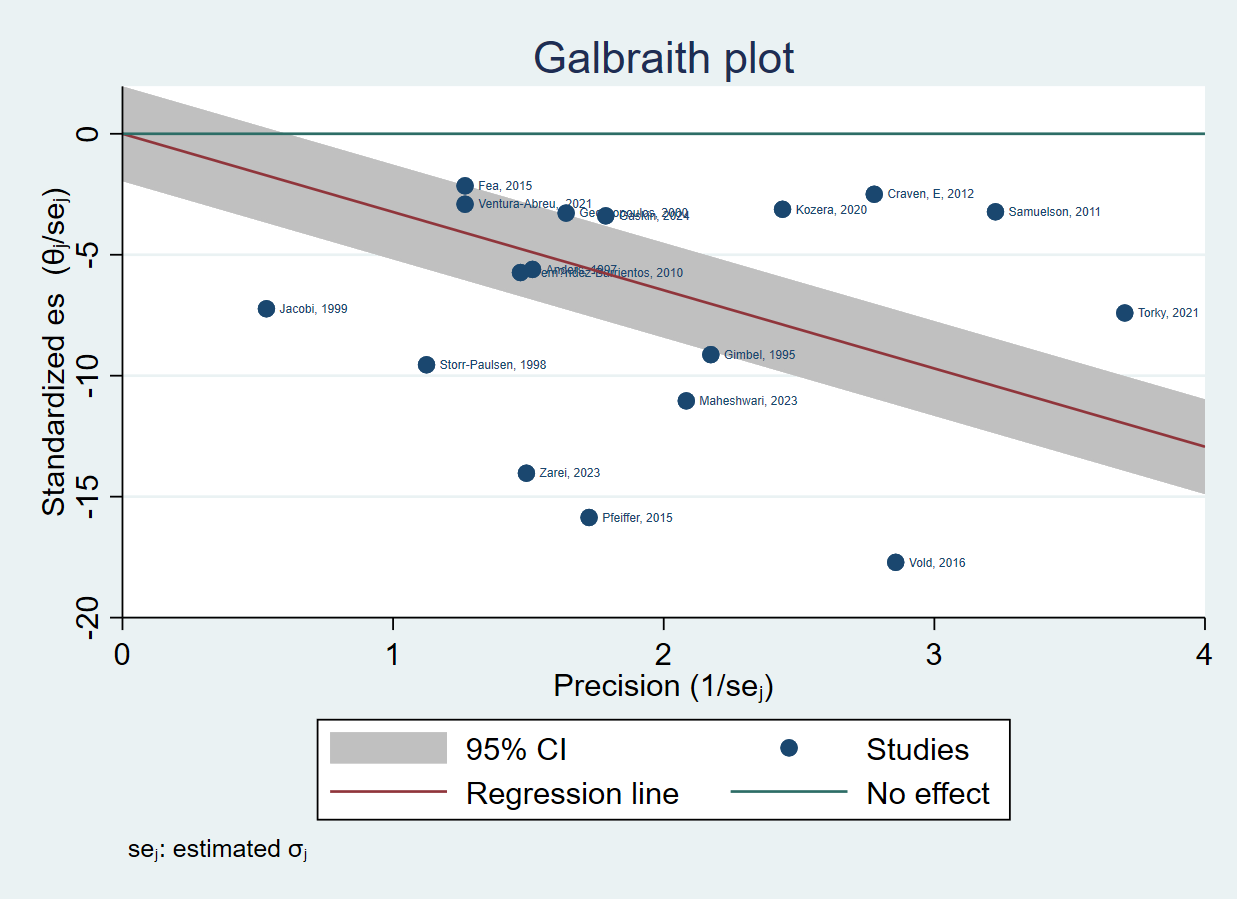


**Fig. S15.** Galbraith plot of changes in IOP at the 12-month follow-up in patients with open-angle glaucoma


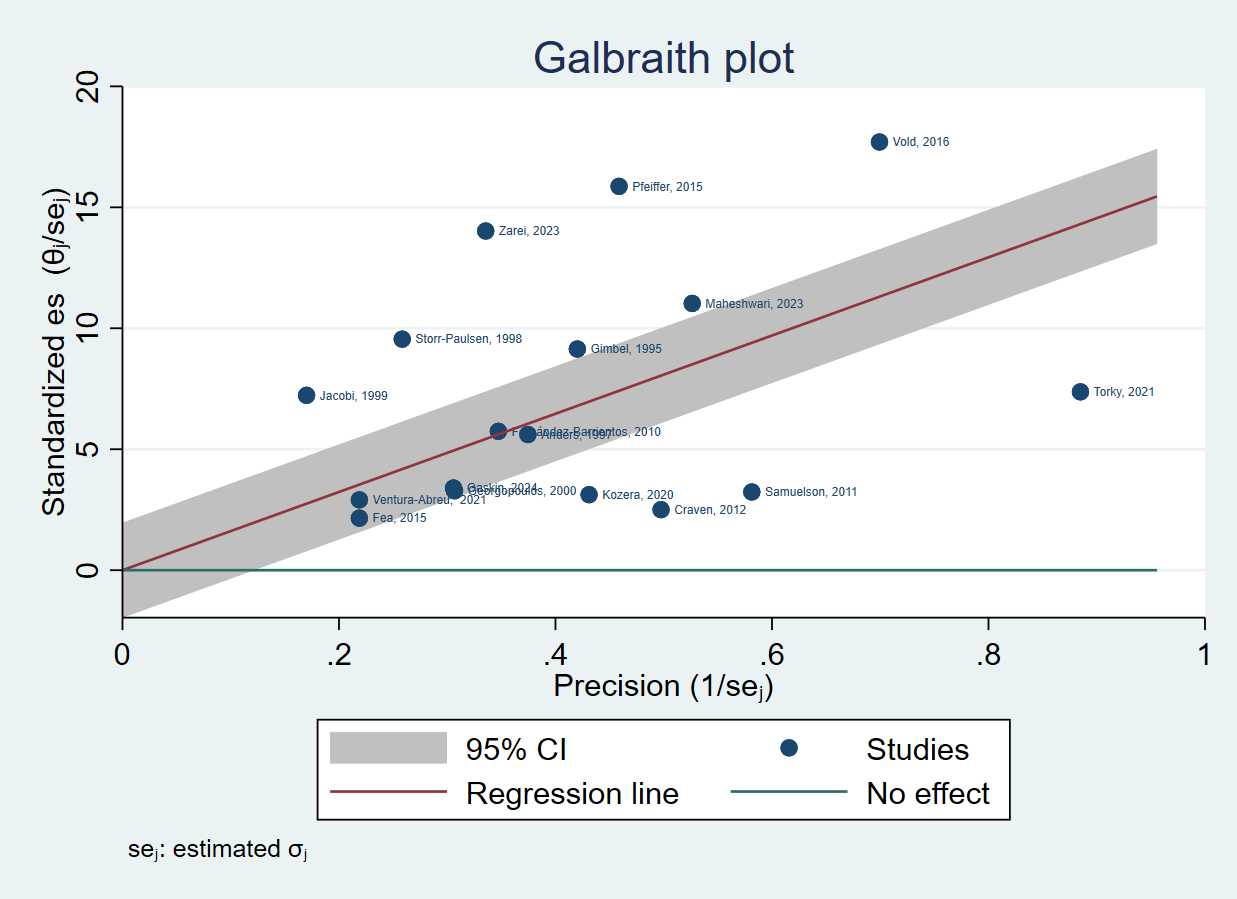


**Fig. S16.** Galbraith plot of the percentage of IOP reductions at the 12-month follow-up in patients with open-angle glaucoma

**
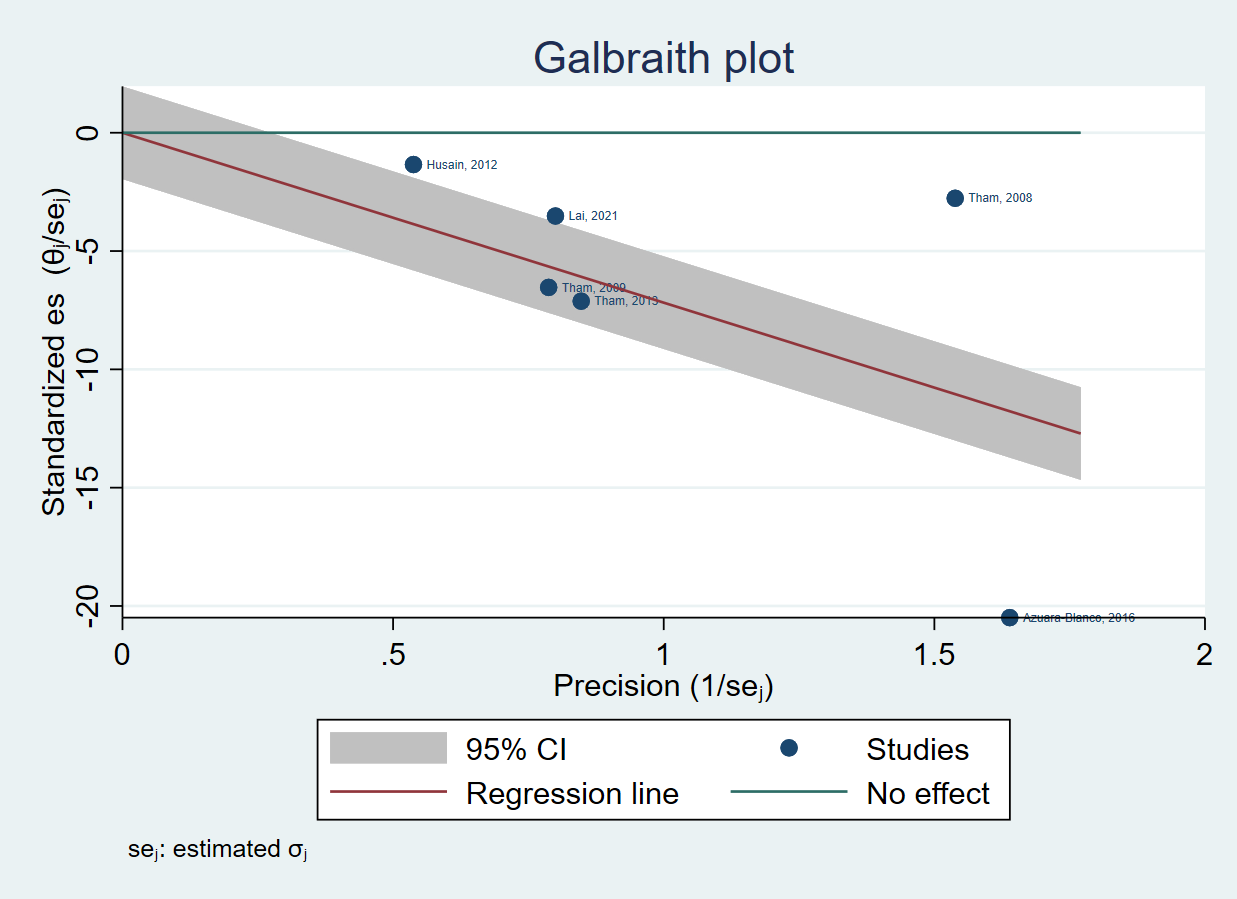
**

**Fig. S17.** Galbraith plot of changes in IOP at the 24-month follow-up in patients with closed-angle glaucoma


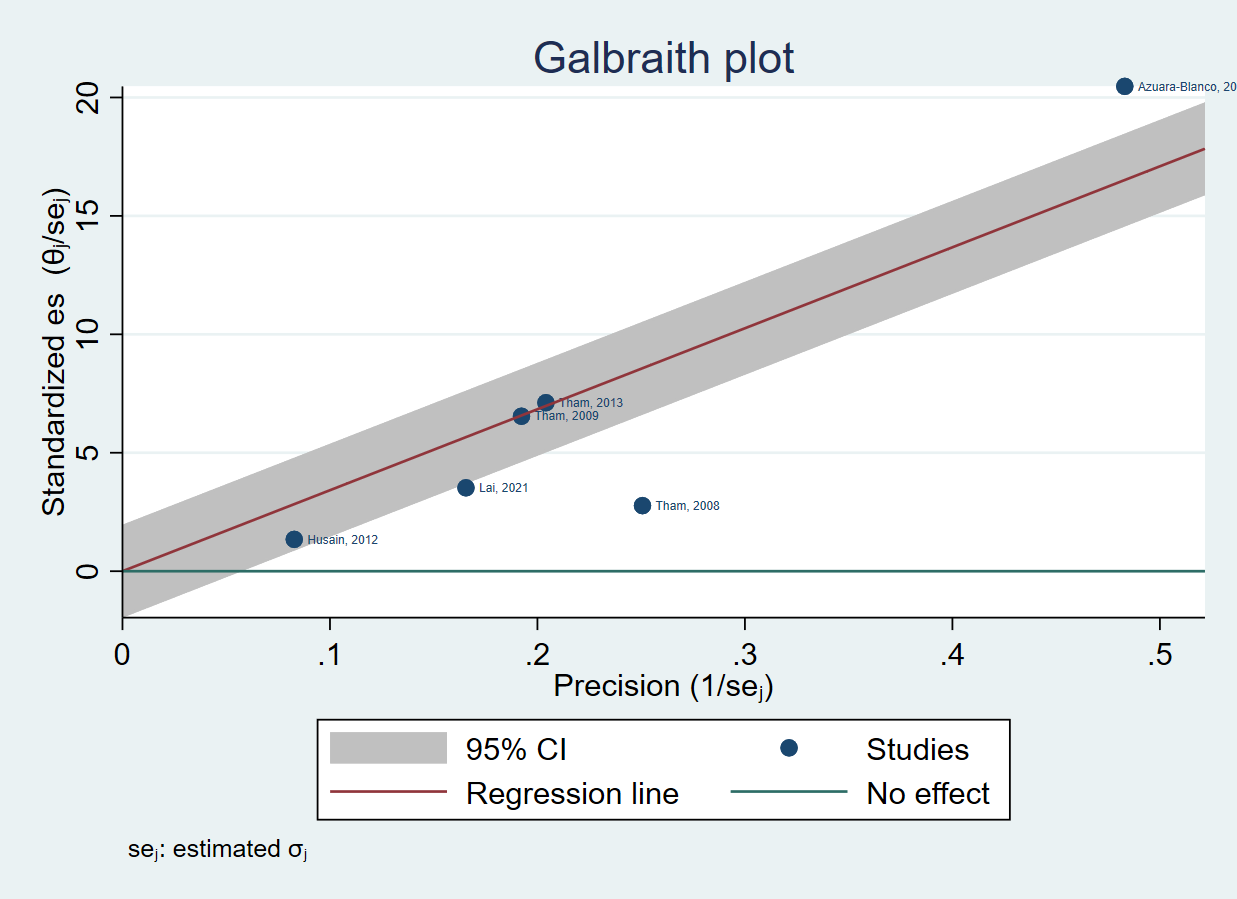


**Fig. S18.** Galbraith plot of the percentage of IOP reductions at the 24-month follow-up in patients with closed-angle glaucoma


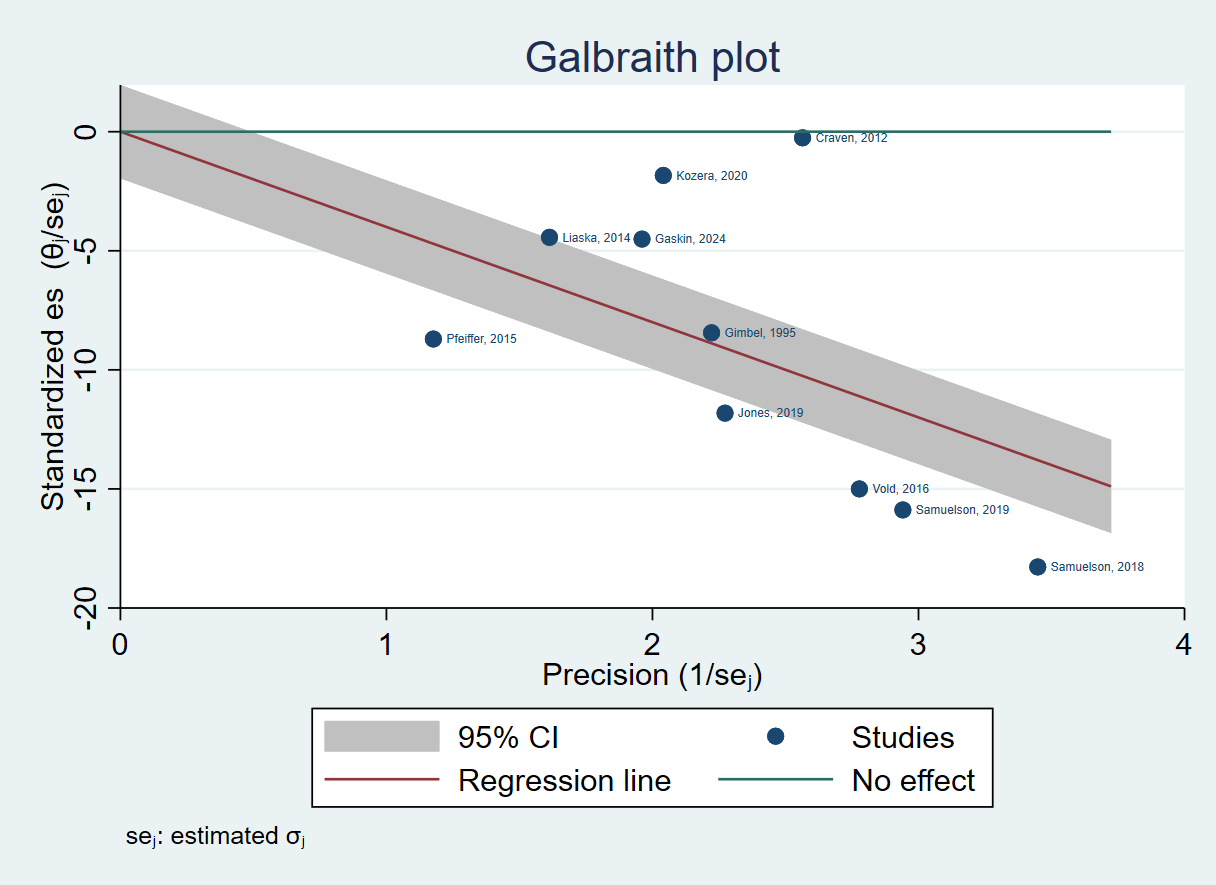


**Fig. S19.** Galbraith plot of changes in IOP at the 24-month follow-up in patients with open-angle glaucoma


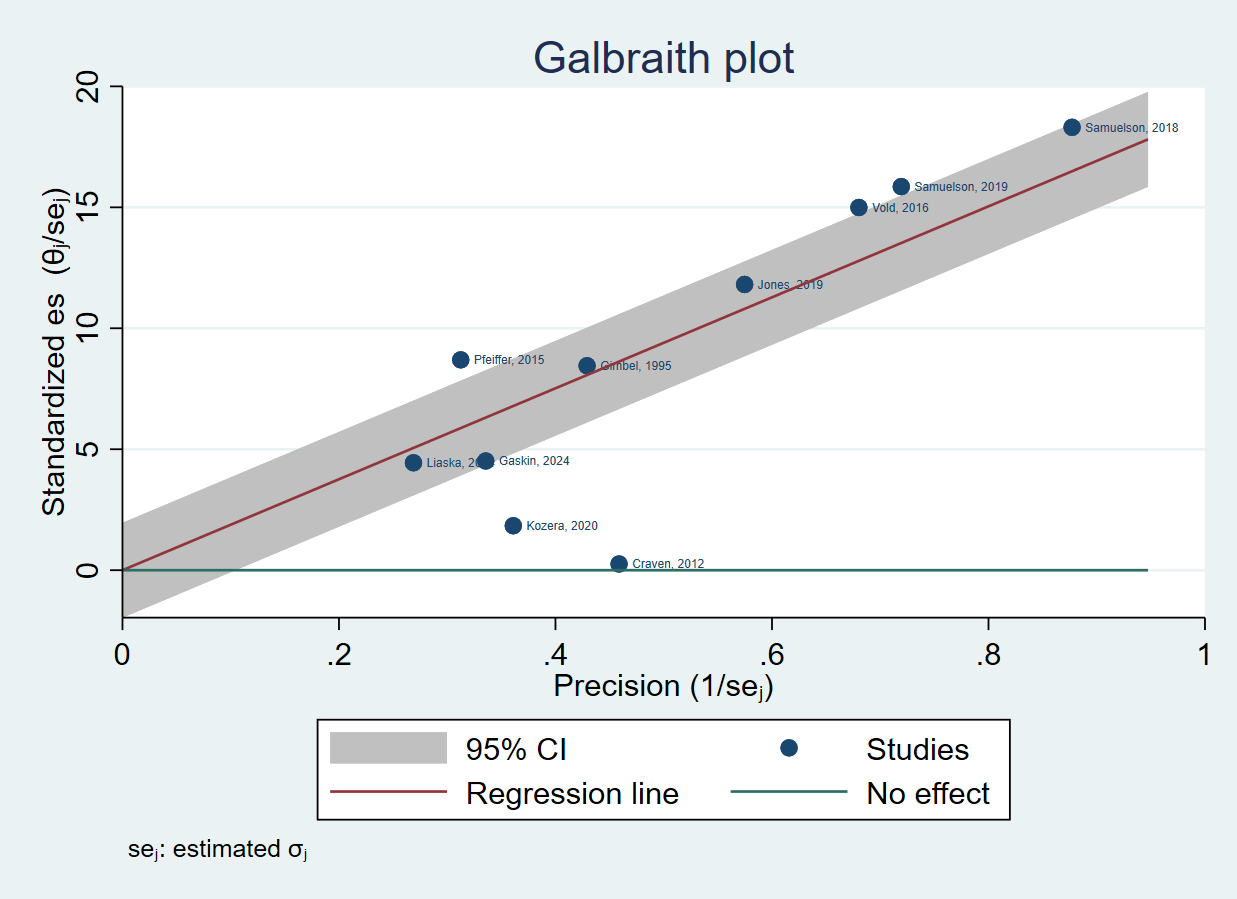


**Fig. S20.** Galbraith plot of the percentage of IOP reductions at the 24-month follow-up in patients with open-angle glaucoma


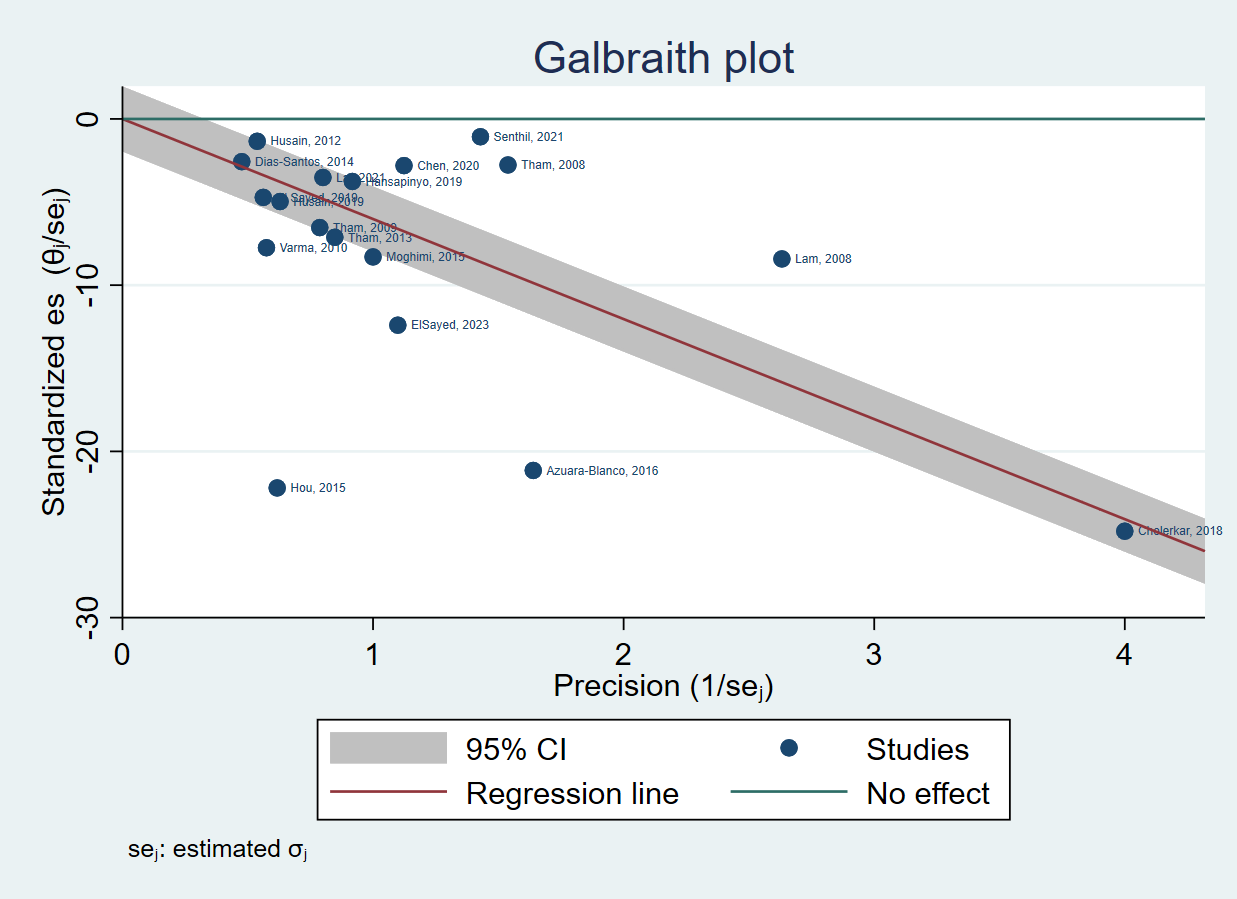


**Fig. S21.** Galbraith plot of changes in IOP at the last follow-up in patients with closed-angle glaucoma


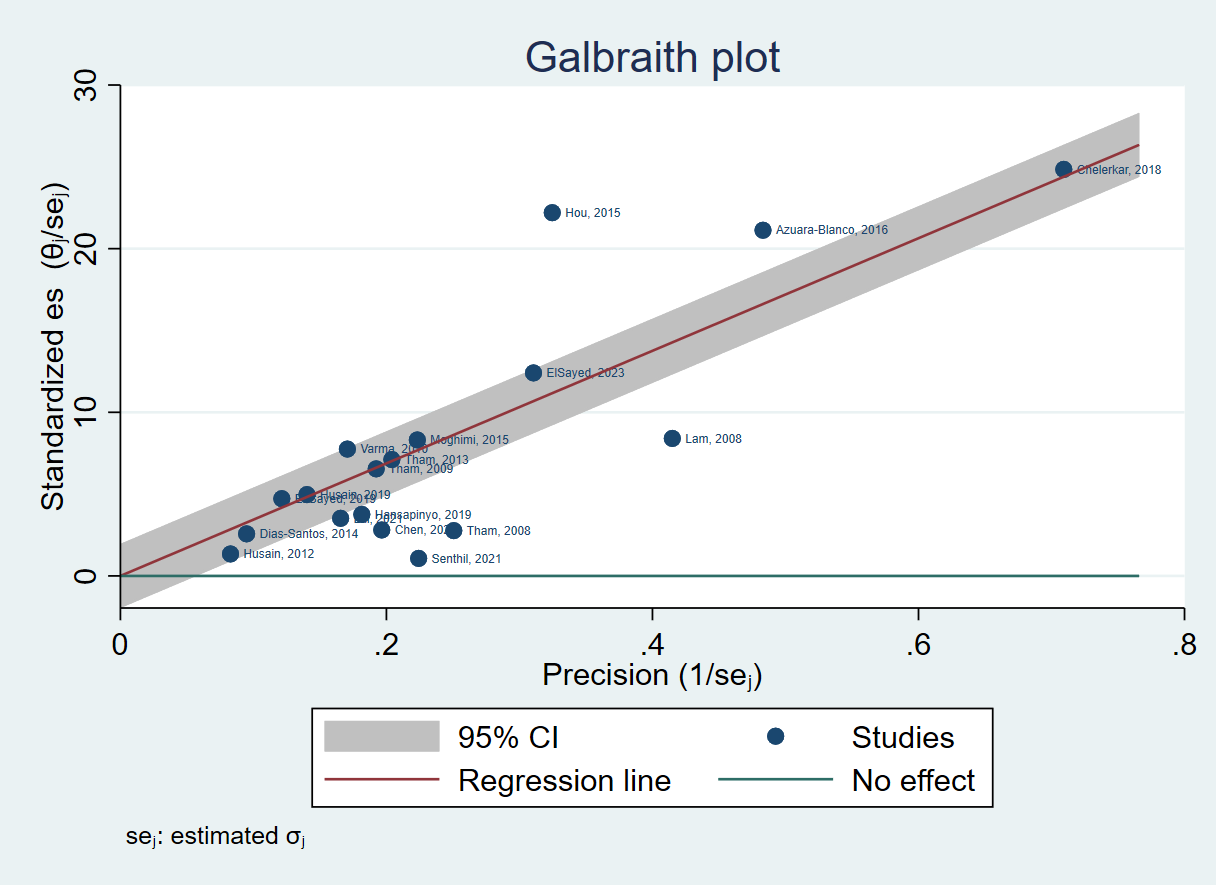


**Fig. S22.** Galbraith plot of the percentage of IOP reductions at the last follow-up in patients with closed-angle glaucoma


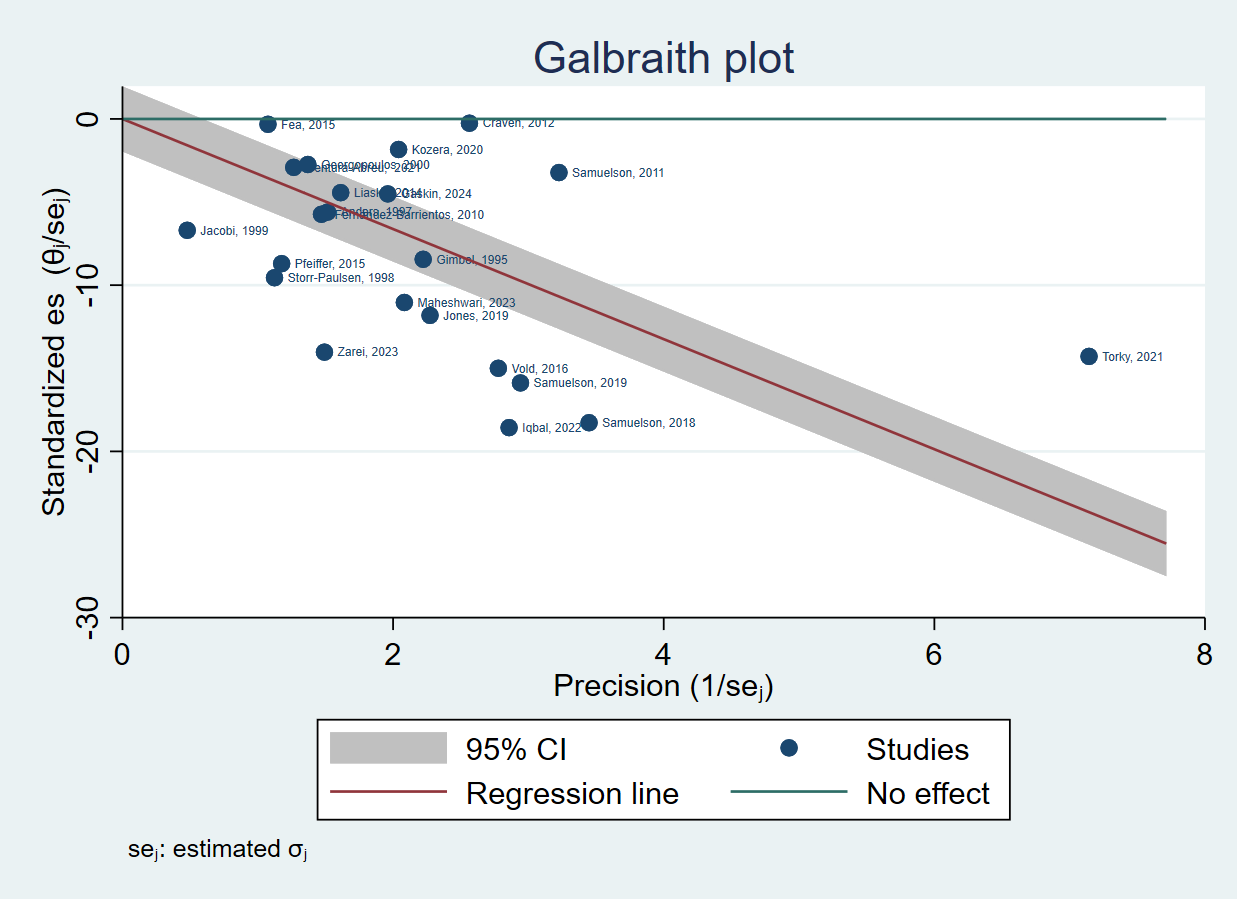


**Fig. S23.** Galbraith plot of changes in IOP at the last follow-up in patients with open-angle glaucoma

**
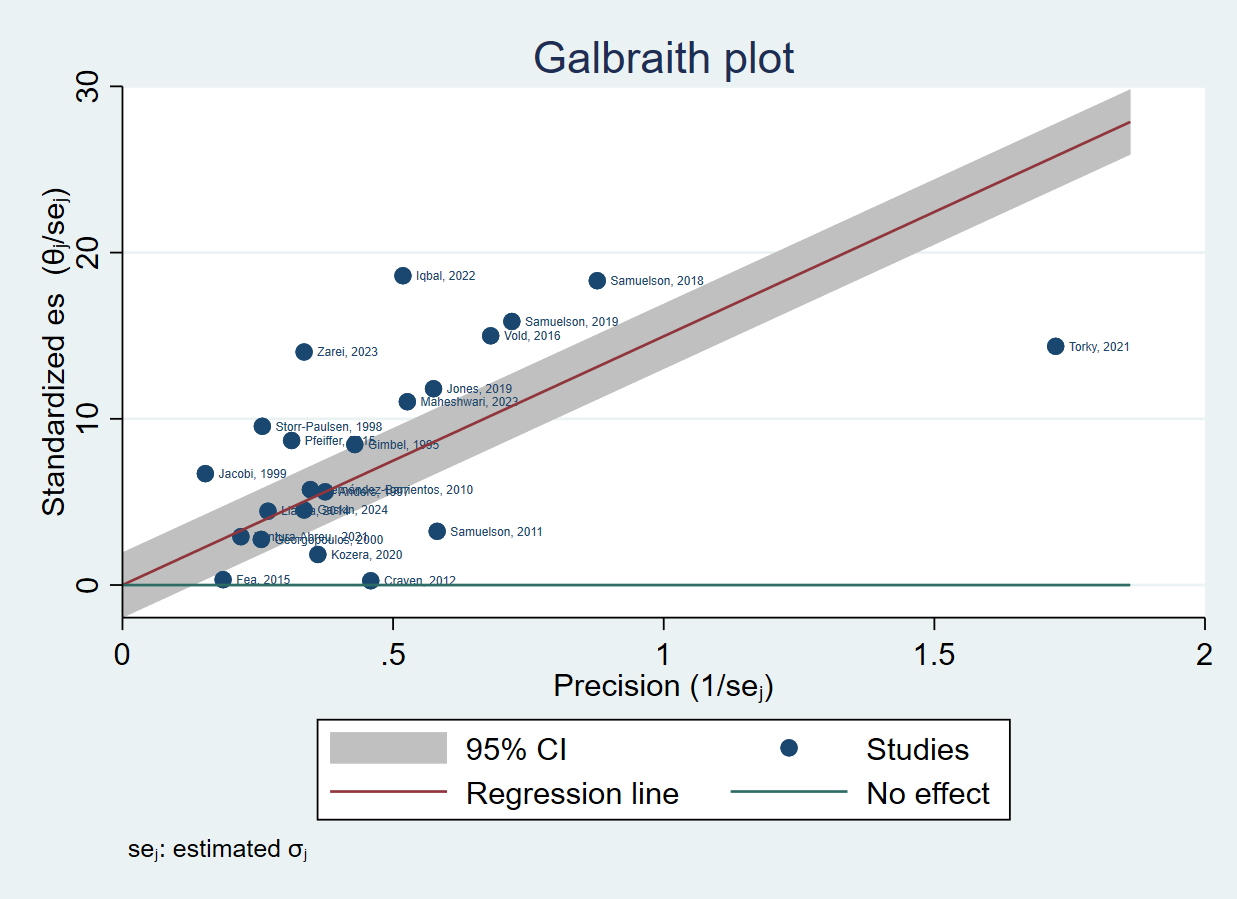
**

**Fig. S24.** Galbraith plot of the percentage of IOP reductions at the last follow-up in patients with open-angle glaucoma

**Sensitivity analysis:**


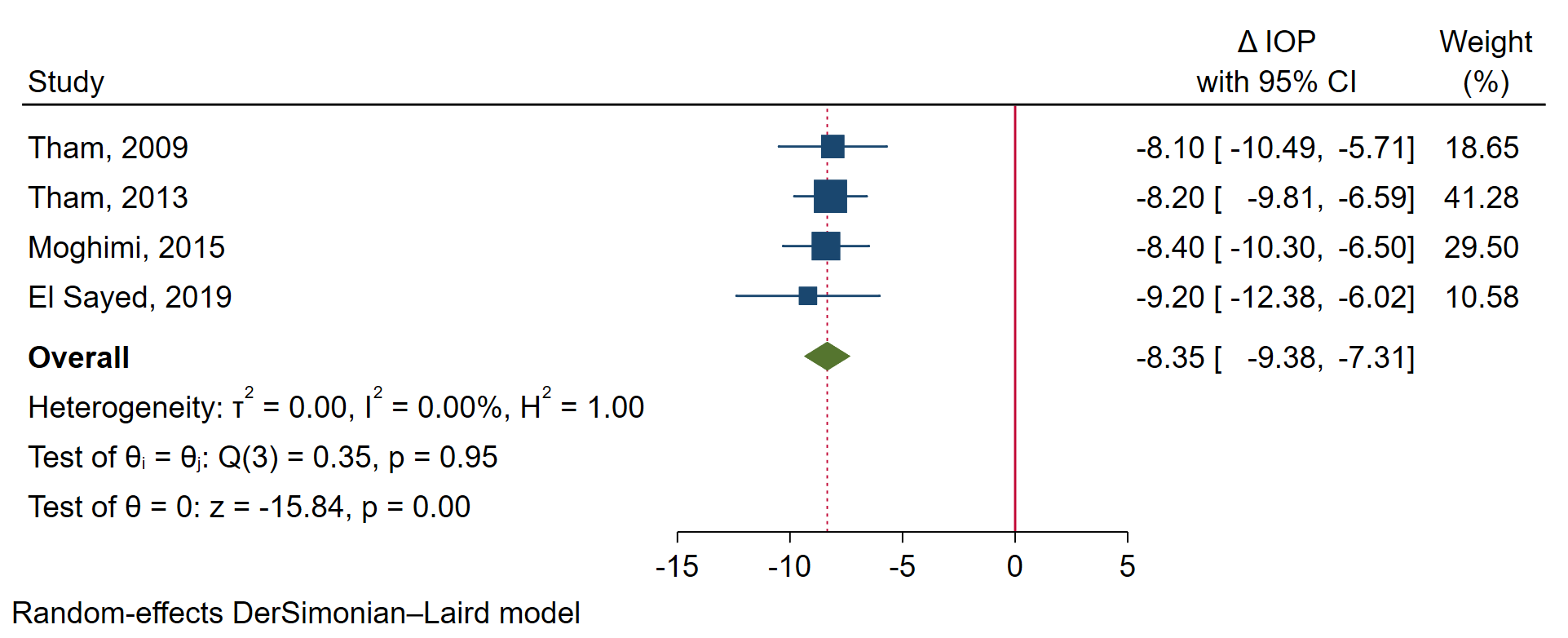


**Fig. S25.** Forest plot of 6-month IOP change in closed-angle glaucoma after excluding outliers.


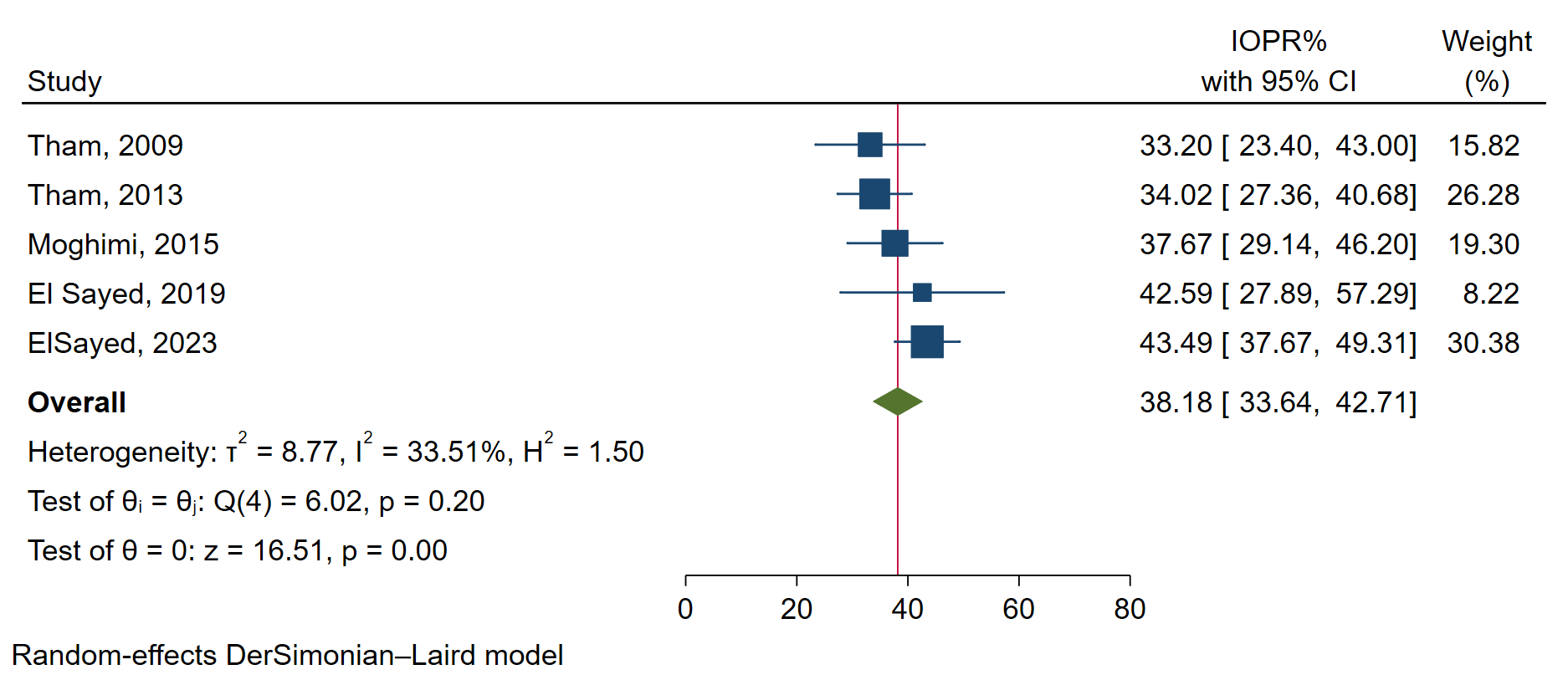


**Fig. S26.** Forest plot of 6-month IOPR% in closed-angle glaucoma after excluding outliers.


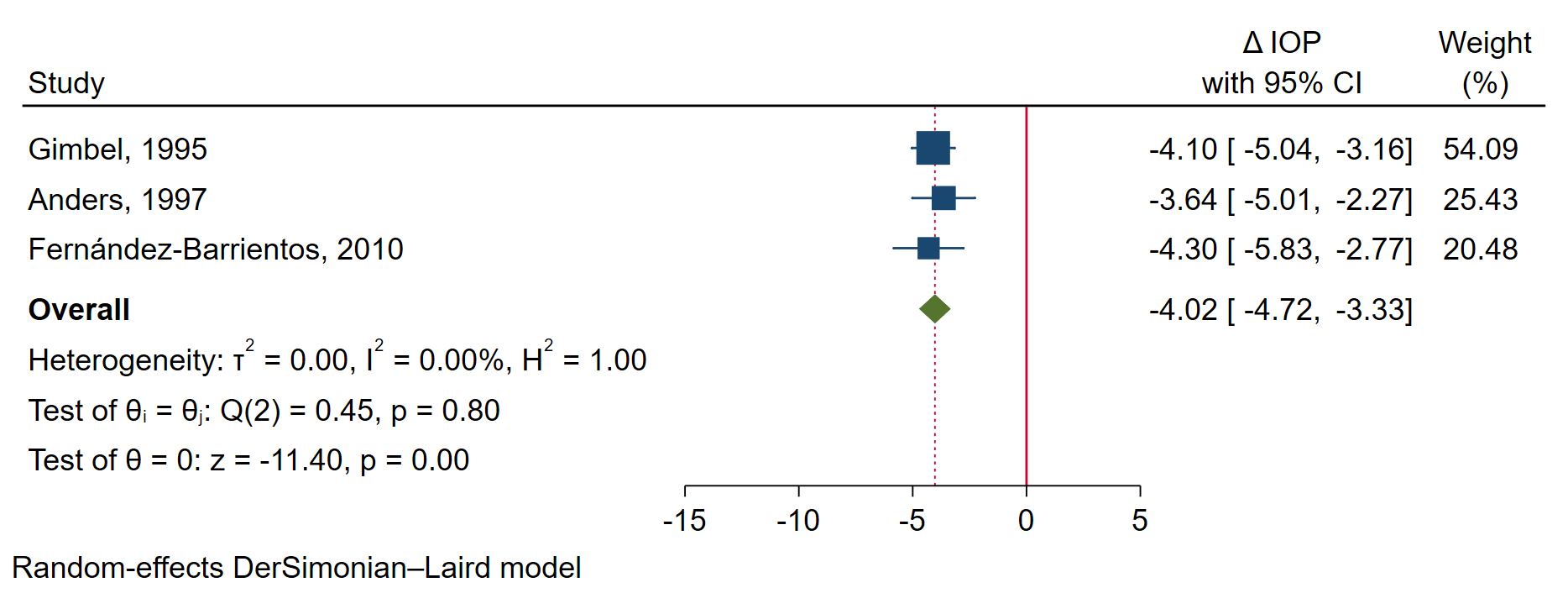


**Fig. S27.** Forest plot of 6-month IOP change in open-angle glaucoma after excluding outliers.


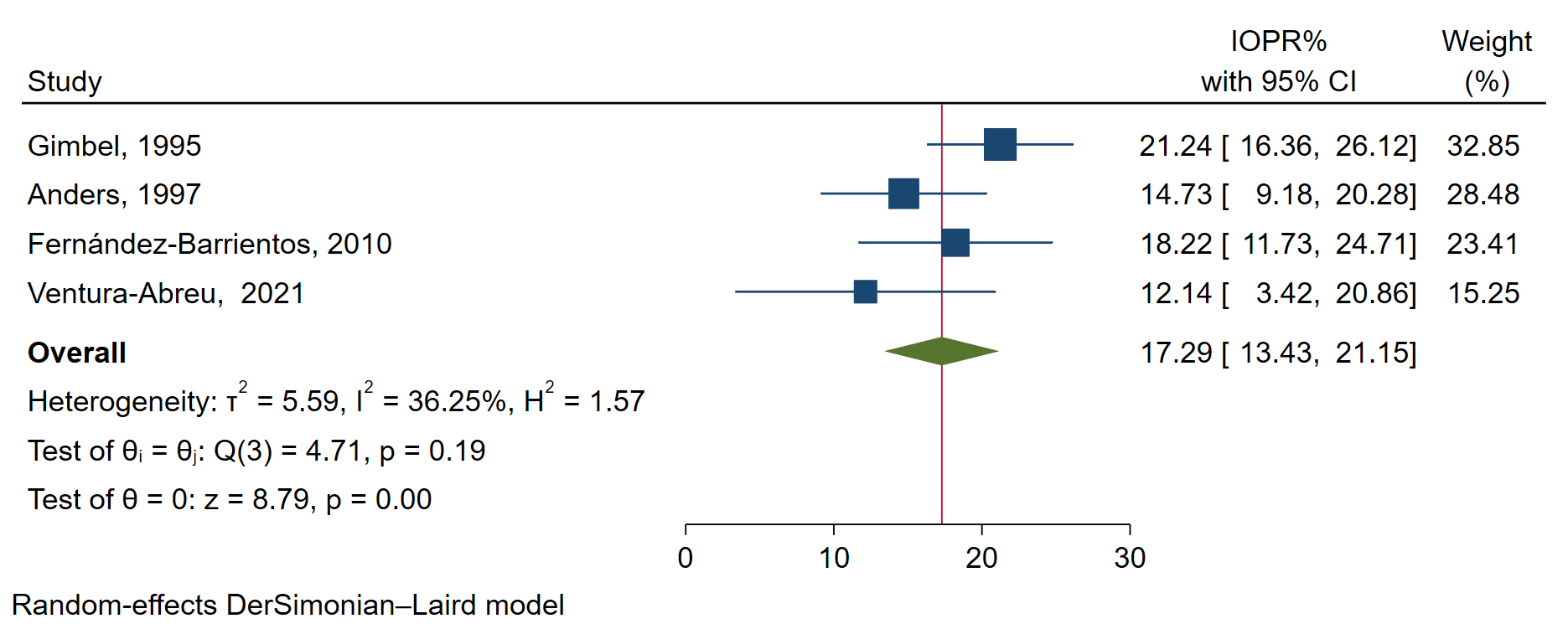


**Fig. S28.** Forest plot of 6-month IOPR% in open-angle glaucoma after excluding outliers.

**
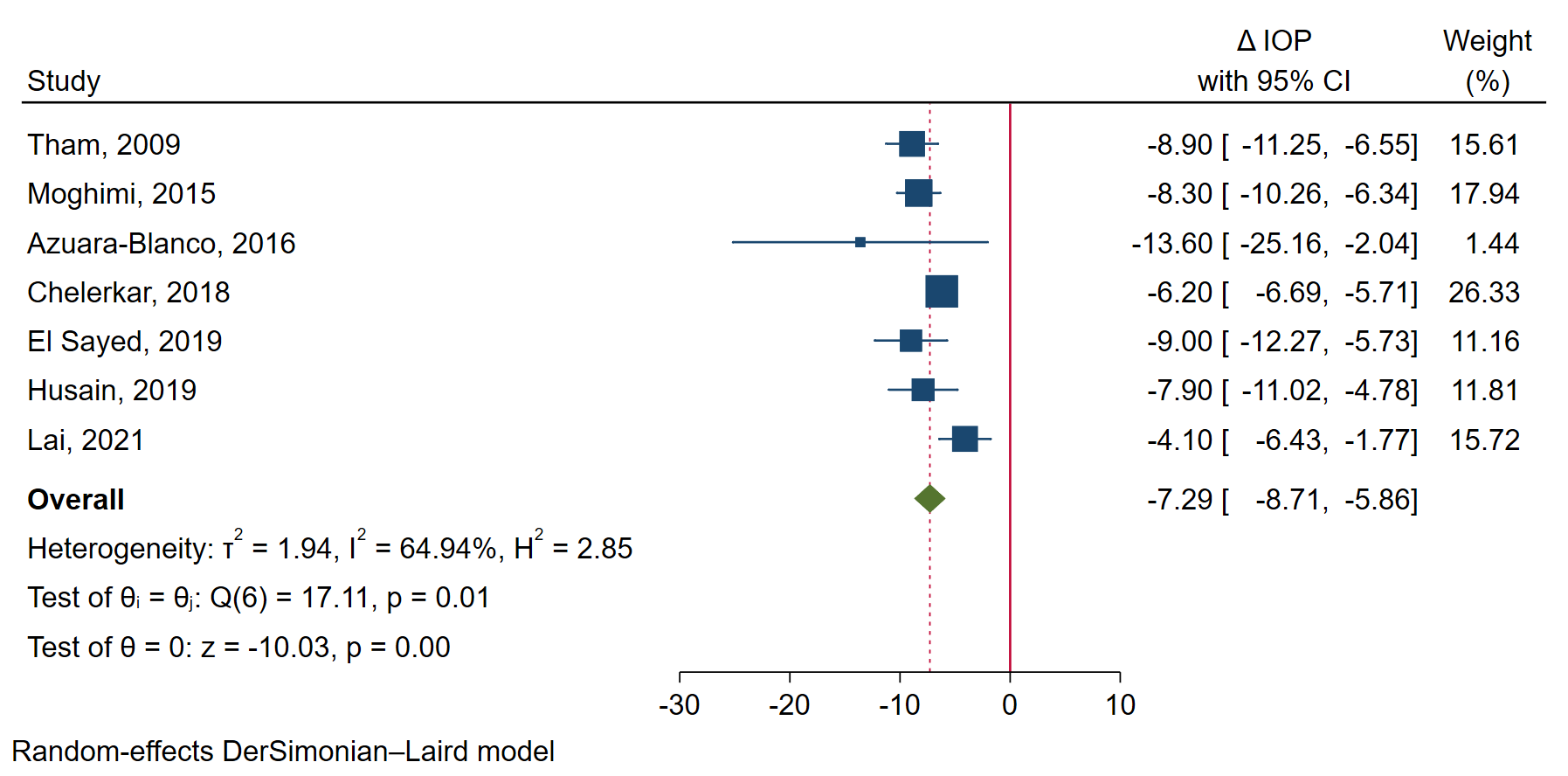
**

**Fig. S29.** Forest plot of 12-month IOP change in closed-angle glaucoma after excluding outliers.

*
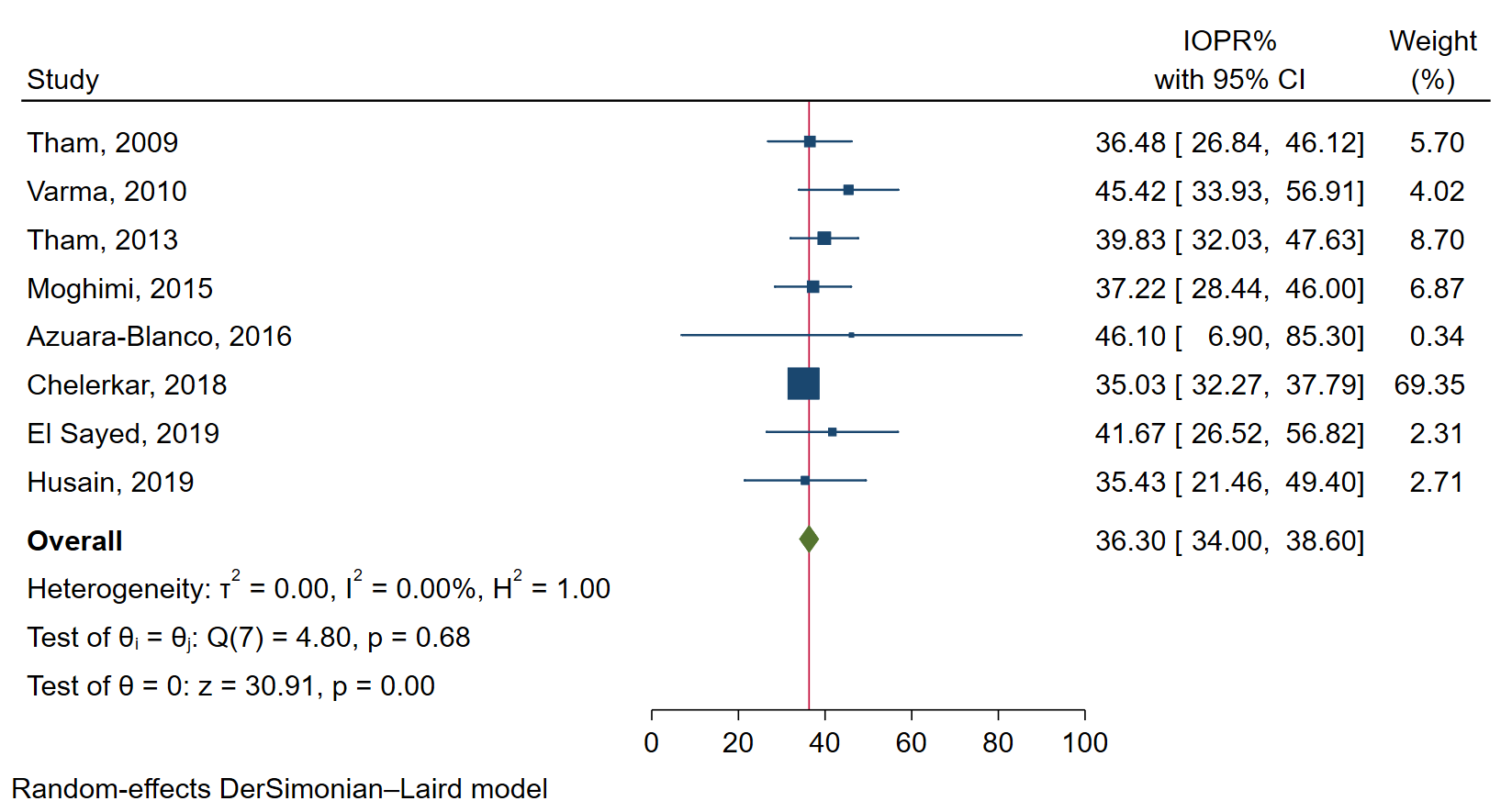
*

**Fig. S30.** Forest plot of 12-month IOPR% in closed-angle glaucoma after excluding outliers.

**
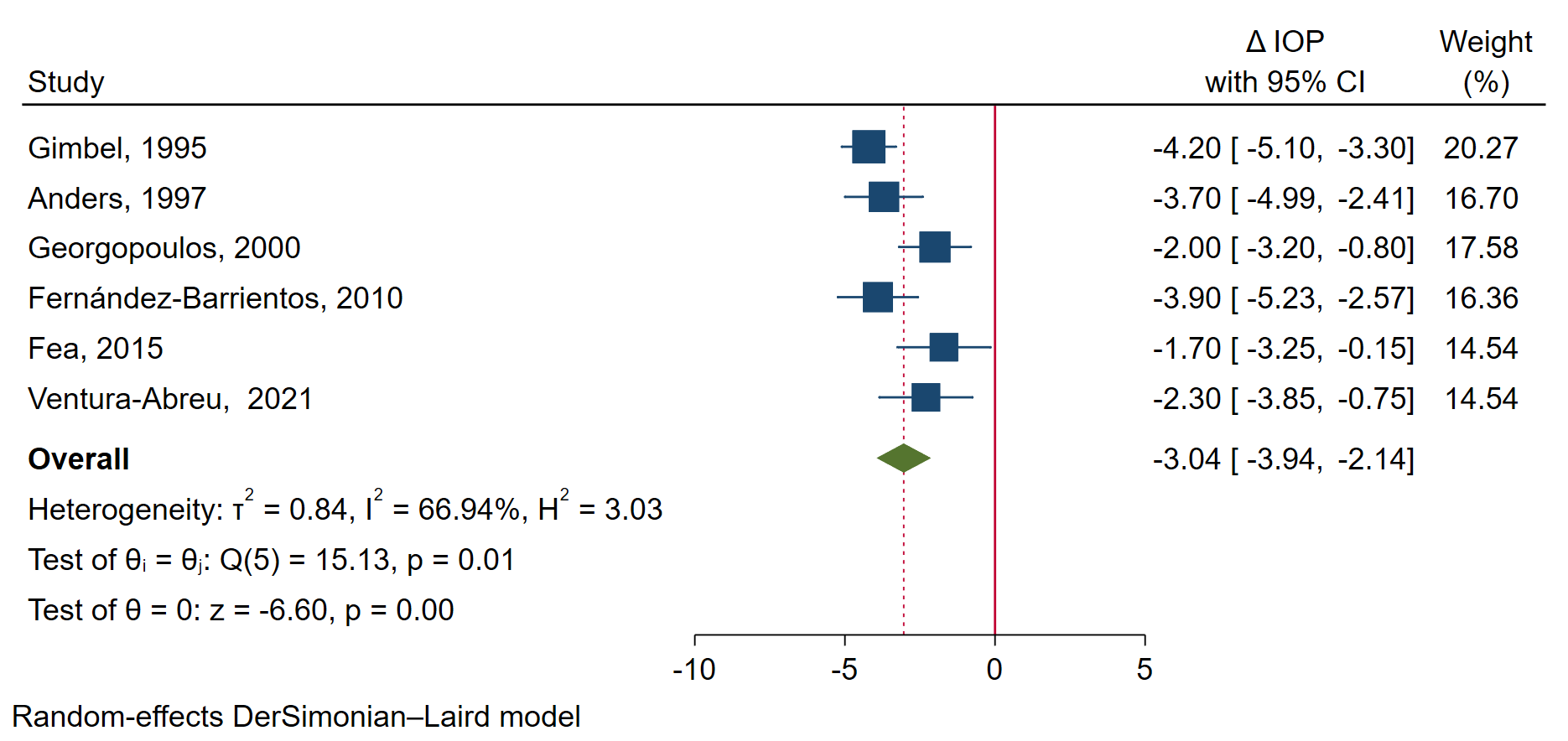
**

**Fig. S31.** Forest plot of 12-month IOP change in open-angle glaucoma after excluding outliers.


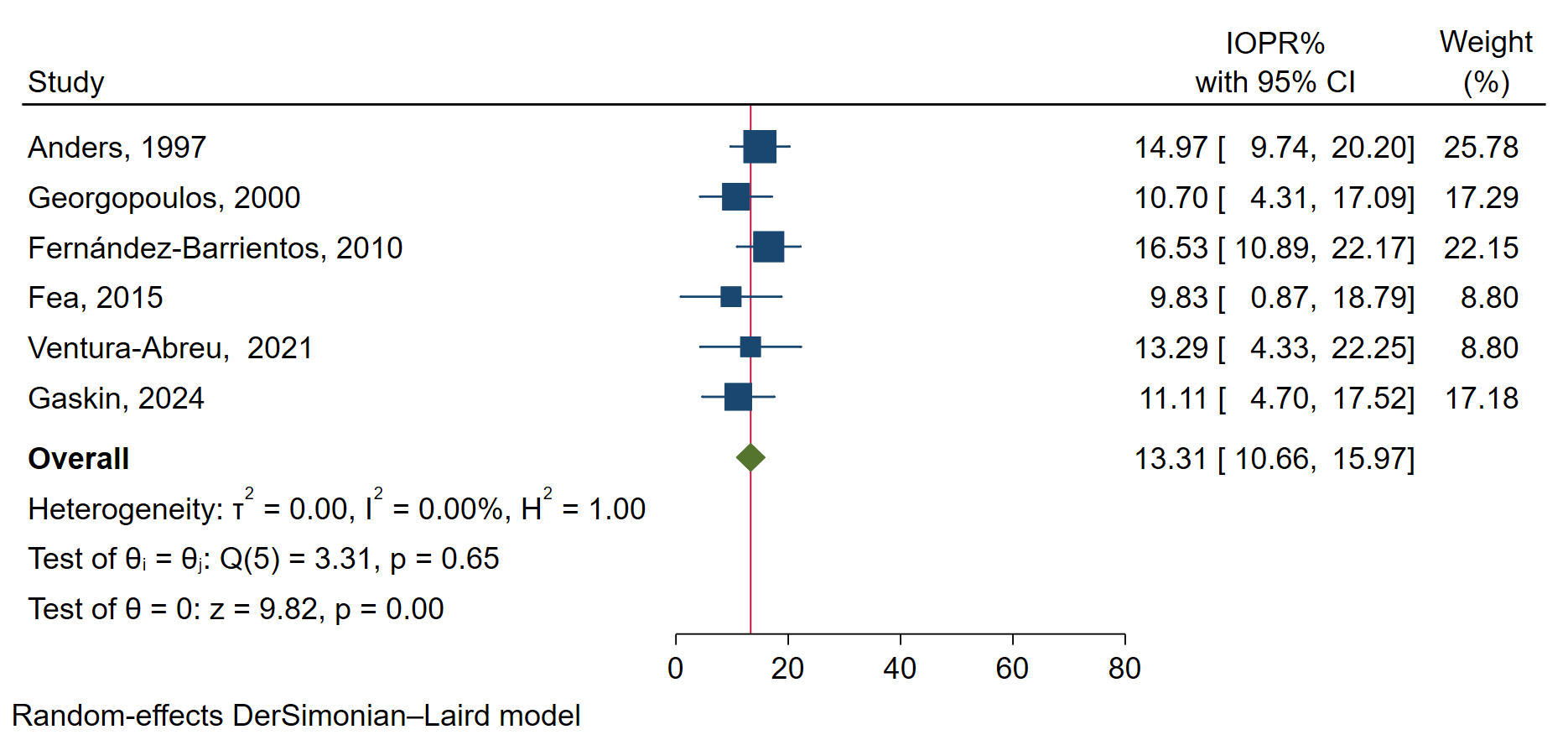


**Fig. S32.** Forest plot of 12-month IOPR% in open-angle glaucoma after excluding outliers.


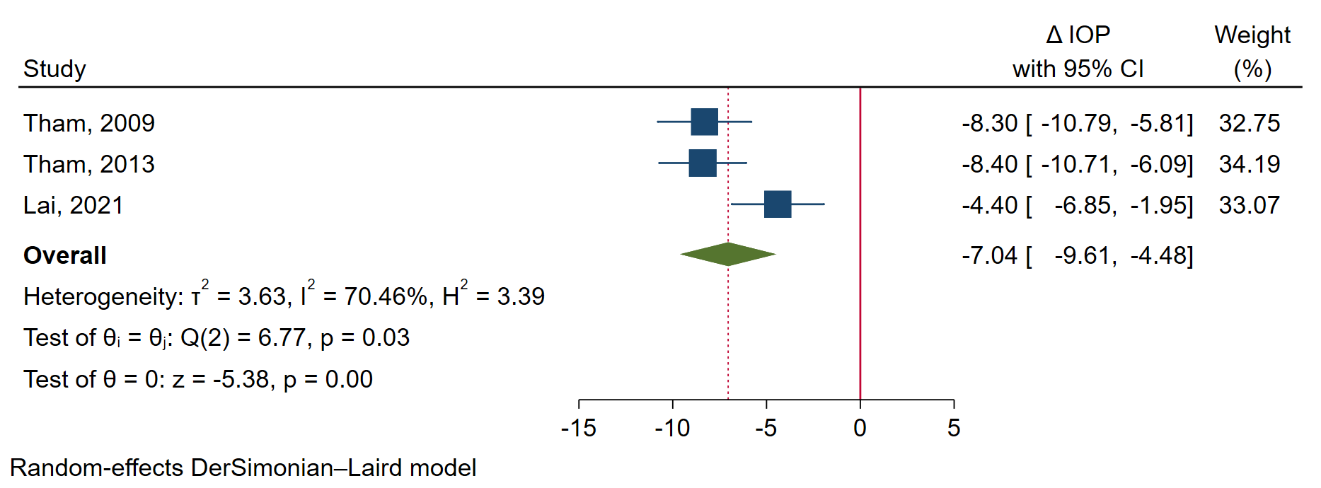


**Fig. S33.** Forest plot of 24-month IOP change in closed-angle glaucoma after excluding outliers.

*
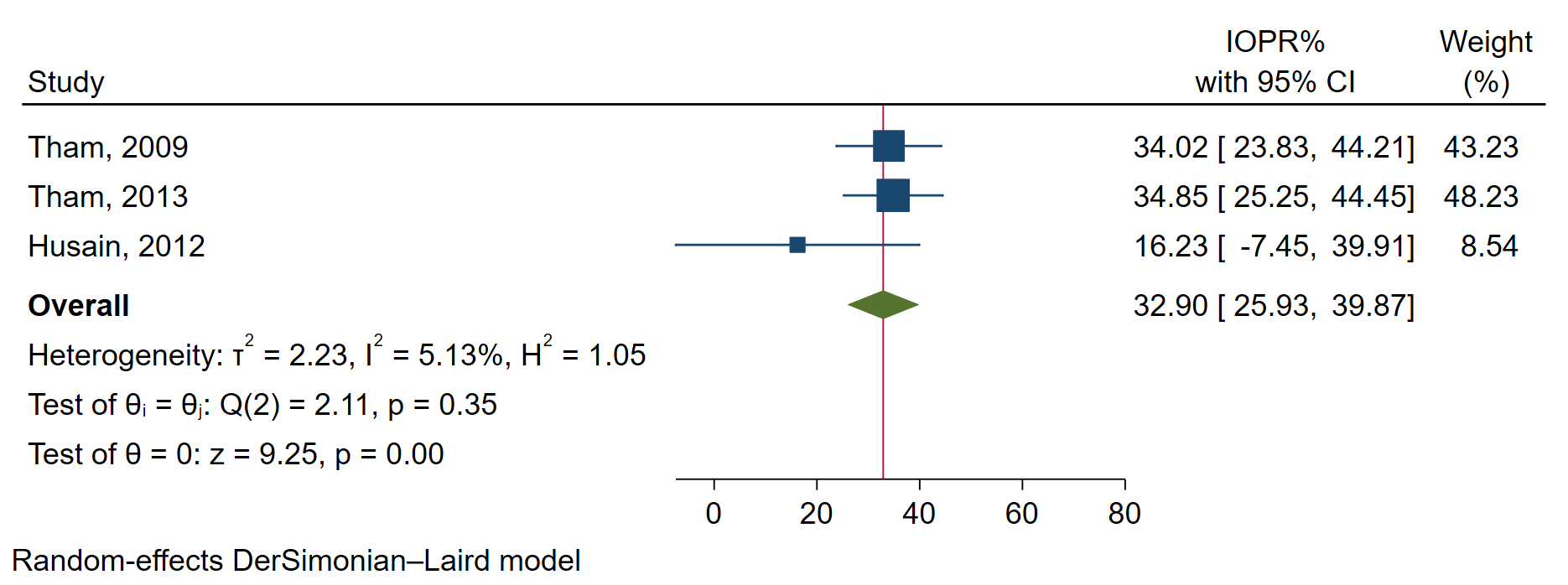
*

**Fig. S34.** Forest plot of 24-month IOPR% in closed-angle glaucoma after excluding outliers.

**
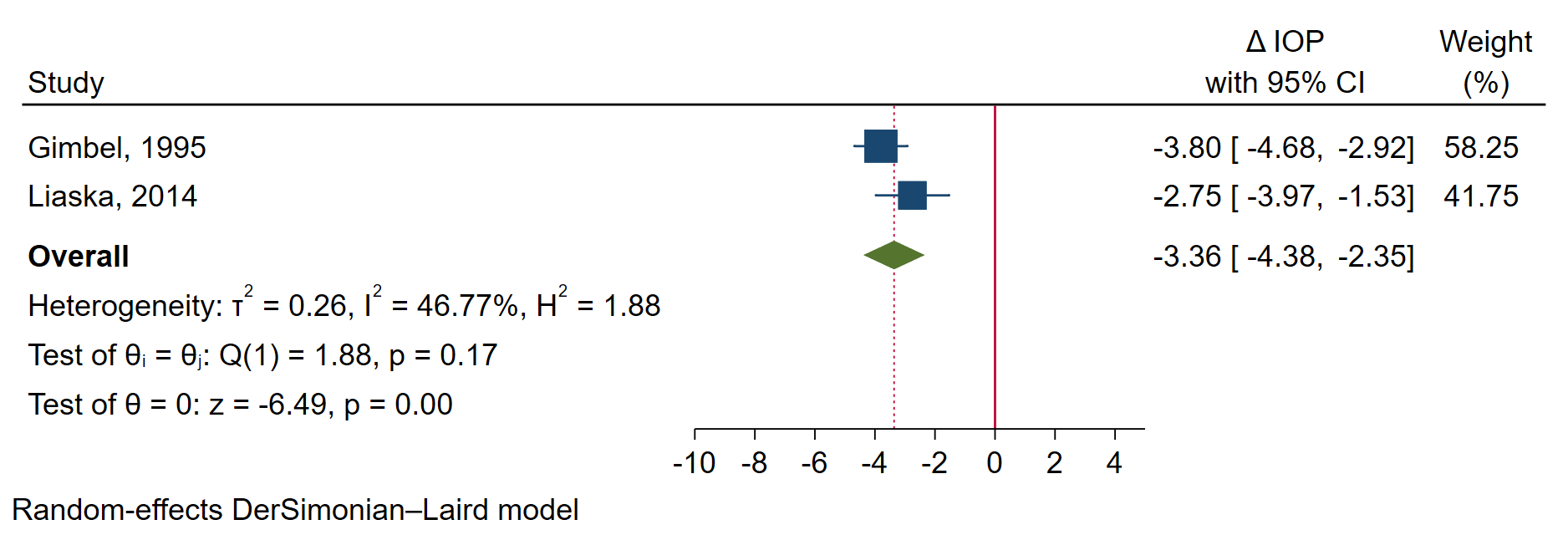
**

**Fig. S35.** Forest plot of 24-month IOP change in open-angle glaucoma after excluding outliers.

*
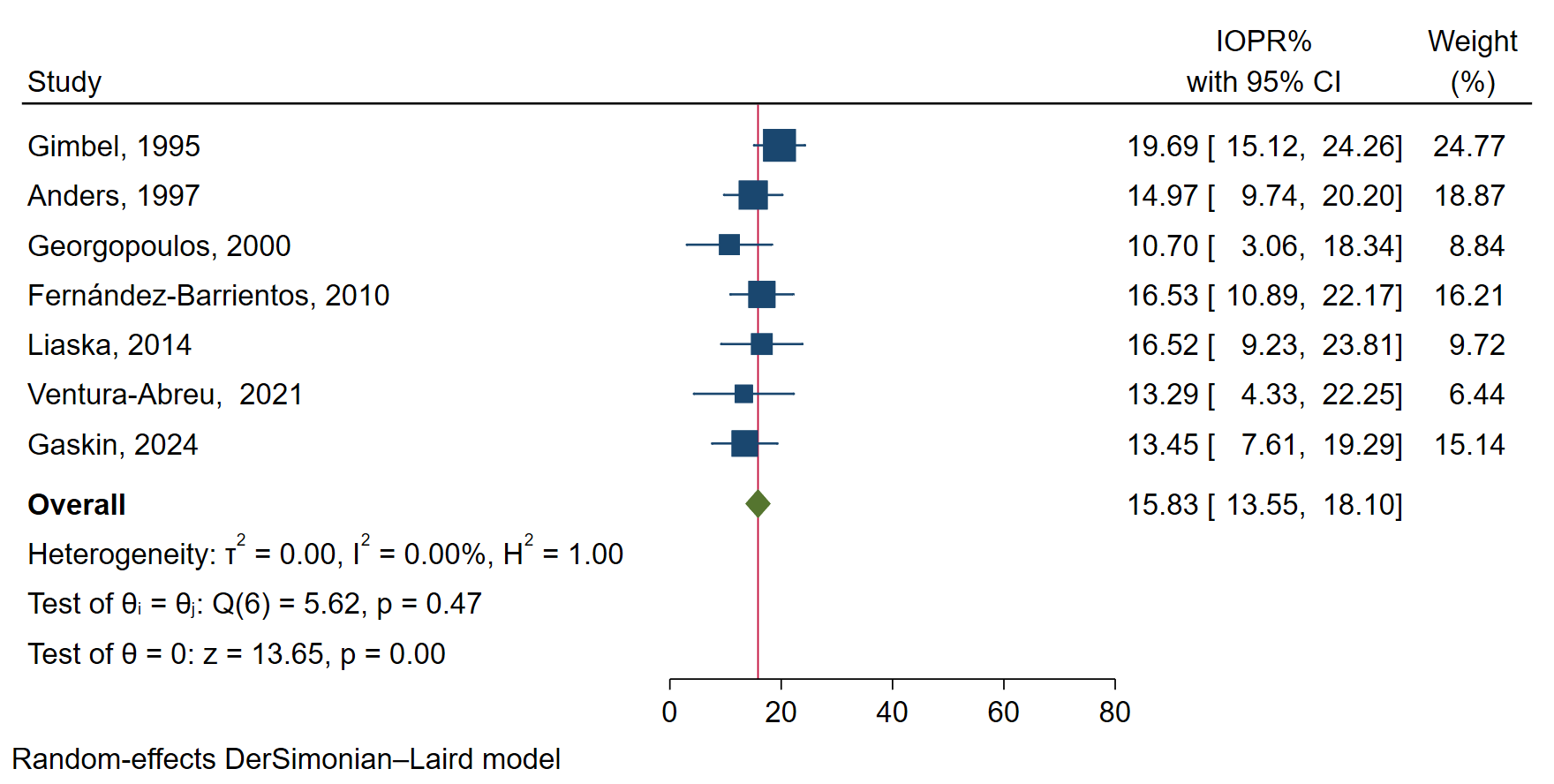
*

**Fig. S36.** Forest plot of 24-month IOPR% in open-angle glaucoma after excluding outliers.


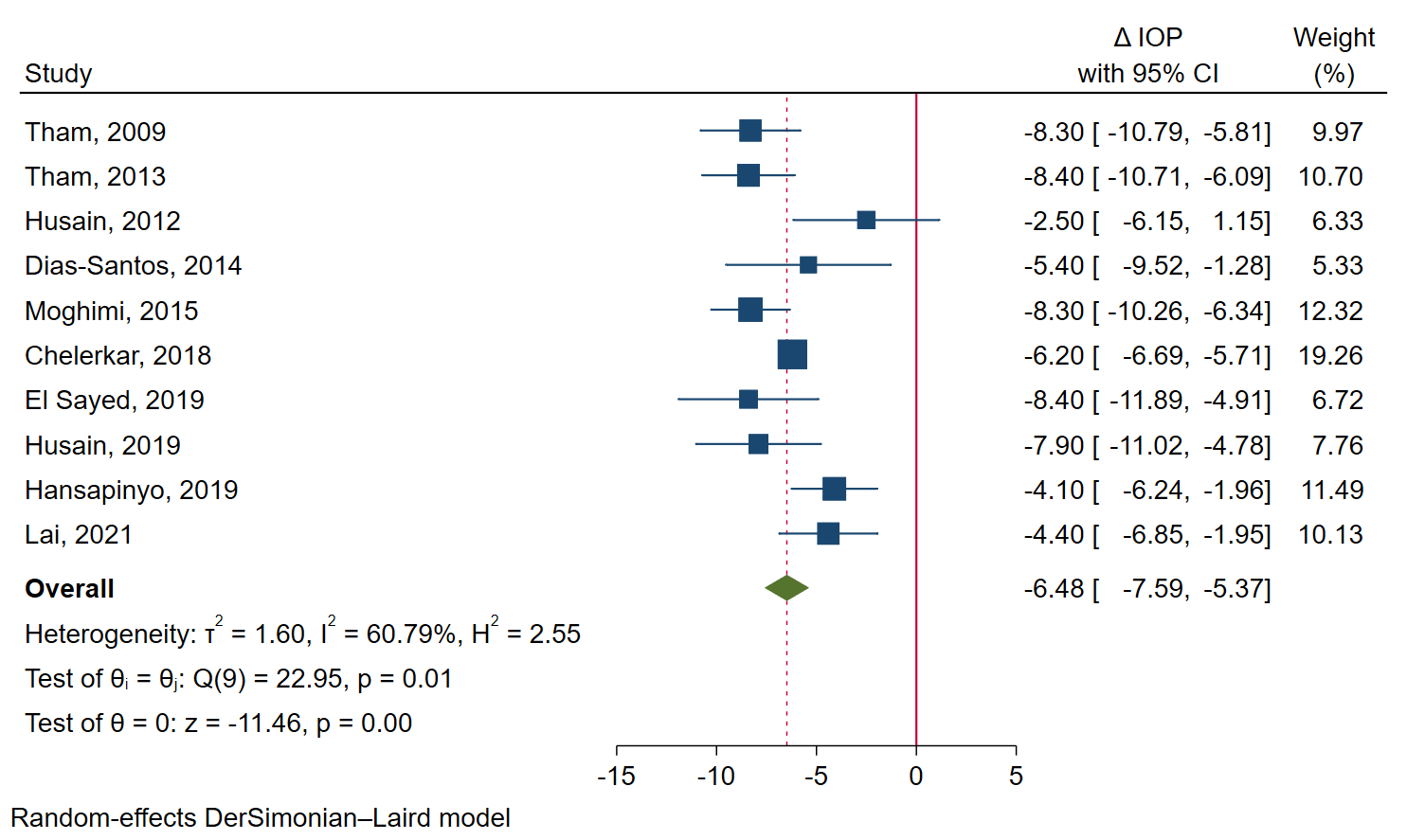


**Fig. S37.** Forest plot of the last follow-up IOP change in closed-angle glaucoma after excluding outliers.


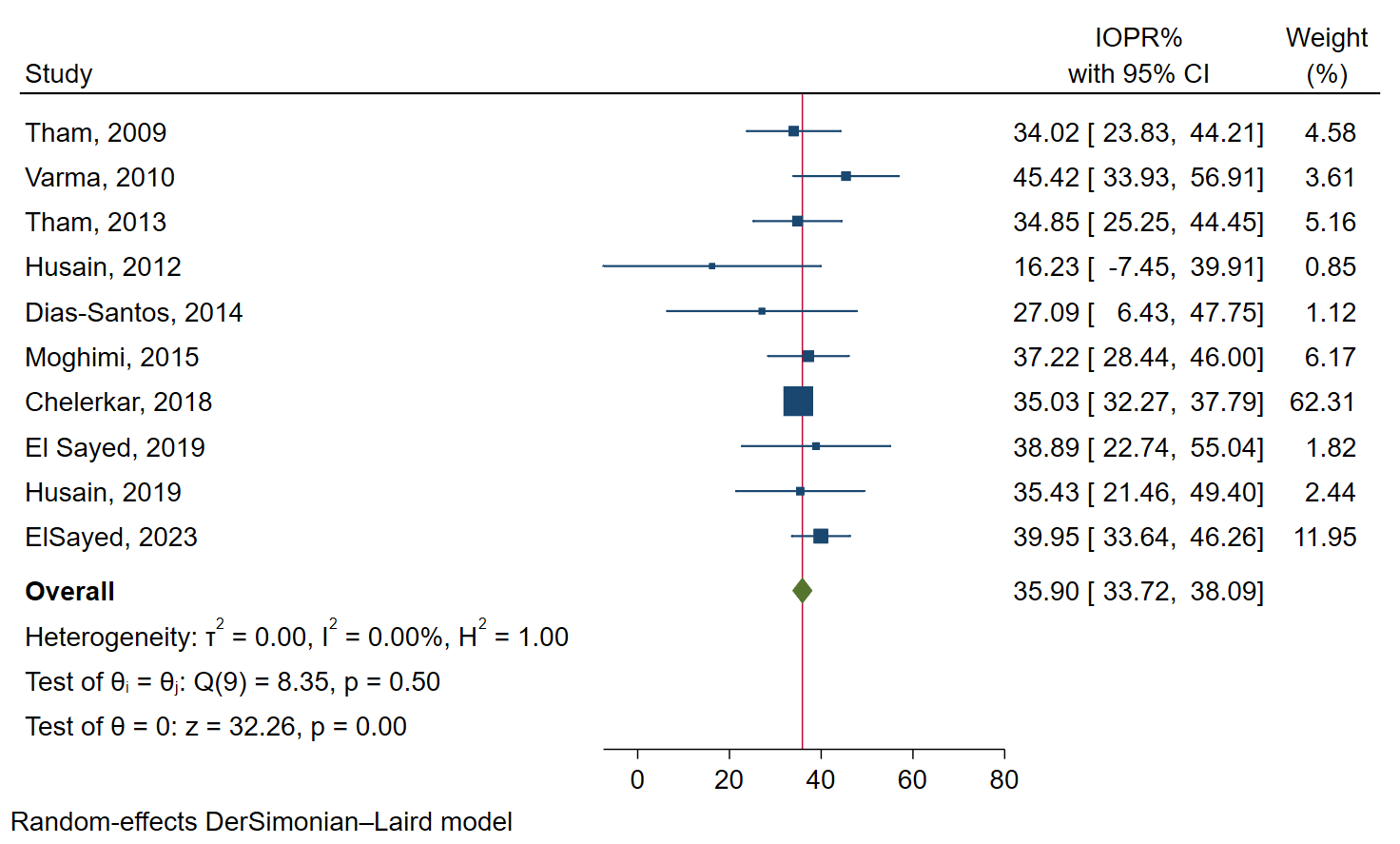


**Fig. S38.** Forest plot of the last follow-up IOPR% in closed-angle glaucoma after excluding outliers.


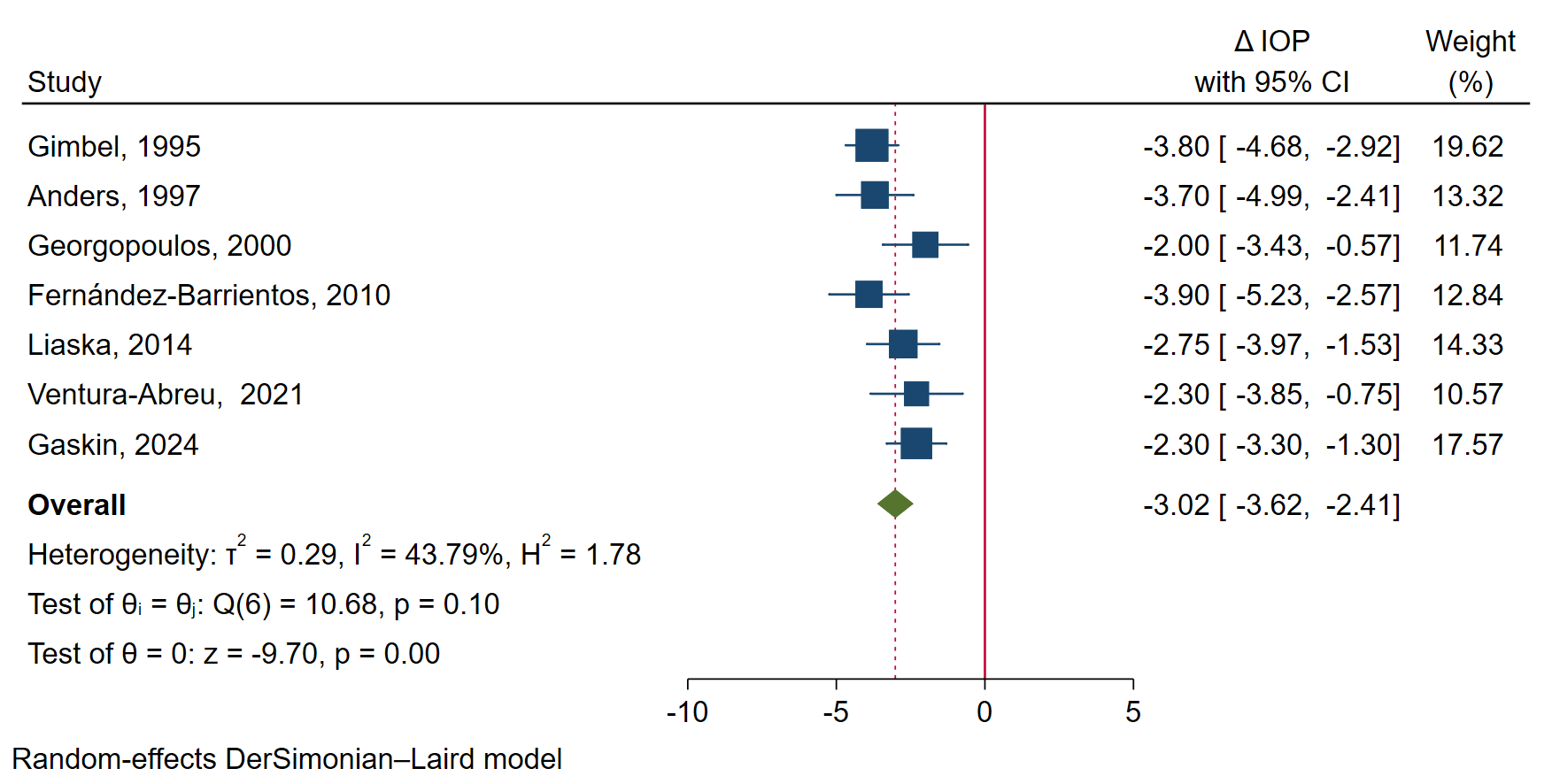


**Fig. S39.** Forest plot of the last follow-up IOP change in open-angle glaucoma after excluding outliers.


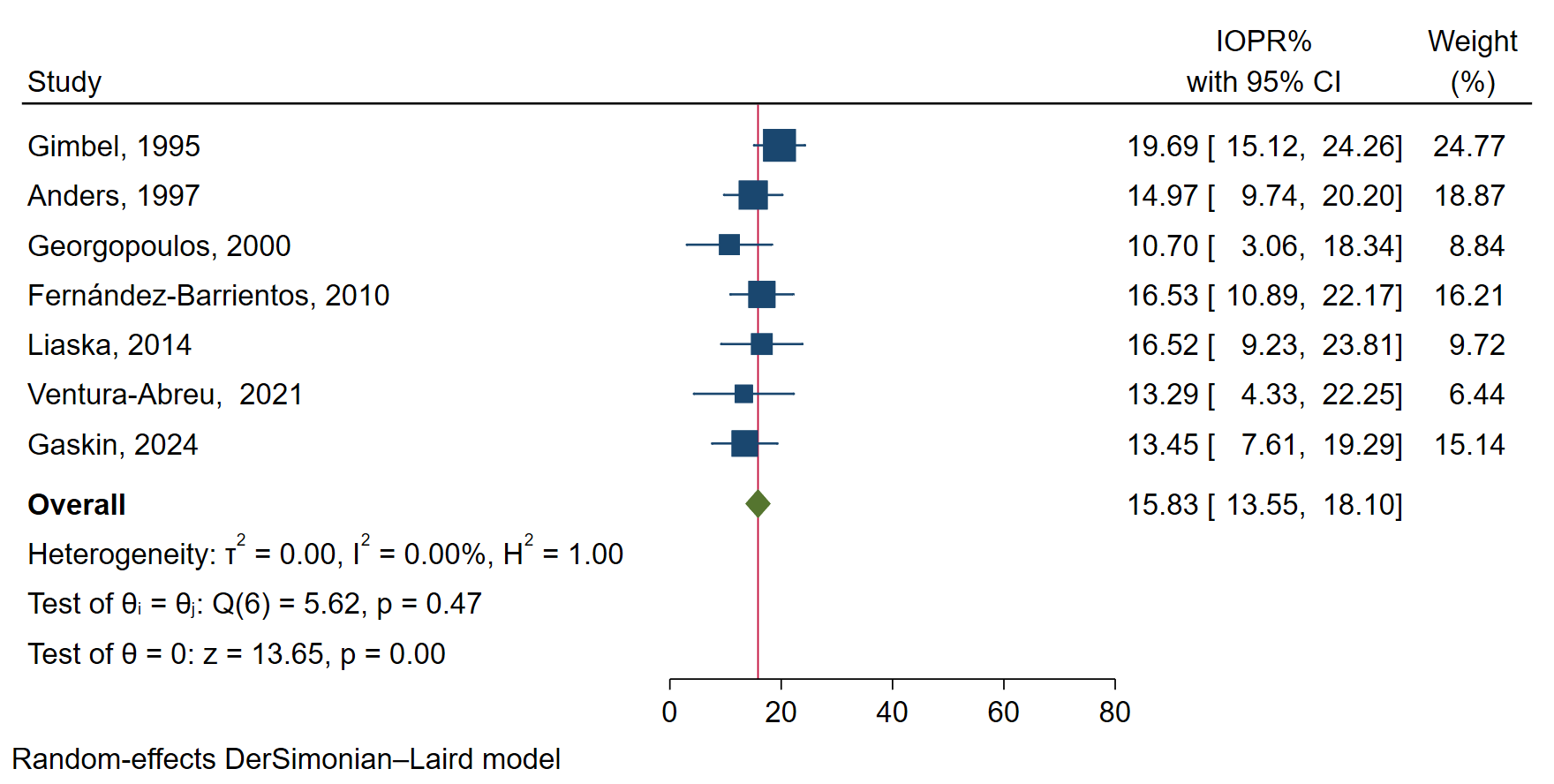


**Fig. S40.** Forest plot of the last follow-up IOPR% in open-angle glaucoma after excluding outliers.

**Subgroup analysis based on Presurgical IOP:**


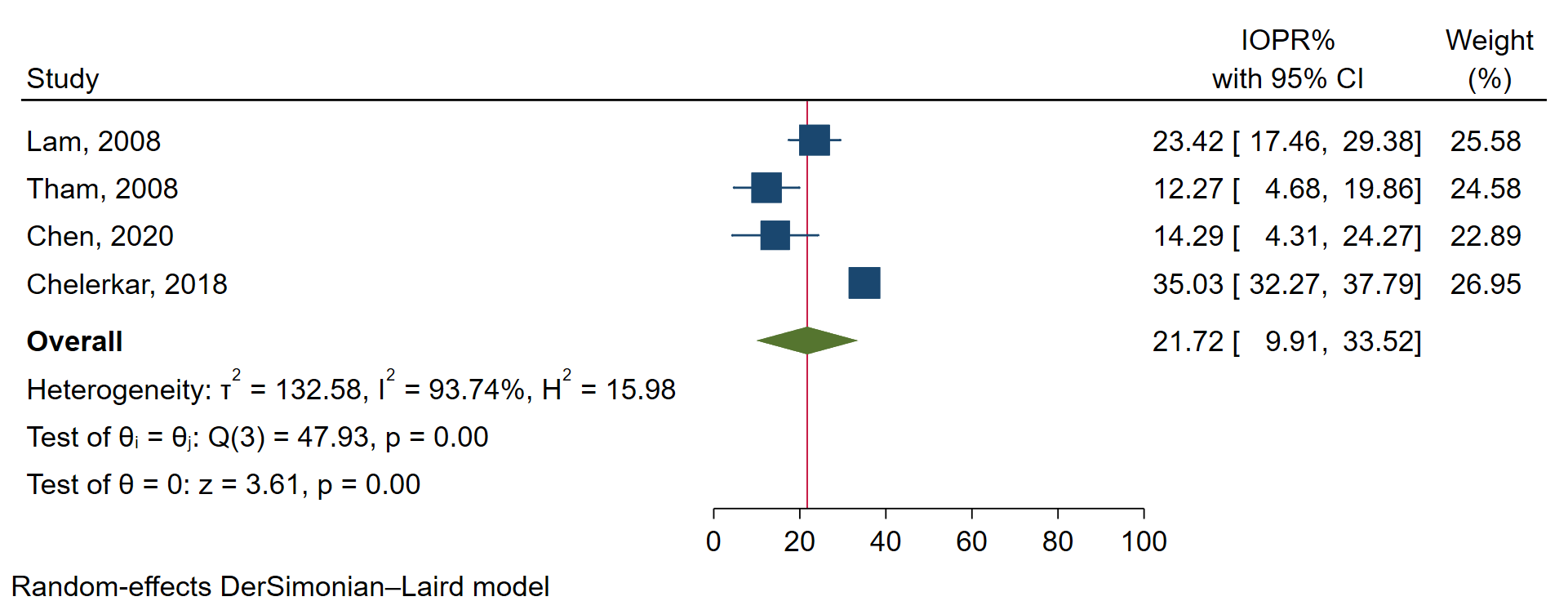


**Fig. S41.** Forest plot of the percentage of IOP reduction for 15-17 presurgical IOP in patients with closed-angle glaucoma at 12 months follow-up period


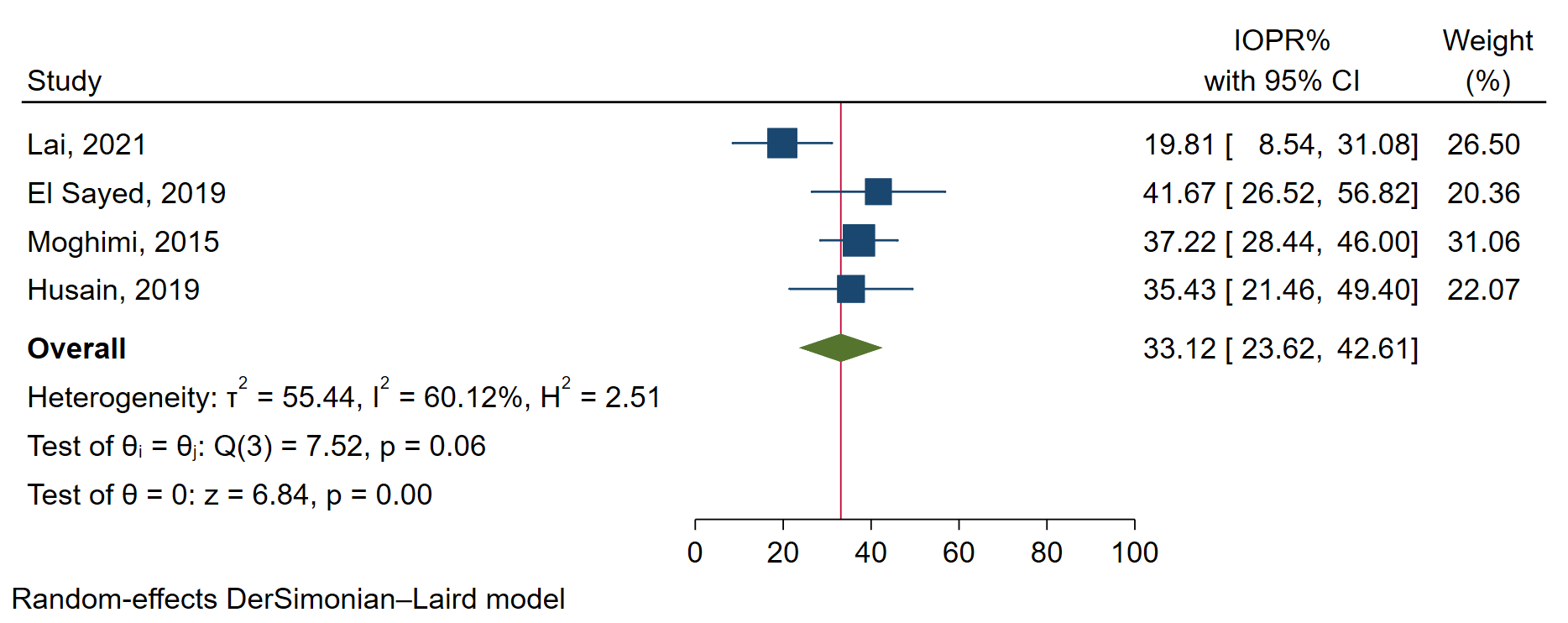


**Fig. S42.** Forest plot of the percentage of IOP reduction for 20-22 presurgical IOP in patients with closed-angle glaucoma at 12 months follow-up period


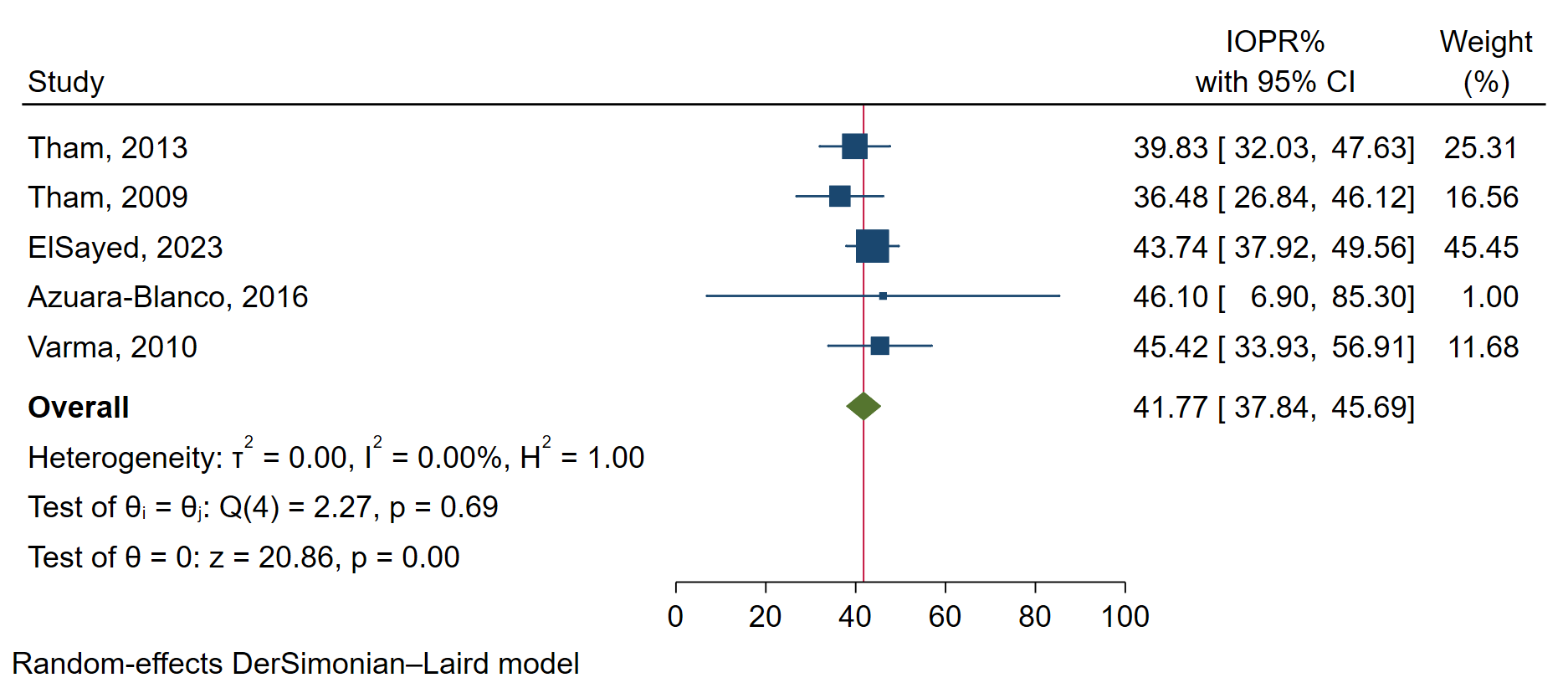


**Fig. S43.** Forest plot of the percentage of IOP reduction for 23-31 presurgical IOP in patients with closed-angle glaucoma at 12 months follow-up period


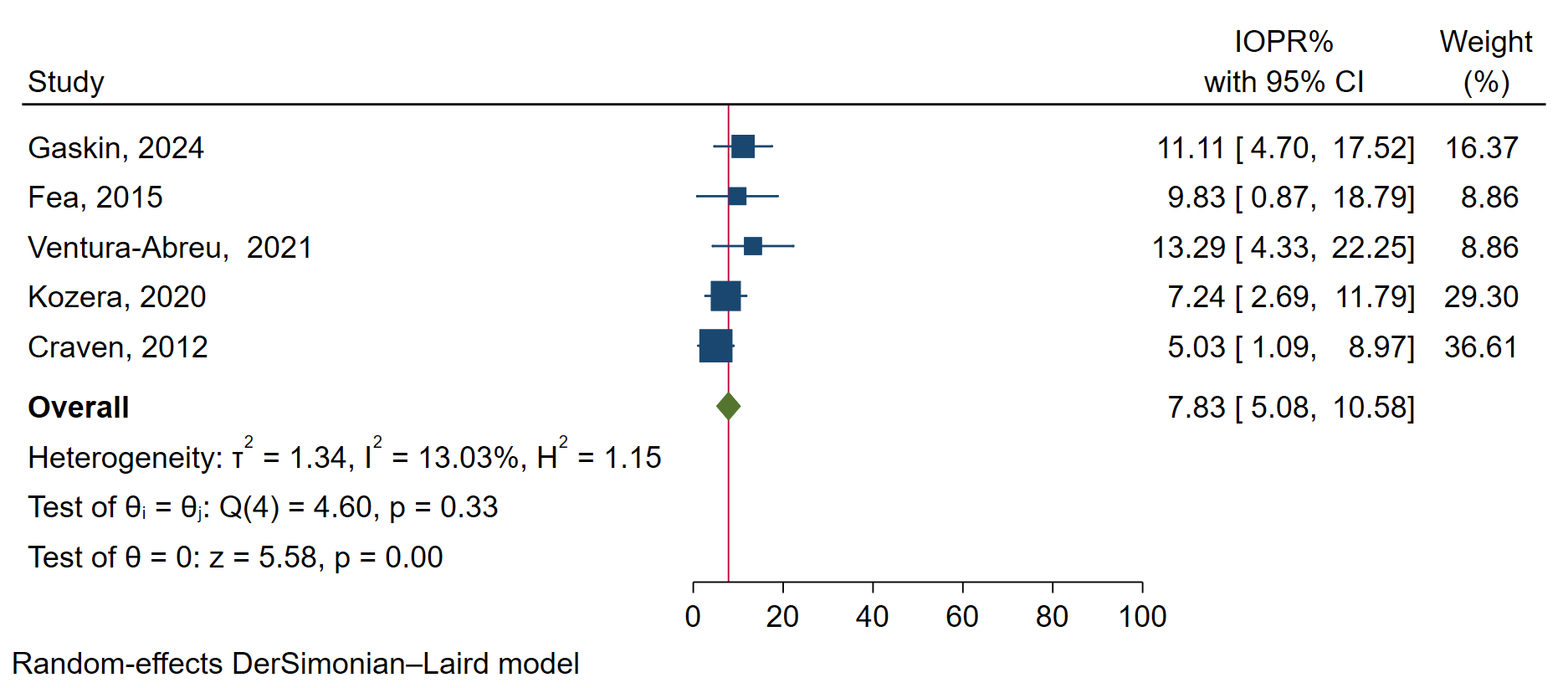


**Fig. S44.** Forest plot of the percentage of IOP reduction for 15-17 presurgical IOP in patients with open-angle glaucoma at 12 months follow-up period


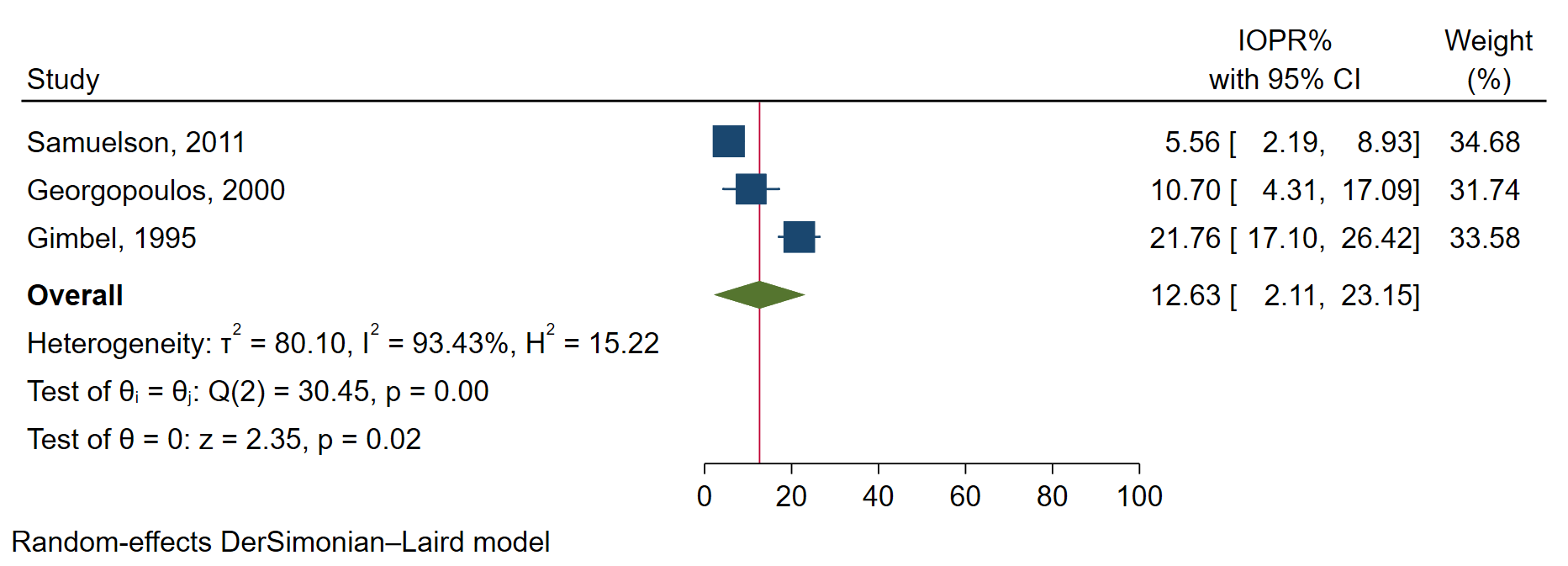


**Fig. S45.** Forest plot of the percentage of IOP reduction for 18-19 presurgical IOP in patients with open-angle glaucoma at 12 months follow-up period


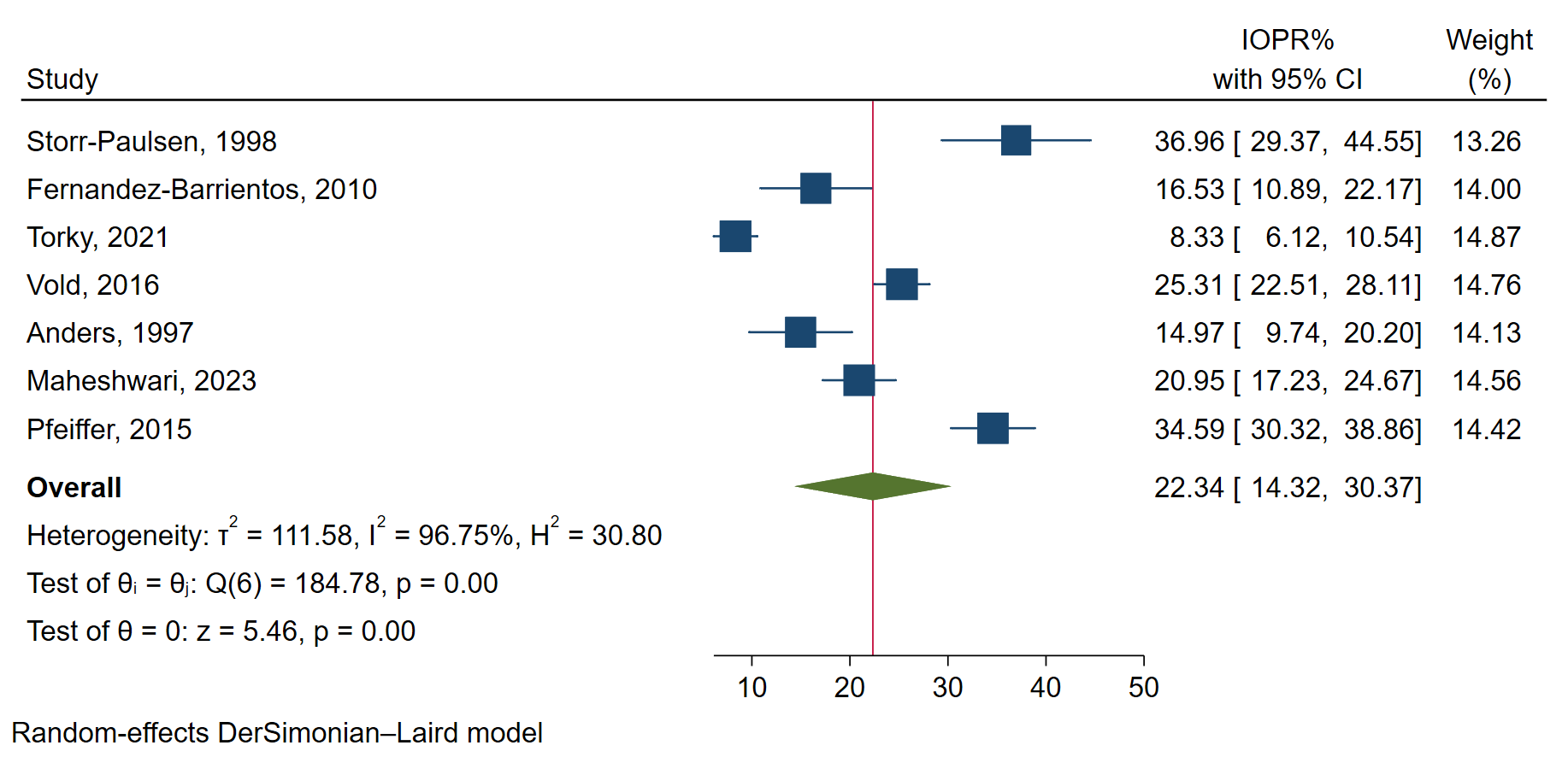


**Fig. S46.** Forest plot of the percentage of IOP reduction for 23-31 presurgical IOP in patients with open-angle glaucoma at 12 months follow-up period


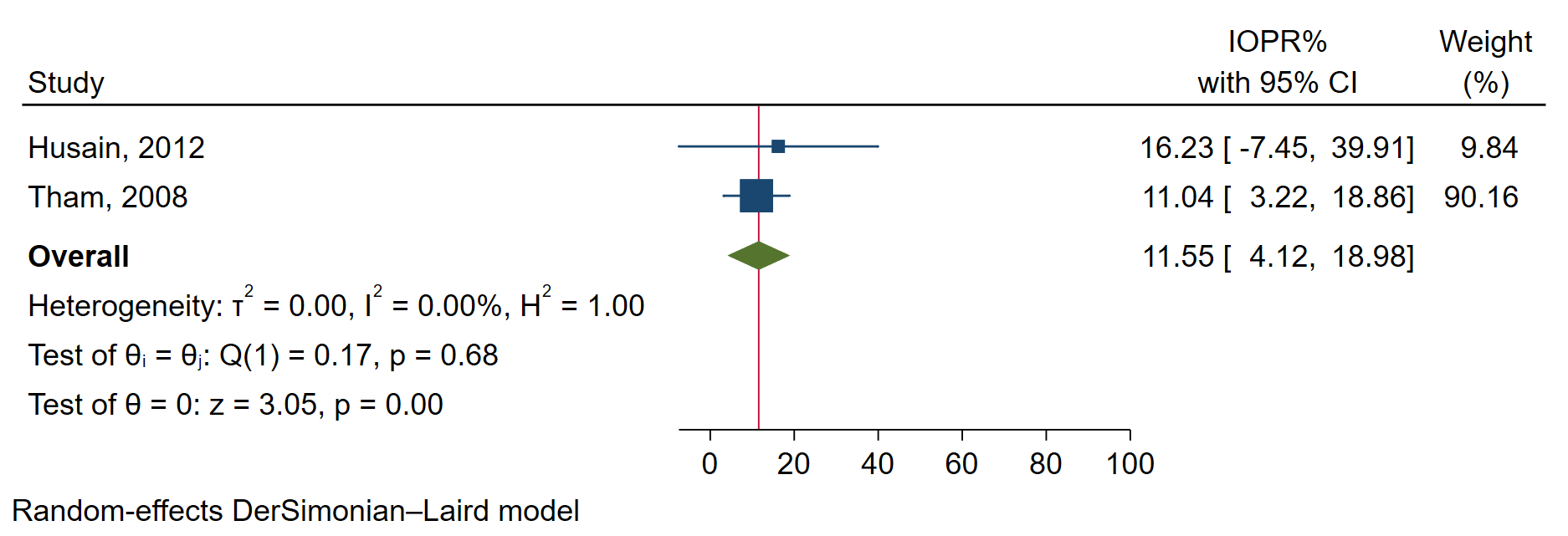


**Fig. S47.** Forest plot of the percentage of IOP reduction for 15-17 presurgical IOP in patients with closed-angle glaucoma at 24 months follow-up period


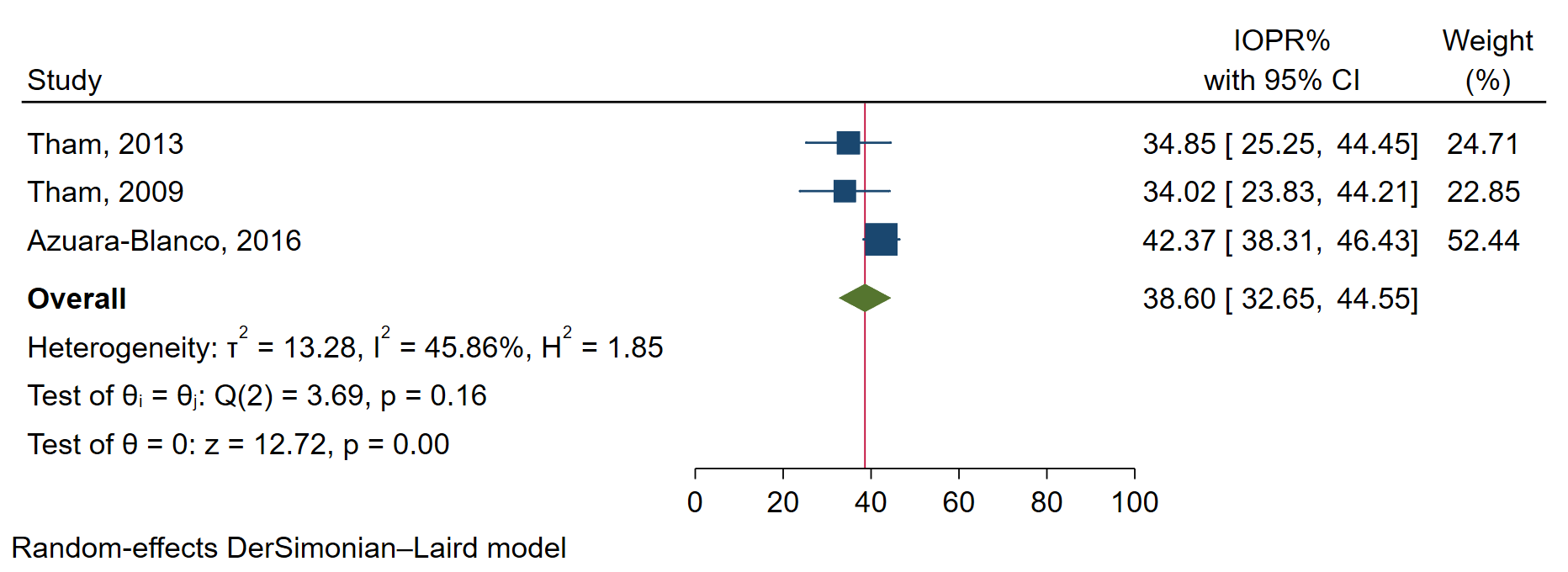


**Fig. S48.** Forest plot of the percentage of IOP reduction for 23-31 presurgical IOP in patients with closed-angle glaucoma at 24 months follow-up period


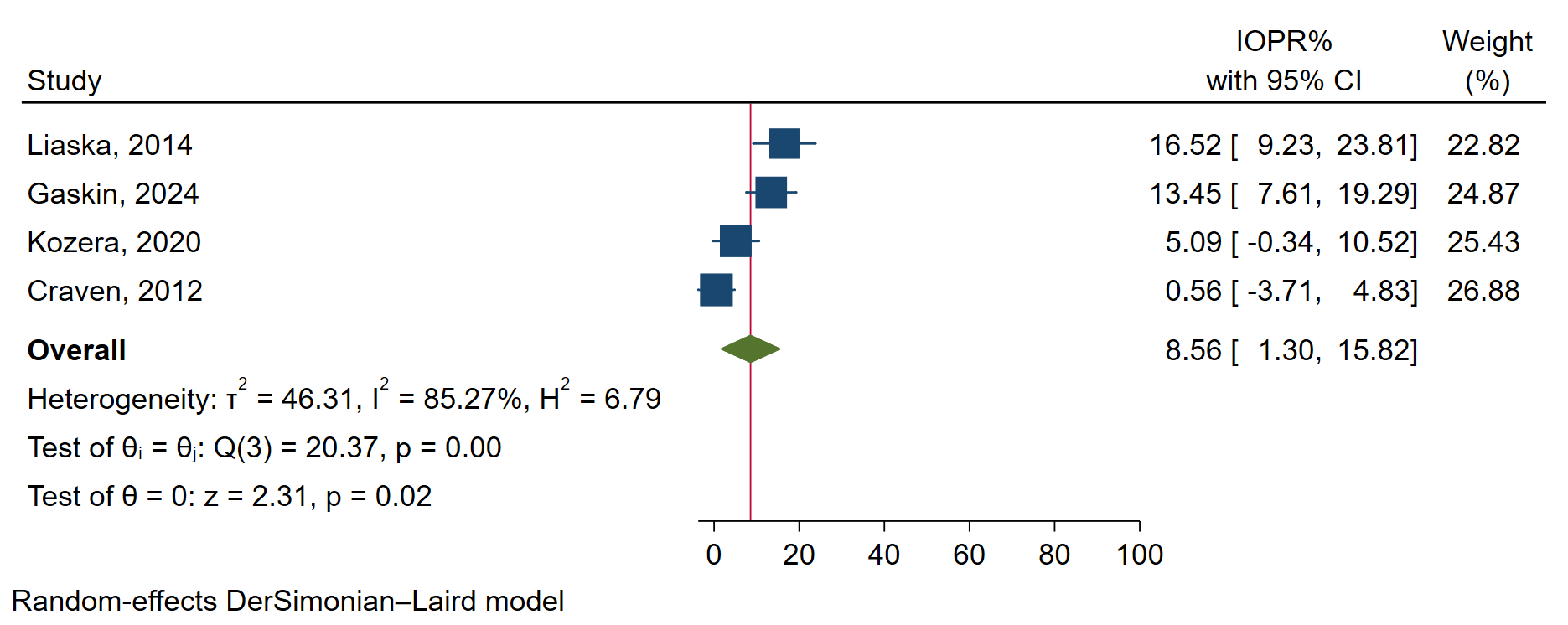


**Fig. S49.** Forest plot of the percentage of IOP reduction for 15-17 presurgical IOP in patients with open-angle glaucoma at 24 months follow-up period


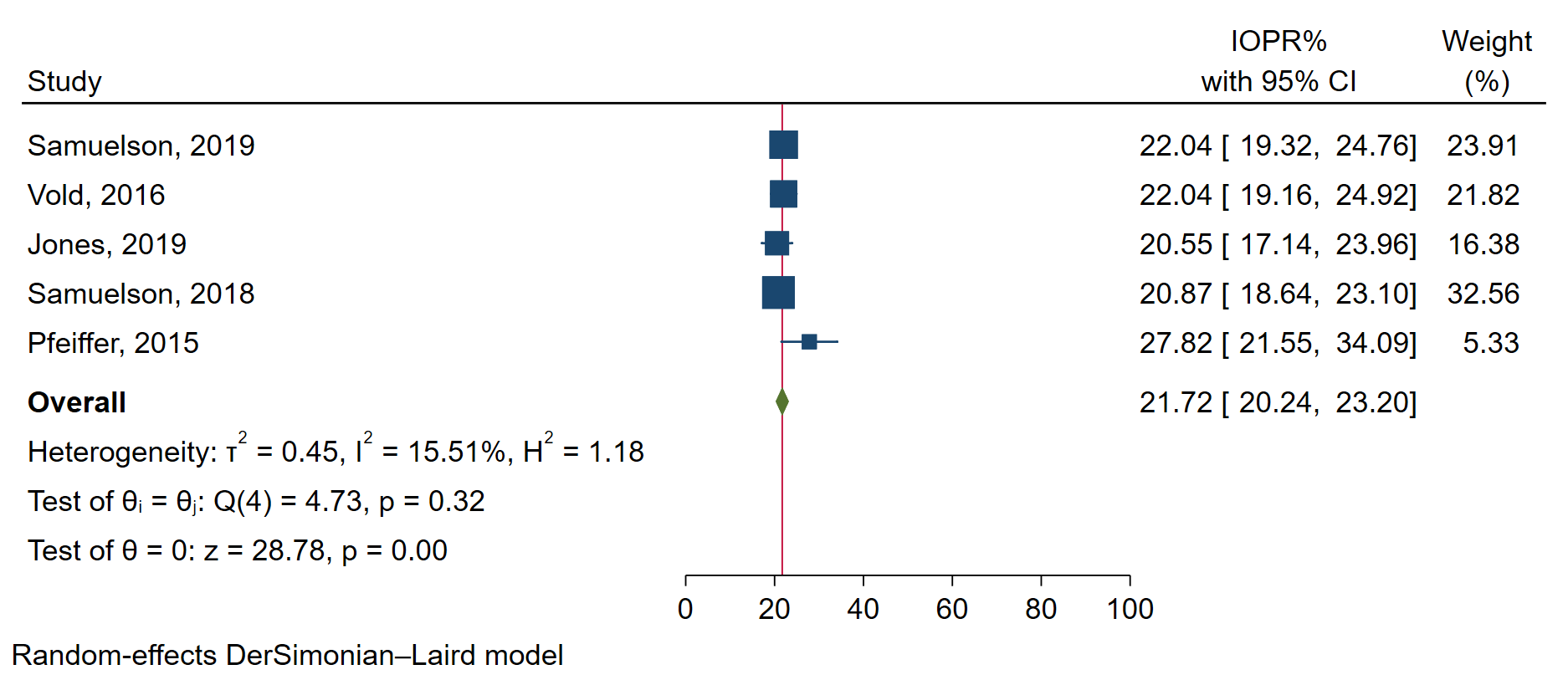


**Fig. S50.** Forest plot of the percentage of IOP reduction for 23-31 presurgical IOP in patients with open-angle glaucoma at 24 months follow-up period


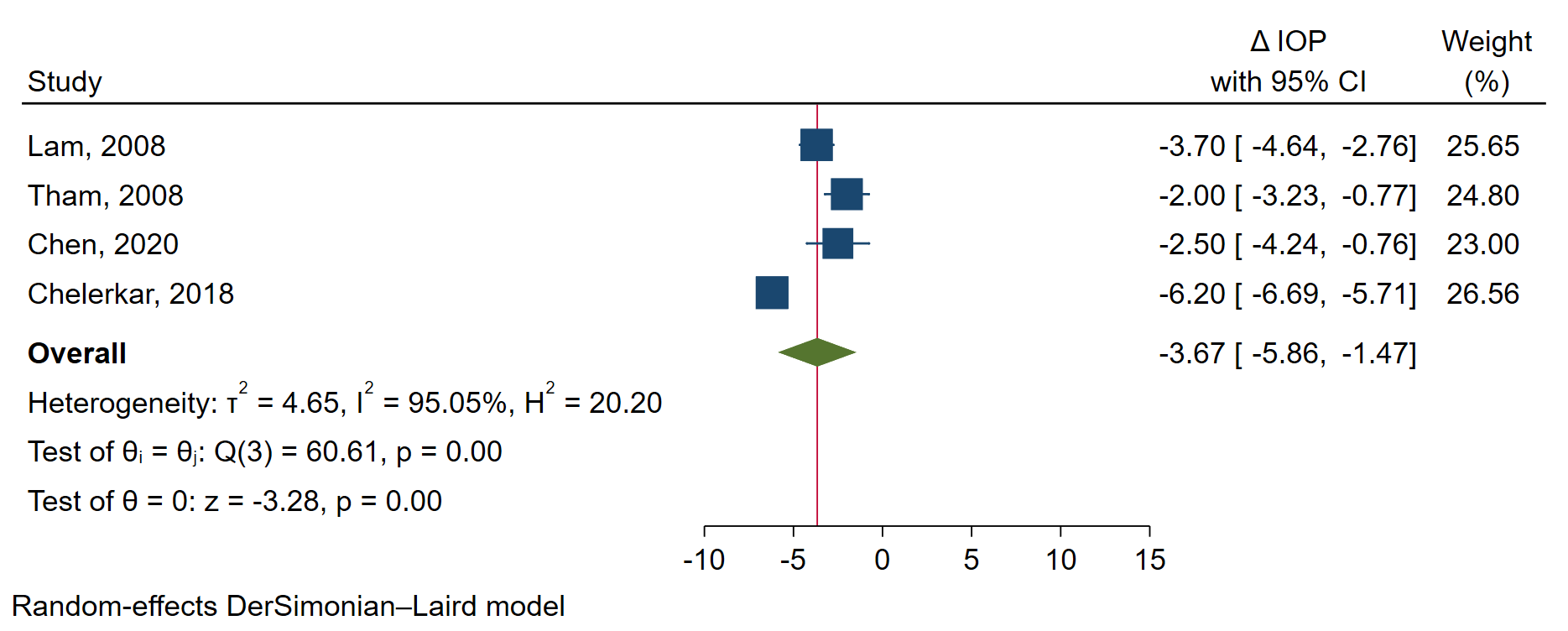


**Fig. S51.** Forest plot of the changes in IOP for 15-17 presurgical IOP in patients with closed-angle glaucoma at 12 months follow-up period


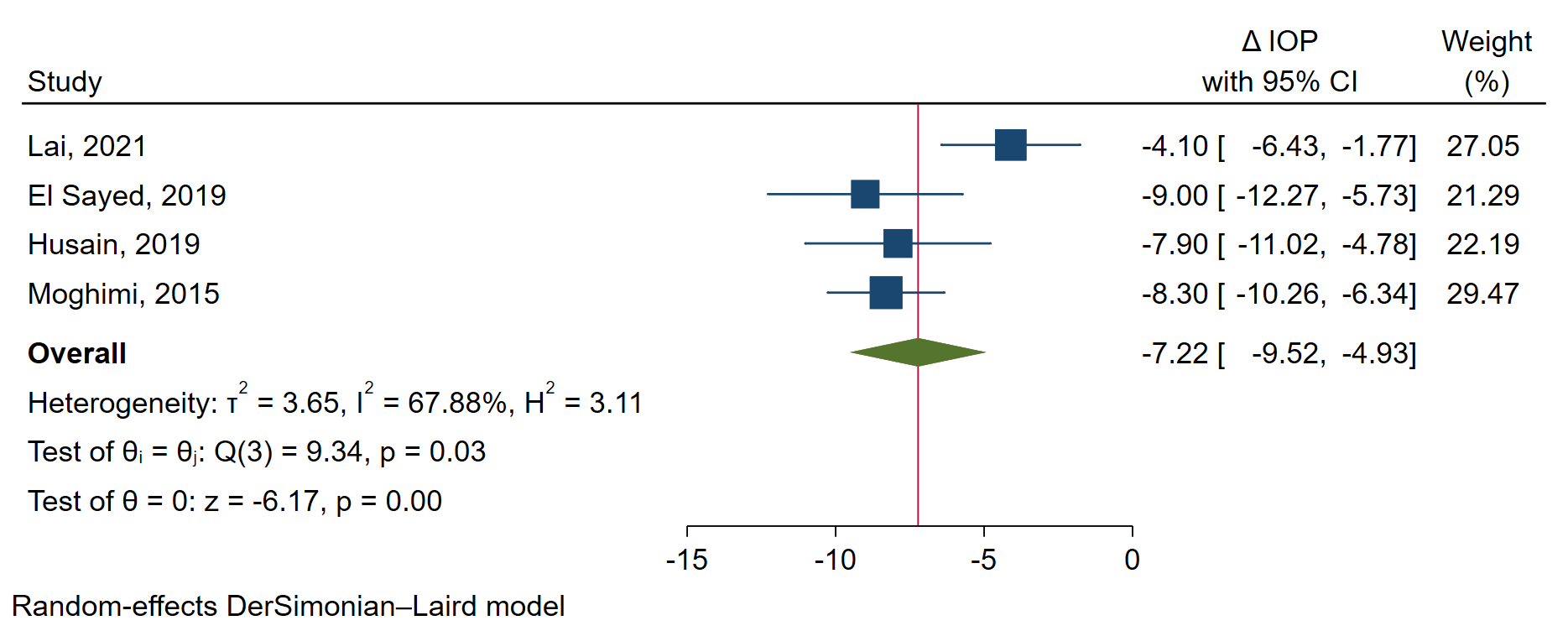


**Fig. S52.** Forest plot of the changes in IOP for 20-22 presurgical IOP in patients with closed-angle glaucoma at 12 months follow-up period


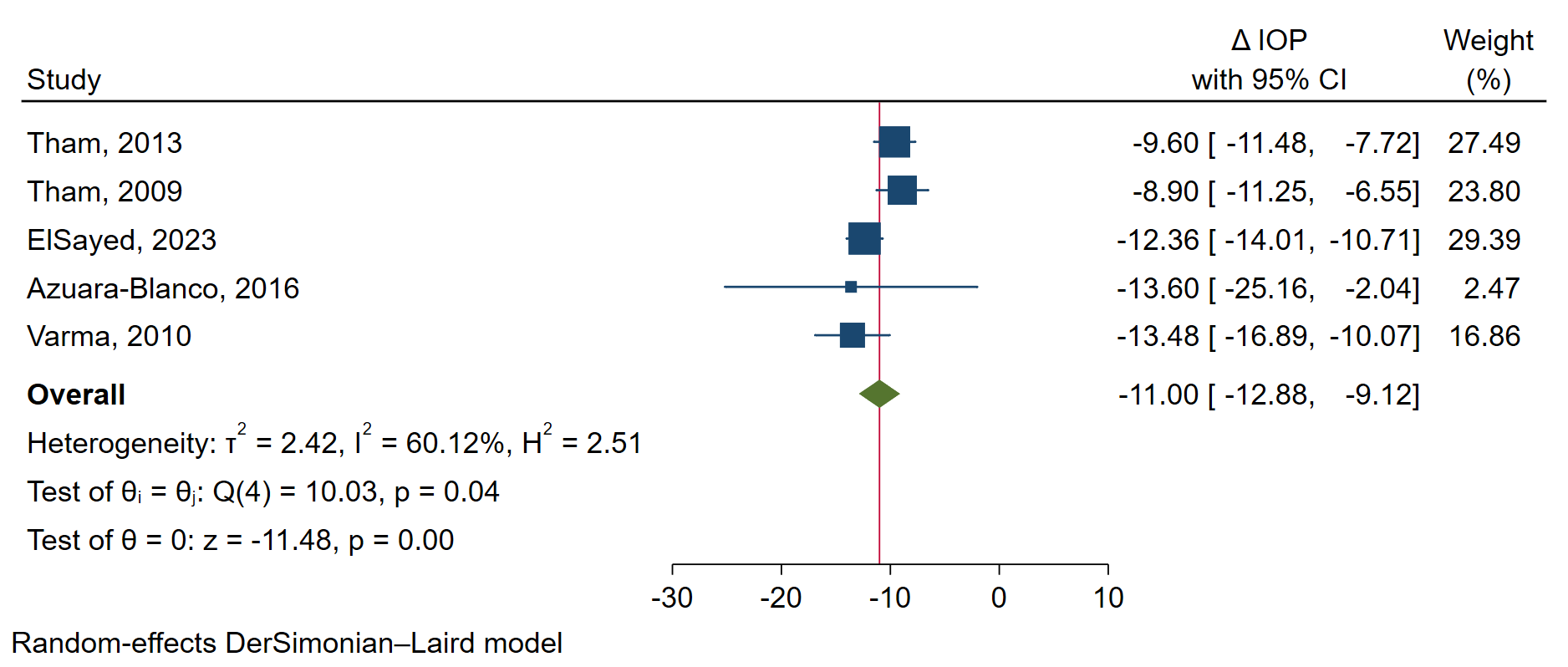


**Fig. S53.** Forest plot of the changes in IOP for 23-31 presurgical IOP in patients with closed-angle glaucoma at 12 months follow-up period


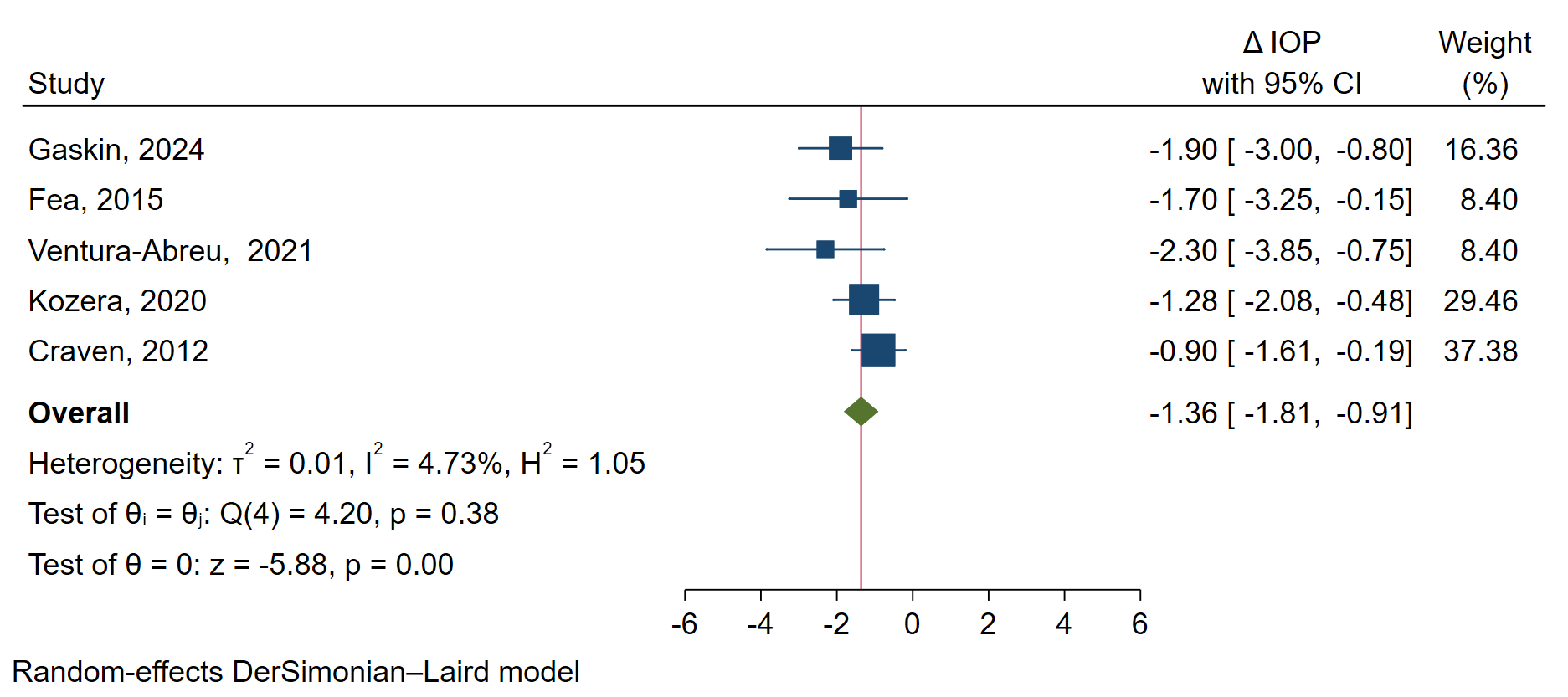


**Fig. S54.** Forest plot of the changes in IOP for 15-17 presurgical IOP in patients with open-angle glaucoma at 12 months follow-up period


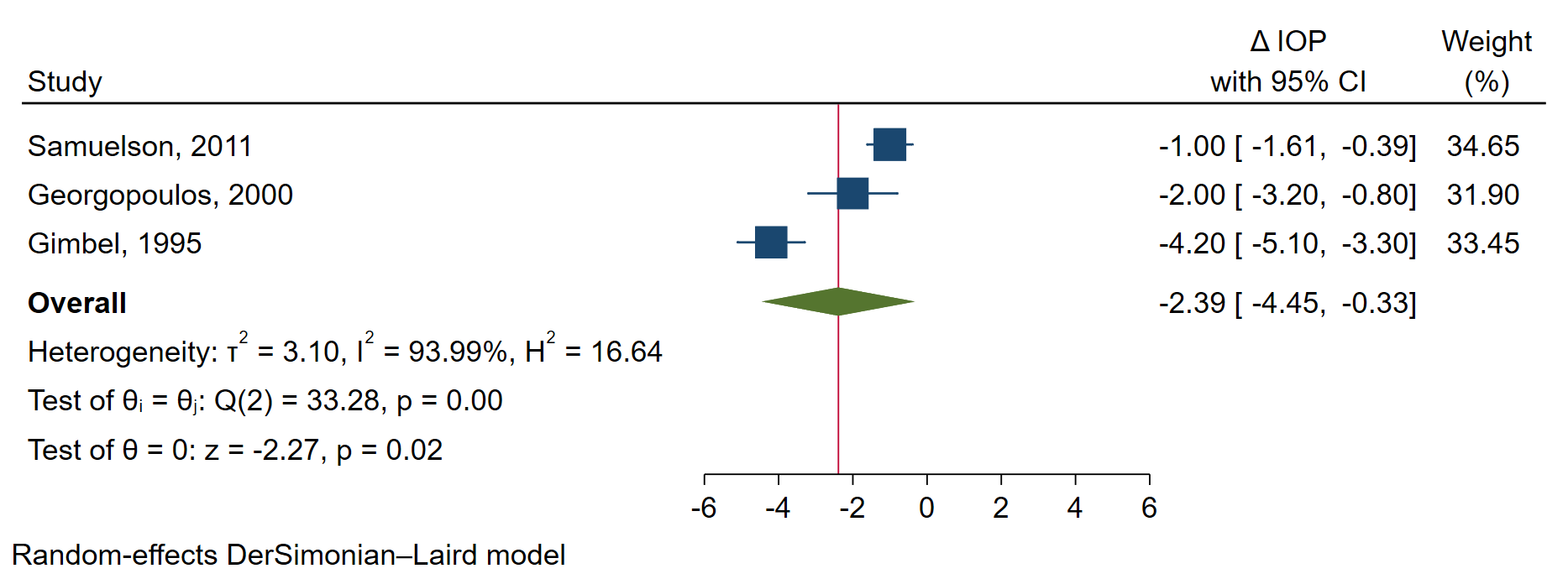


**Fig. S55.** Forest plot of the changes in IOP for 18-19 presurgical IOP in patients with open-angle glaucoma at 12 months follow-up period


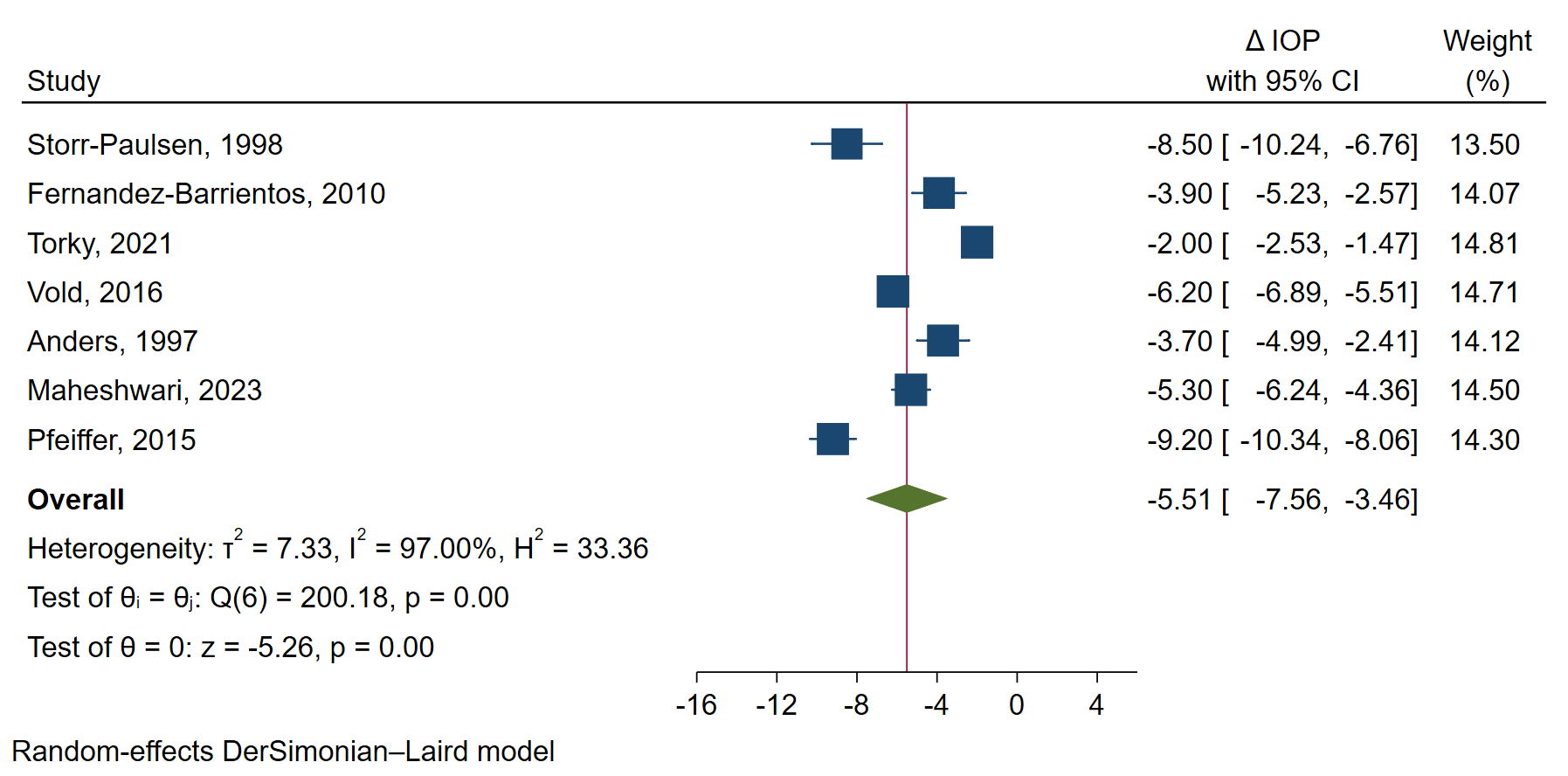


**Fig. S56.** Forest plot of the changes in IOP for 23-31 presurgical IOP in patients with open-angle glaucoma at 12 months follow-up period


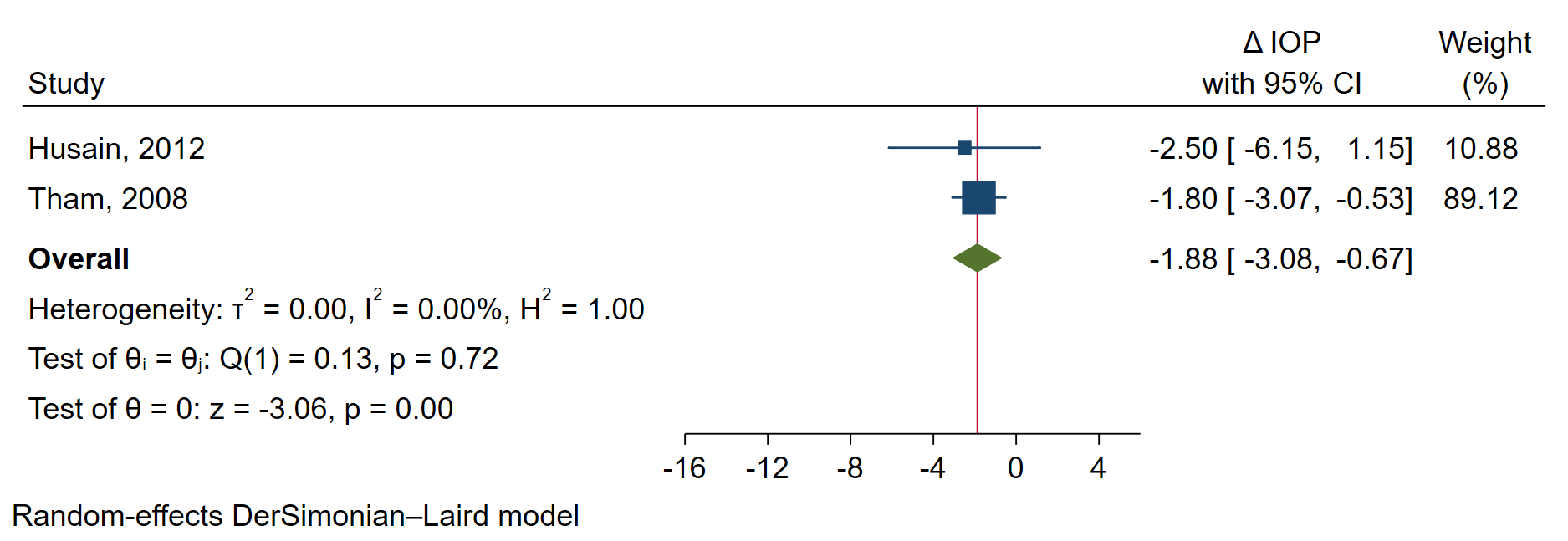


**Fig. S57.** Forest plot of the changes in IOP for 15-17 presurgical IOP in patients with closed-angle glaucoma at 24 months follow-up period


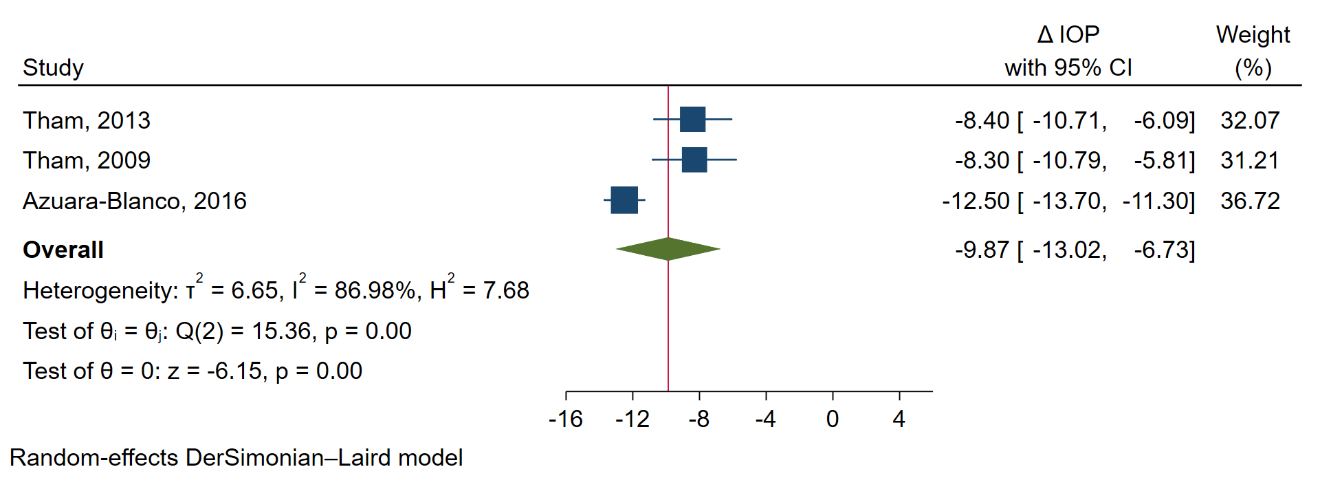


**Fig. S58.** Forest plot of the changes in IOP for 23-31 presurgical IOP in patients with closed-angle glaucoma at 24 months follow-up period


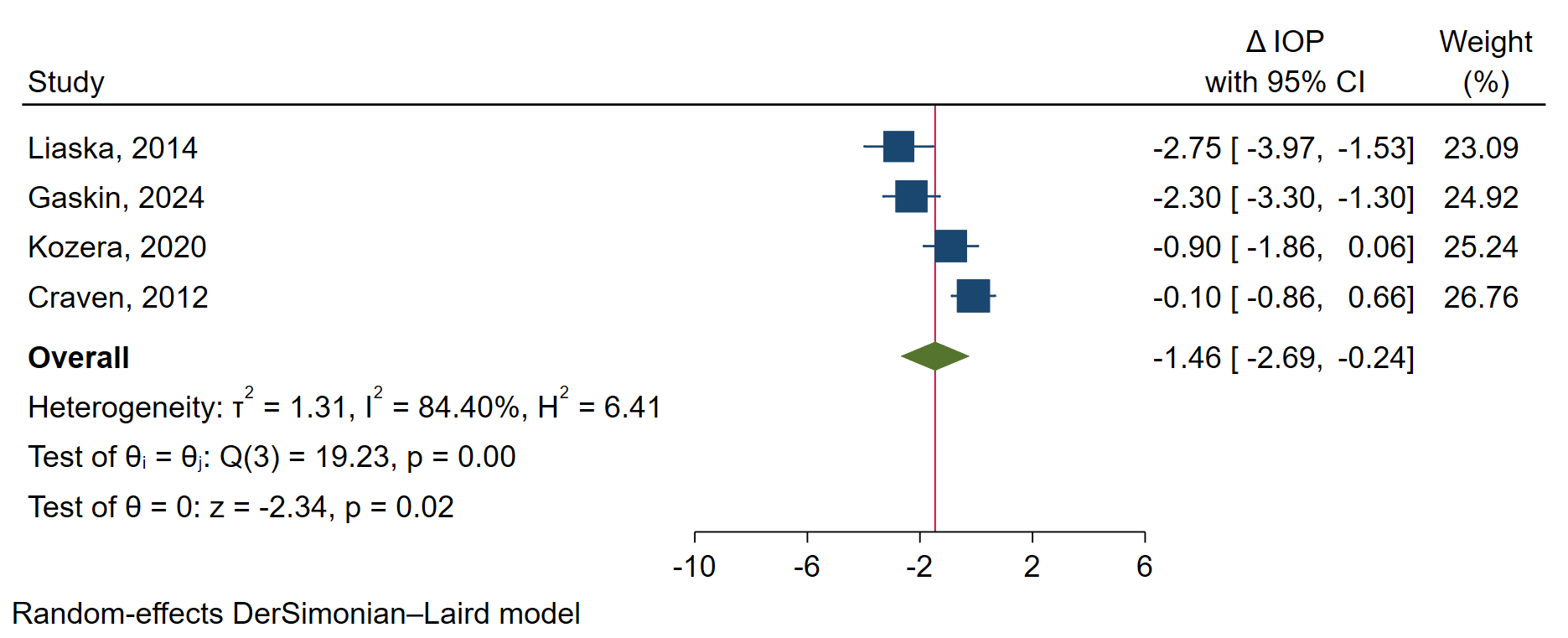


**Fig. S59.** Forest plot of the changes in IOP for 15-17 presurgical IOP in patients with open-angle glaucoma at 24 months follow-up period


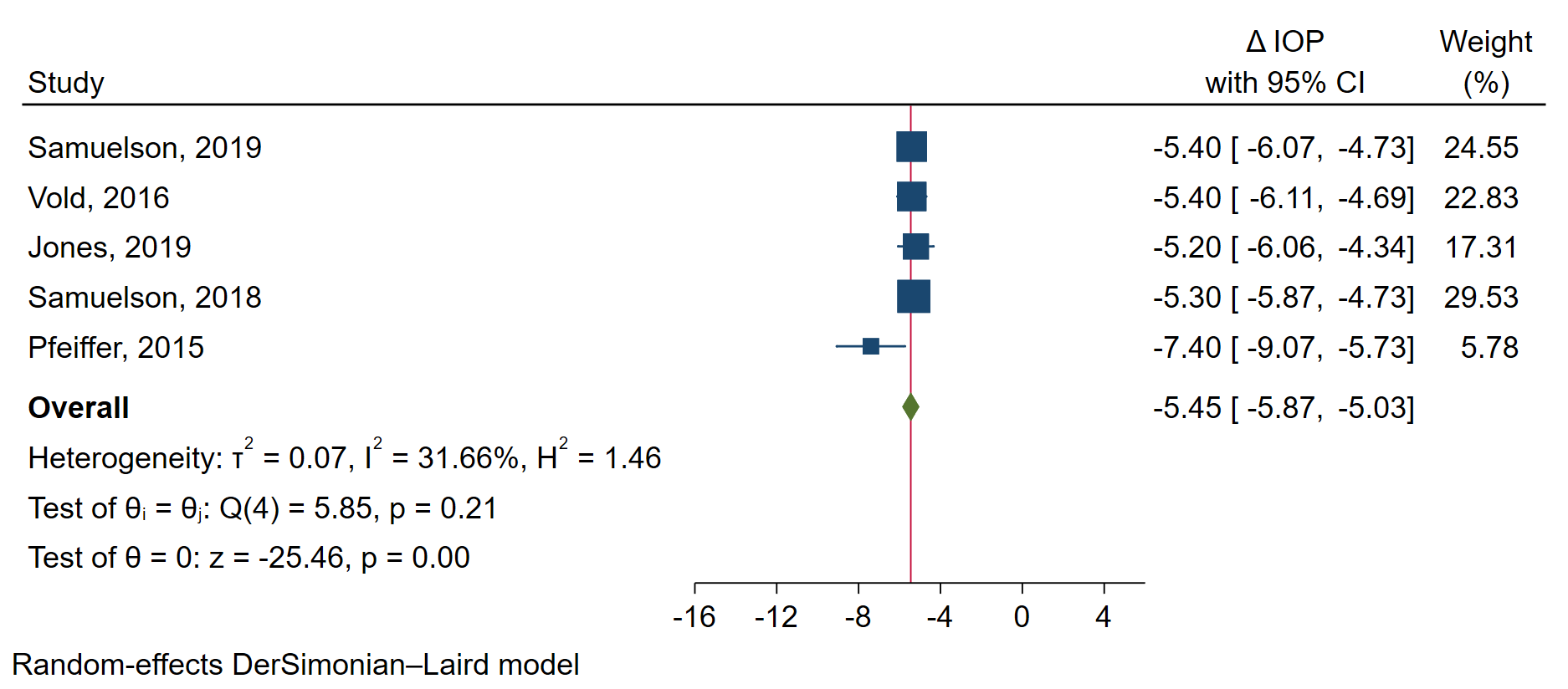


**Fig. S60.** Forest plot of the changes in IOP for 23-31 presurgical IOP in patients with open-angle glaucoma at 24 months follow-up period

**Publication bias:**

**Closed-angle at 12 months follow-up:**

1. Regression-based Egger test for small-study effects

Random-effects model

Method: DerSimonian–Laird: H0: beta1 = 0; no small-study effects

- beta1 = -2.65
- SE of beta1 = 1.449
- z = -1.83
- Prob > |z| = 0.0670

2. Begg's test for small-study effects

- Kendall's score = -27.00
- SE of score = 18.267
- z = -1.53
- Prob > |z| = 0.1546

3. Nonparametric trim-and-fill analysis of publication bias

| Studies | Δ IOP | [95% conf. interval] |
| --- | --- | --- |
| Observed | -9.506 | -12.221 -6.792 |
| Observed + Imputed | -9.506 | -12.221 -6.792 |

**Open-angle at 12 months follow-up:**

1. Regression-based Egger test for small-study effects

Random-effects model

Method: DerSimonian–Laird: H0: beta1 = 0; no small-study effects

- beta1 = -6.72
- SE of beta1 = 1.943
- z = -3.46
- Prob > |z| = 0.0005

1. Begg's test for small-study effects

- Kendall's score = -47.00
- SE of score = 24.256
- z = -1.98
- Prob > |z| = 0.0579

1. Nonparametric trim-and-fill analysis of publication bias

| Studies | Δ IOP | [95% conf. interval] |
| --- | --- | --- |
| Observed | -4.347 | -5.662 -3.032 |
| Observed + Imputed | -4.347 | -5.662 -3.032 |

**Closed-angle at 24 months follow-up:**

1. Regression-based Egger test for small-study effects

Random-effects model

Method: DerSimonian–Laird: H0: beta1 = 0; no small-study effects

- beta1 = 3.38
- SE of beta1 = 5.783
- z = 0.58
- Prob > |z| = 0.5594

1. Begg's test for small-study effects

- Kendall's score = 5.00
- SE of score = 5.323
- z = 0.75
- Prob > |z| = 0.4524

1. Nonparametric trim-and-fill analysis of publication bias

| Studies | Δ IOP | [95% conf. interval] |
| --- | --- | --- |
| Observed | -6.369 | -10.738 -1.999 |
| Observed + Imputed | -6.369 | -10.738 -1.999 |

**Open-angle at 24 months follow-up:**

1. Regression-based Egger test for small-study effects

Random-effects model

- Method: DerSimonian–Laird: H0: beta1 = 0; no small-study effects
- beta1 = -2.49
- SE of beta1 = 4.672
- z = -0.53
- Prob > |z| = 0.5947

1. Begg's test for small-study effects

- Kendall's score = 17.00
- SE of score = 11.180
- z = 1.43
- Prob > |z| = 0.1524

1. Nonparametric trim-and-fill analysis of publication bias

| Studies | Δ IOP | [95% conf. interval] |
| --- | --- | --- |
| Observed | -3.833 | -5.178 -2.488 |
| Observed + Imputed | -3.833 | -5.178 -2.488 |


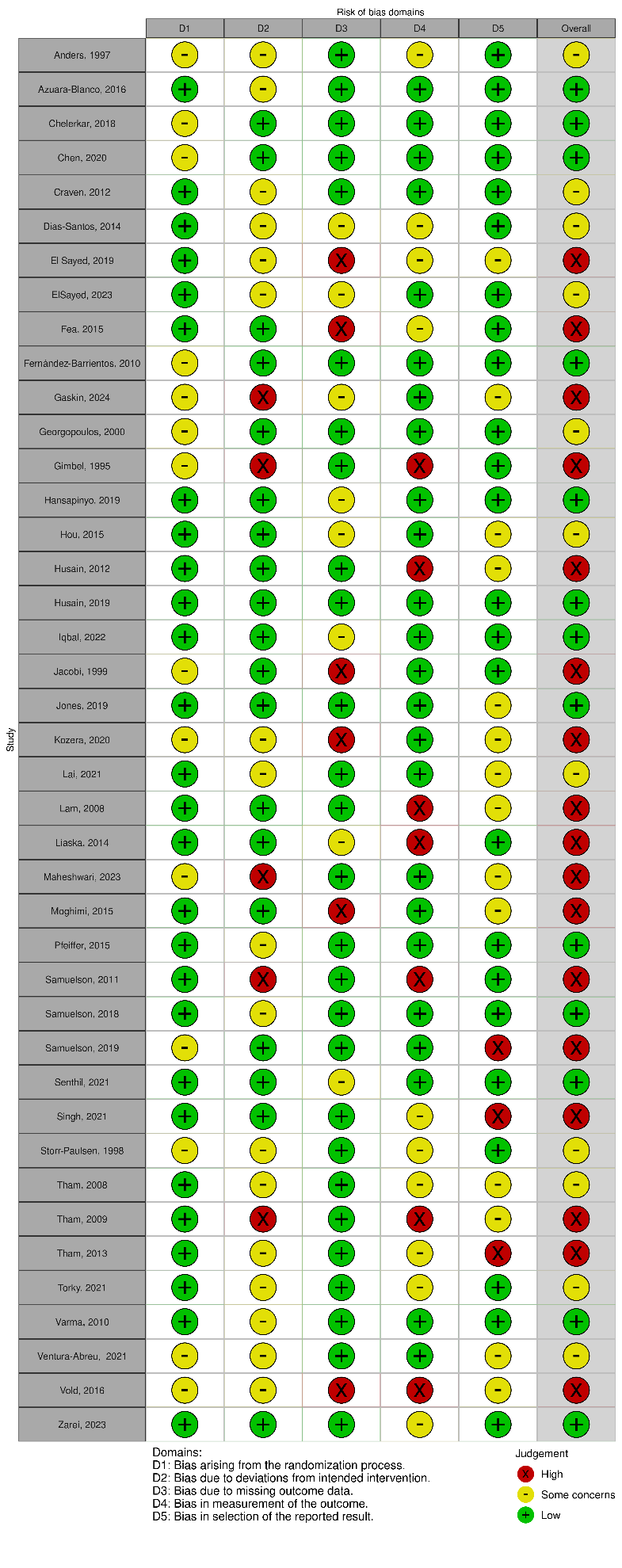

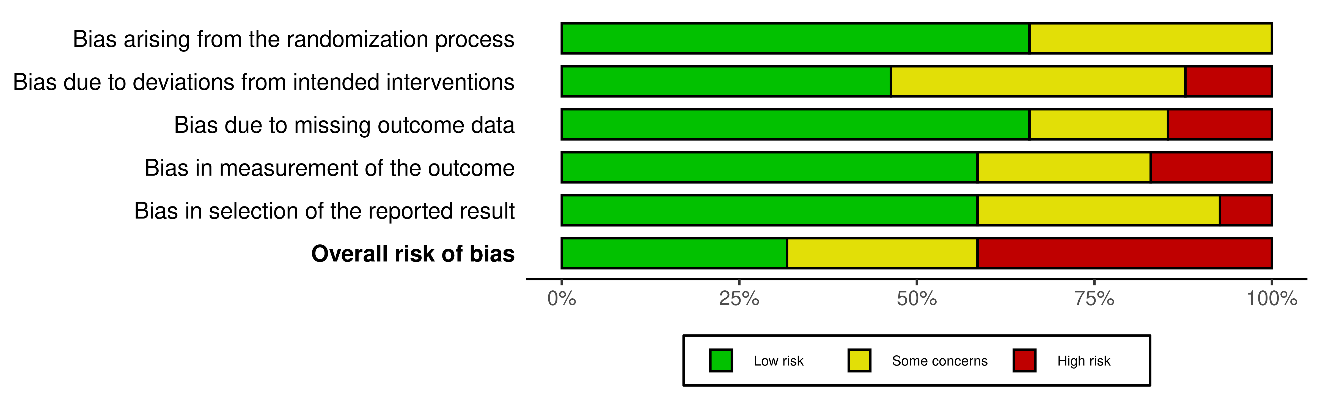


**Fig. S61** Risk of bias summary
